# Angiogenesis and anti-leukaemia activity of novel indole derivatives as potent colchicine binding site inhibitors

**DOI:** 10.1080/14756366.2022.2032688

**Published:** 2022-02-02

**Authors:** Yongfang Yao, Tao Huang, Yuyang Wang, Longfei Wang, Siqi Feng, Weyland Cheng, Longhua Yang, Yongtao Duan

**Affiliations:** a Henan Provincial Key Laboratory of Pediatric Hematology, Children's Hospital Affiliated to Zhengzhou University, Zhengzhou University, Zhengzhou, China; b School of Pharmaceutical Sciences, Zhengzhou University, Zhengzhou, Henan, China; c Ministry of Education of China, Key Laboratory of Advanced Drug Preparation Technologies (Zhengzhou University), Zhengzhou, China; d Henan Provincial Key Laboratory of Children's Genetics and Metabolic Diseases, Children's Hospital Affiliated to Zhengzhou University, Zhengzhou University, Zhengzhou, China; e Medical School, Huanghe Science and Technology University, Zhengzhou, Henan Province, P.R China

**Keywords:** Leukaemia, anti-angiogenesis, tubulin, zebrafish

## Abstract

The screened compound DYT-1 from our in-house library was taken as a lead (inhibiting tubulin polymerisation: IC_50_=25.6 µM, anti-angiogenesis in Zebrafish: IC_50_=38.4 µM, anti-proliferation against K562 and Jurkat: IC_50_=6.2 and 7.9 µM, respectively). Further investigation of medicinal chemistry conditions yielded compound **29e** (inhibiting tubulin polymerisation: IC_50_=4.8 µM and anti-angiogenesis in Zebrafish: IC_50_=3.6 µM) based on tubulin and zebrafish assays, which displayed noteworthily nanomolar potency against a variety of leukaemia cell lines (IC_50_= 0.09–1.22 µM), especially K562 cells where apoptosis was induced. Molecular docking, molecular dynamics (MD) simulation, radioligand binding assay and cellular microtubule networks disruption results showed that **29e** stably binds to the tubulin colchicine site. **29e** significantly inhibited HUVEC tube formation, migration and invasion in vitro. Anti-angiogenesis in vivo was confirmed by zebrafish xenograft. **29e** also prominently blocked K562 cell proliferation and metastasis in blood vessels and surrounding tissues of the zebrafish xenograft model. Together with promising physicochemical property and metabolic stability, **29e** could be considered an effective anti-angiogenesis and -leukaemia drug candidate that binds to the tubulin colchicine site.

## 1. Introduction

Leukaemia is a group of life-threatening malignant disorders of the blood and bone marrow which may present at all ages, from the newborn to the elderly. Acute lymphoblastic leukaemia (ALL) is the most common in early childhood and rare in adults whereas acute myeloid leukaemia (AML) is less common than ALL in children but increasingly common in older adults.^1^ Progress in the treatment of leukaemia has been accelerated as a result of a better understanding of the pathophysiology of different leukemias and novel drugs. However, the five-year overall survival rate of some leukaemia patients, including AML, remains <30% and prognosis is grim for recurrent cases that have already undergone first-line induction therapy, with <10% surviving five years after relapse.^2^ Therefore, there is still an urgent need for new and effective treatment strategies for leukaemia, especially in relapsed and refractory cases.

Considering that several investigations have focussed on the leukaemia cell alone, a broader perspective taking into account the leukaemia cell microenvironment may be necessary to better appreciate leukaemia pathobiology.^3^ An increasing amount of evidence has revealed that leukaemia and endothelial cells depend on each other for survival and proliferation. Thus, targeting both leukaemia and endothelial cells with drugs such as anti-angiogenic agents, can lead to anti-proliferation and anti-angiogenesis effects, which may be a promising strategy.^4^^,^^5^

**Figure 1.**
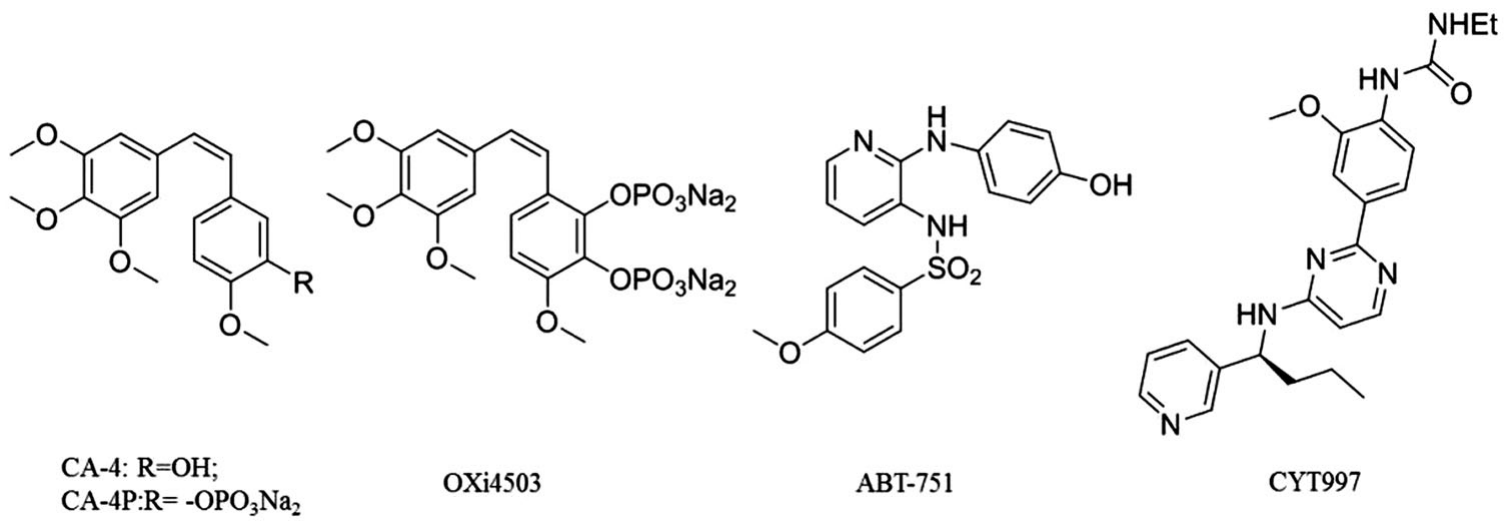
Representative anti-angiogenic agents binding to tubulin colchicine binding site.

Due to the growth, progression and metastasis of cancers relying on a functional network of blood vessels, study of the vasculature has become a promising field in the treatment of leukaemia and solid tumours.^6^^,^^7^ Anti-angiogenesis has been a vital strategy for the treatment of different tumours. Numerous angiogenesis related factors such as VEGFR-2, Tie-2, EphB4 and tubulin have been identified as potential targets for angiogenesis inhibitors.^8–10^ Tubulin, the necessary protein for the formation of the mitotic spindle and mitotic division of cell, has been an important target for the design and development of anti-cancer drugs.^11^^,^^12^ In the past ten years, a series of tubulin colchicine site inhibitors had been found to display strong vascular disrupting and anti-angiogenesis activity.^13^ Some of them have entered the clinical trials, including CA4P, OXi4503 and ABT-751 (Figure 1).

Combretastatin (CA4P), the first-in-class molecule against the tubulin colchicine binding site, showed significant anti-angiogenic effect and anti-leukaemia potency in vivo.^14^ Soon after that, another tubulin inhibitor, OXi4503, had been identified and shown to display more potent vascular disruption and antitumor activity than CA4P *in vitro* and in vivo. OXi4503 exhibited single-agent anti-leukaemia activity in murine xenograft models of acute myeloid leukaemia (AML) and in clinical phase for relapsed/refractory AML.^15^ The derivatives of CA-4, CA4P and OXi4503 have serious cardiovascular toxicities and easily undergo isomerisation via cis double bonds during storage and administration. Aside from CA-4 and its derivatives, there are also other inhibitors with diverse structural scaffolds that bind to the colchicine site as anti-vascular agents, for example, ABT-751. ABT-751 is an orally active antimitotic agent against a wide range of human tumour cell lines including leukaemia. In phase I trials for refractory haematologic malignancies, ABT-751 can be absorbed and eliminated rapidly.^16^ CYT997 is another tubulin colchicine binding agent with strong anti-angiogenic and anti-vascular potency. CYT997 may represent a promising approach for the treatment of AML. CYT997 induces cell death in CD123^+^ leukaemia cells and significantly reduces leukaemia colony formation when used alone or in combination with other agents.^17^ Although leukaemia vasculature is an important target for treatment, the number and diversity of tubulin colchicine binding site agents with strong anti-angiogenic potency are still limited.

**Figure 2.**
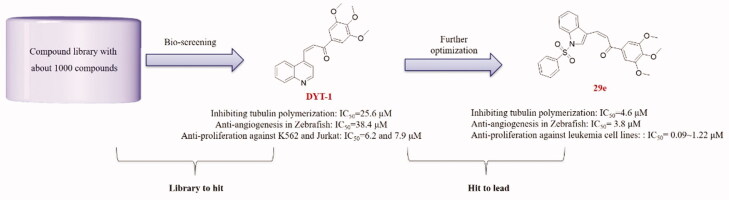
Identification of hit compound (**DYT-1**) from our chemical library and further optimizations to discover lead compound (**29e**).

To identify potent anti-angiogenic agents binding to the tubulin colchicine binding site against leukaemia with a novel scaffold, based on our previous work on the identification of tubulin colchicine binding site inhibitors, we screened our in-house structurally diverse molecular library (ca. 1000 compounds)^18–25^ and subsequently examined medicinal chemistry indications, leading to the identification of highly potent anti-angiogenic agents targeting the tubulin colchicine binding site against leukaemia (Figure 2).

## 2. Results and discussion

### 2.1. Chemistry

**Scheme 1.**
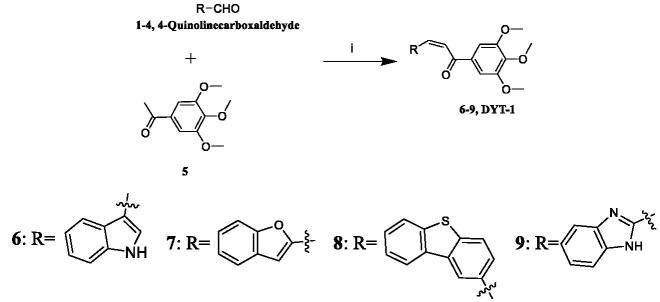
Reagents and conditions: (i) EtOH, 40% NaOH, 0 °C, 30 min; room temperature, 4 h.

**Scheme 2.**
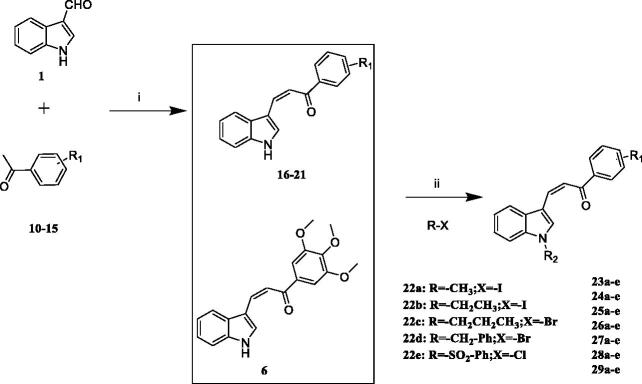
Reagents and conditions: (i) EtOH, 40% NaOH, 0 °C, 30 min; room temperature, 4 h; (ii) NaH, different haloalkanes, THF, 0 °C to room temperature.

The one-step synthetic pathway adopted to prepare **6**–**9** is depicted in Scheme 1. Different aldehydes reacted with 3′,4′,5′-trimethoxyacetophenone under the presence of NaOH solution resulting in excellent yield of compound **6**–**9** (synthetic detail procedure is given in the Experimental section). As shown in Scheme 2, the first step to prepare compounds **16–21** was similar to step i above mentioned. Then the intermediates **6, 16–21** and different haloalkanes reacted in the presence of NaH to yield the target compound **23a-29e**. **DYT-1** was prepared similarly to **6–9** and **16–21**. 4-Quinolinecarboxaldehyde reacted with 3′,4′,5′-trimethoxyacetophenone led to **DYT-1**. Also, we should point out that compounds **6**^22^**, 23-27a,**^26^ **28a**^27^ and **29a-d**^22^ ever were reported in our previous research and others. However, these reported compounds never were evaluated for anti-angiogenic activity.

### 2.2. Biological evaluation

#### 2.2.1. Sar of chalcone derivatives based on the tubulin inhibitory assay in vitro and Transgenic-Zebrafish-Based assays

For SAR analysis, different strategies have been used including tubulin- and zebrafish-based assays both *in vitro* and in vivo. An increasing number of studies have shown that transgenic zebrafish (FLK-1:EGFP) assays were a less costly and more rapid method in discovering antiangiogenic activity. Thereby, the combination of tubulin- and transgenic-zebrafish-based assays is an effective method for identifying agents with antimitotic and anti-angiogenic potency.

First, the left-hand side (LHS) quinoline moiety based on the isostere strategy leading to the formation of compounds **6**–**9** with indole, benzofuran, dibenzothiophene and benzimidazole, respectively, was examined. As shown in Table 1, the tubulin inhibitory polymerisation (IC_50_) of **6**–**9** was 8.5, 18.6, 45.7 and 20.8 µM, respectively. The antiangiogenic activity in zebrafish assays also presented similar trend. The compounds **6**–**7** with better tubulin inhibitory polymerisation activity were more potent than **8**–**9** on antiangiogenic assays. Considering the results of tubulin inhibition *in vitro* and anti-angiogenesis in vivo, **6** was selected to further investigate the SAR.

**Table 1.** Tubulin inhibitory activities and antiangiogenic activities in Zebrafish of chalcone derivatives with variation of the LHS

Cpd.	R	Tubulin^a^(μM)	Zebrafish^a^(μM)
**6**	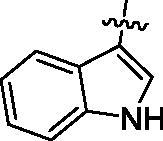	8.5 ± 1.2	27.8 ± 0.9
**7**	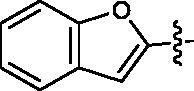	18.6 ± 0.5	29.5 ± 1.5
**8**	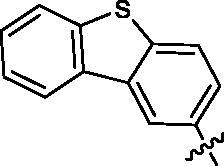	45.7 ± 2.3	68.6 ± 2.1
**9**	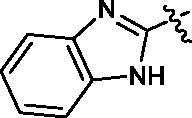	20.8 ± 1.4	>100
**CA-4**		9.0 ± 1.7	ND^b^

^a^Each compound was tested in triplicate; the data are presented as the mean ± SD.

^b^ND: not detected because of its strong toxicity.

Then we employed compound **6** as a template to explore the effects of different substituents on the LHS and right-hand side (RHS). As shown in Table 2, a variety of substituents at Ph and indole-N gave compounds **23a-29e.** As a whole, electron-donating groups including 3-Cl and 4-Cl on Ph were not beneficial for tubulin inhibitory activity retaining. By contrast, electron-withdrawing substituents were tolerated, such as -OCH_3_ and -CF_3_. Comparing the inhibitory potency of **26e** with **26a-26d**, it can be seen that the size accumulation on indole-N could increase potency, as did **29a-29e**. Interestingly, compound **28e** with moderate tubulin inhibitory potency but excellent anti-angiogenic activity indicated that the anti-angiogenic ability was not, at least partly, due to tubulin.

**Table 2.** Tubulin inhibitory activities and antiangiogenic activities in Zebrafish of chalcone derivatives with variation of both LHS and RHS.

Cpd.	R^1^	R^2^	Tubulin^a^(μM)	Zebrafish^a^(μM)
**23a**	H	-CH_3_	>100	–^b^
**23b**	H	-CH_2_CH_3_	>100	–
**23c**	H	-CH_2_CH_2_CH_3_	>100	–
**23d**	H	-CH_2_-Ph	68.6 ± 3.2	–
**23e**	H	-SO_2_-Ph	>100	–
**24a**	−3-Cl	-CH_3_	>100	–
**24b**	−3-Cl	-CH_2_CH_3_	>100	–
**24c**	−3-Cl	-CH_2_CH_2_CH_3_	>100	–
**24d**	−3-Cl	-CH_2_-Ph	>100	–
**24e**	−3-Cl	-SO_2_-Ph	92.3 ± 2.4	–
**25a**	−4-Cl	-CH_3_	>100	–
**25b**	−4-Cl	-CH_2_CH_3_	>100	–
**25c**	−4-Cl	-CH_2_CH_2_CH_3_	>100	–
**25d**	−4-Cl	-CH_2_-Ph	>100	–
**25e**	−4-Cl	-SO_2_-Ph	>100	–
**26a**	−4-OCH_3_	-CH_3_	85.7 ± 2.5	–
**26b**	−4-OCH_3_	-CH_2_CH_3_	78.8 ± 1.3	–
**26c**	−4-OCH_3_	-CH_2_CH_2_CH_3_	72.5 ± 1.7	–
**26d**	−4-OCH_3_	-CH_2_-Ph	75.8 ± 2.6	–
**26e**	−4-OCH_3_	-SO_2_-Ph	58.4 ± 1.7	35.6 ± 3.2
**27a**	−3-OCH_3_	-CH_3_	>100	–
**27b**	−3-OCH_3_	-CH_2_CH_3_	>100	–
**27c**	−3-OCH_3_	-CH_2_CH_2_CH_3_	52.1 ± 2.9	18.6 ± 3.2
**27d**	−3-OCH_3_	-CH_2_-Ph	>100	–
**27e**	−3-OCH_3_	-SO_2_-Ph	87.2 ± 1.4	–
**28a**	−4-CF_3_	-CH_3_	>100	–
**28b**	−4-CF_3_	-CH_2_CH_3_	>100	–
**28c**	−4-CF_3_	-CH_2_CH_2_CH_3_	26.8 ± 4.6	
**28d**	−4-CF_3_	-CH_2_-Ph	>100	–
**28e**	−4-CF_3_	-SO_2_-Ph	10.4 ± 0.7	5.8 ± 2.1
**29a**	−3,4,5-OCH_3_	-CH_3_	49.9 ± 8.5	15.7 ± 3.2
**29b**	−3,4,5-OCH_3_	-CH_2_CH_3_	>100	–
**29c**	−3,4,5-OCH_3_	-CH_2_CH_2_CH_3_	>100	–
**29d**	−3,4,5-OCH_3_	-CH_2_-Ph	24.8 ± 2.1	6.7 ± 0.5
**29e**	−3,4,5-OCH_3_	-SO_2_-Ph	4.8 ± 0.7	3.4 ± 0.3

^a^Each compound was tested in triplicate; the data are presented as the mean ± SD.

^b^Not tested.

In a summary, efforts of structural optimisation and SAR studies led to the discovery of **29e**, which exhibited both high potency against tubulin (IC_50_=4.8 µM) and a considerable antiangiogenic effect in transgenic-zebrafish (IC_50_=3.4 µM). Further in-depth study *in vitro* and in vivo were subsequently performed with compound **29e**.

#### 2.2.2. Leukaemia cell lines growth inhibitory activity of compound 29e

Four myeloid leukaemia cell lines (MV4-11, HL60, K562, THP-1) and three lymphoid leukaemia cell lines (CCRF-CEM, Jurkat, HuT 78) had been selected to evaluate the potency of **29e** by CCK-8 assay (Table 3). Overall, compound **29e** had more potent inhibitory activity on myeloid leukaemia cell lines than lymphoid leukaemia. Notably, compound **29e** showed the best growth inhibitory activity against K562 with an IC_50_ of 0.09 µM.

**Table 3.** IC_50_ Values of **29e** and CA-4 against various leukaemia cell lines

Tumour type	Cell line	IC_50_^a^(μM)	
		29**e**	CA-4
leukaemia, AML	MV4-11	0.25 ± 0.02	0.12 ± 0.03
leukaemia, APL	HL60	0.18 ± 0.002	0.26 ± 0.05
leukaemia, CML	K562	0.09 ± 0.001	0.27 ± 0.01
leukaemia, AML	THP-1	0.37 ± 0.03	0.08 ± 0.01
leukaemia, ALL	CCRF-CEM	0.84 ± 0.08	0.71 ± 0.11
leukaemia, ALL	Jurkat	1.22 ± 0.12	0.19 ± 0.02
leukaemia, ALL	HuT 78	0.26 ± 0.11	0.24 ± 0.04

^a^Each compound was tested in triplicate; the data are presented as the mean ± SD.

#### 2.2.3. Compound 29e induced K562 apoptosis *in vitro*

**Figure 3.**
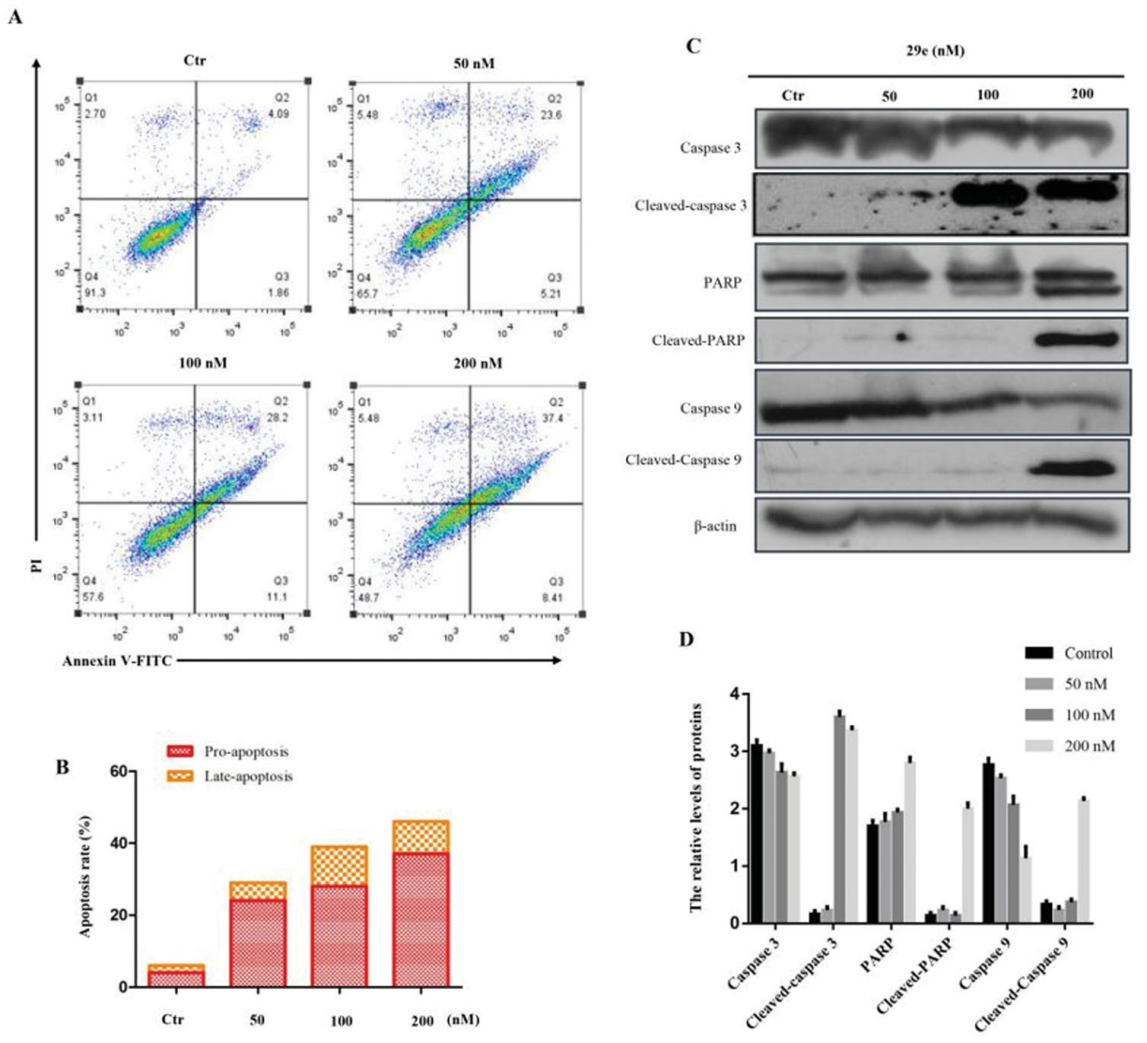
Compound **29e** induced apoptosis K562 cells. (A) Apoptosis ratio detection by flow cytometry assays for 48 h. (B) The quantitative analysis of apoptotic rate at early and advanced stages of K562 cells. (C) Western blot analysis of the apoptosis related proteins. (D) The quantitative analysis of the protein levels. The data was presented as the mean ± SD of three independent tests.

To test whether apoptosis was related to the cell growth inhibition, the compound **29e** -treated K562 cell line was analysed by flow cytometry. As displayed in Figure 3(A,B), **29e** induced cell apoptosis in a concentration-dependent manner. The percentage of apoptotic cell significantly increased from 5.95% to 45.81% after treatment of **29e**. In contrast to the control, the percentage of pro-apoptosis treated with **29e** increased more obviously than late-apoptosis. To further confirm this result, we employed a western blot assay to detect the expression of apoptosis-related proteins. Caspases, a family of cysteine-aspartic proteases, are the central executioners of apoptosis. As a critical step in the process of apoptotic cellular death, caspase activation is mediated by various inducers. Caspase-3 is responsible for chromatin condensation and DNA fragmentation, which represents the hallmark of apoptosis. The cleavage of poly (ADP-ribose) polymerase (PARP) is an important indicator of apoptosis and generally considered to be an indicator of caspase-3 activation. As shown in Figure 3(C,D), the expression of caspase-3, caspase-9 and PARP (apoptosis-promoting protein) decreased efficiently. Inversely, treatment of K562 cell lines with compound **29e** resulted in an up-regulation of cleaved caspase-3 and PARP, which may be closely related to K562 apoptosis.

#### 2.2.4. Compound 29e bound to tubulin colchicine site and inhibited tubulin polymerisation

**Figure 4.**
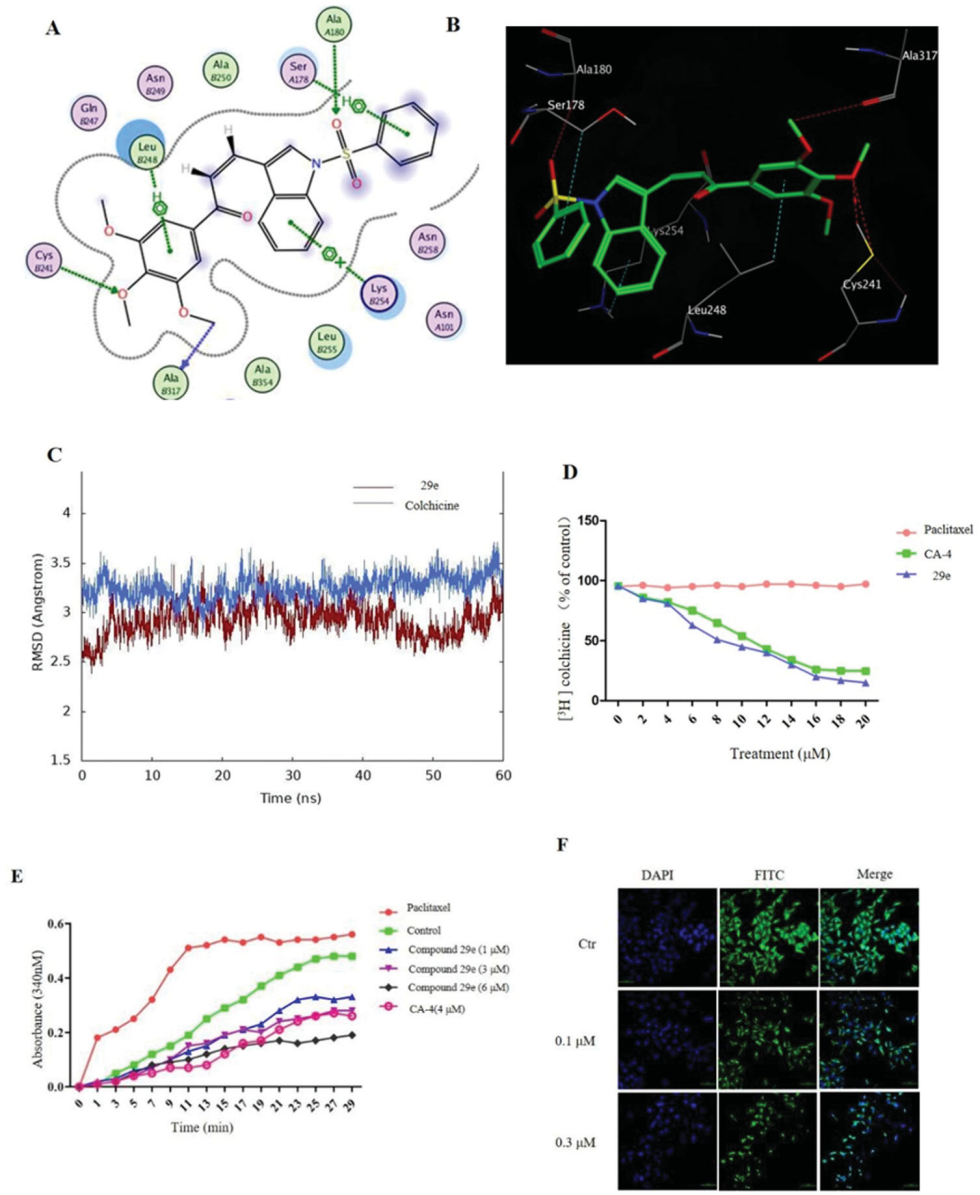
Compound **29e** bind to the colchicine site of tubulin and inhibit the microtubule polymerisation. (A) 2 D model of the interaction between compound **29e** and the amino acid residues of tubulin colchicine site. (B) 3 D model of the binding position of compound **29e**. (C) RMSD tendency of two systems (**29e**, colchicine) versus time in the 60 ns MD simulation. (D) Effect of compound **29e** on tubulin binding of [^3^H] colchicine. (E) Compound **29e** affected microtubule assembly *in vitro*. (F) The effects of **29e** on the organisation of cellular microtubule network of K562 cells.

To elucidate whether compound **29e** targeted the microtubule system and evaluate how it bind, we carried out a molecular docking study to predict the possible binding mode of **29e** with tubulin (PDB code: 1SA0) using the software Moe 2015. The docking result (Figure 4(A,B)) showed that **29e** had a similar binding pose compared to colchicine where the 3,4,5,-trimethoxyphenyl ring was placed in proximity to Cys 241 by use of a hydrogen bond.^22^ Furthermore, the 3,4,5,-trimethoxyphenyl ring formed a π-H bond and hydrogen bond with Leu 248 (distance = 3.67 Å) and Ala 317 (distance = 3.47 Å), respectively. The only π -cation bond was formed from the interaction of indole ring and Lys 254, which had a distance of 4.16 Å. As expected, the benzenesulfonyl moiety played an important role, demonstrated by its interaction with Ala 180 and Ser 178. Based on the binding pose from the docking result, molecular dynamics (MD) simulations were then carried out in explicit aqueous solution for 10 ns consisting of colchicine and compound **29e**. The stabilities under simulation were evaluated by means of the root-mean-square deviation (RMSD). The results described in Figure 4(C) suggested that both systems were stable during the 60-ns MD simulation. The RMSD values of compound **29e** fluctuated from 3 to 3.5 Å after reaching the summit, which demonstrated that the hit compound **29e** was stabilised in the tubulin colchicine binding site. To confirm if the compound **29e** bound to the tubulin colchicine binding site, we performed a radioligand binding assay by competing with the [^3^H]colchicine binding to tubulin (Figure 4(D)). As shown in Figure 4(D), 29e efficiently bound to the [^3^H]colchicine binding domain of tubulin compared with the positive control CA-4, which demonstrated that **29e** directly interacted with tubulin by the pocket of colchicine binding site. To further evaluate the tubulin polymerisation inhibition ability of **29e,** *in vitro* tubulin was incubated with 1uM, 3uM and 6uM compound **29e** (Figure 4(E)). The IC_50_ of inhibiting tubulin polymerisation was 4.6uM, which revealed **29e** was a novel inhibiting tubulin polymerisation agent. The immunofluorescence staining assay on K562 cell line was performed to evaluate the effects of compound **29e** against the microtubule cytoskeleton (Figure 4(F)). For the control group, the microtubule network of K562 cells displayed a normal arrangement and tissue morphology. In contrast, the result from the groups treated with 0.1 uM and 0.3 uM compound **29e** showed conspicuous disorder for the microtubule network; the microtubule cytoskeleton was destroyed and shrunk to the cell border. These results supported the theory that compound **29e** was a potent tubulin polymerisation inhibitor *in vitro*.

#### 2.2.5. Anti-angiogenesis effect of compound 29e *in vitro*

**Figure 5.**
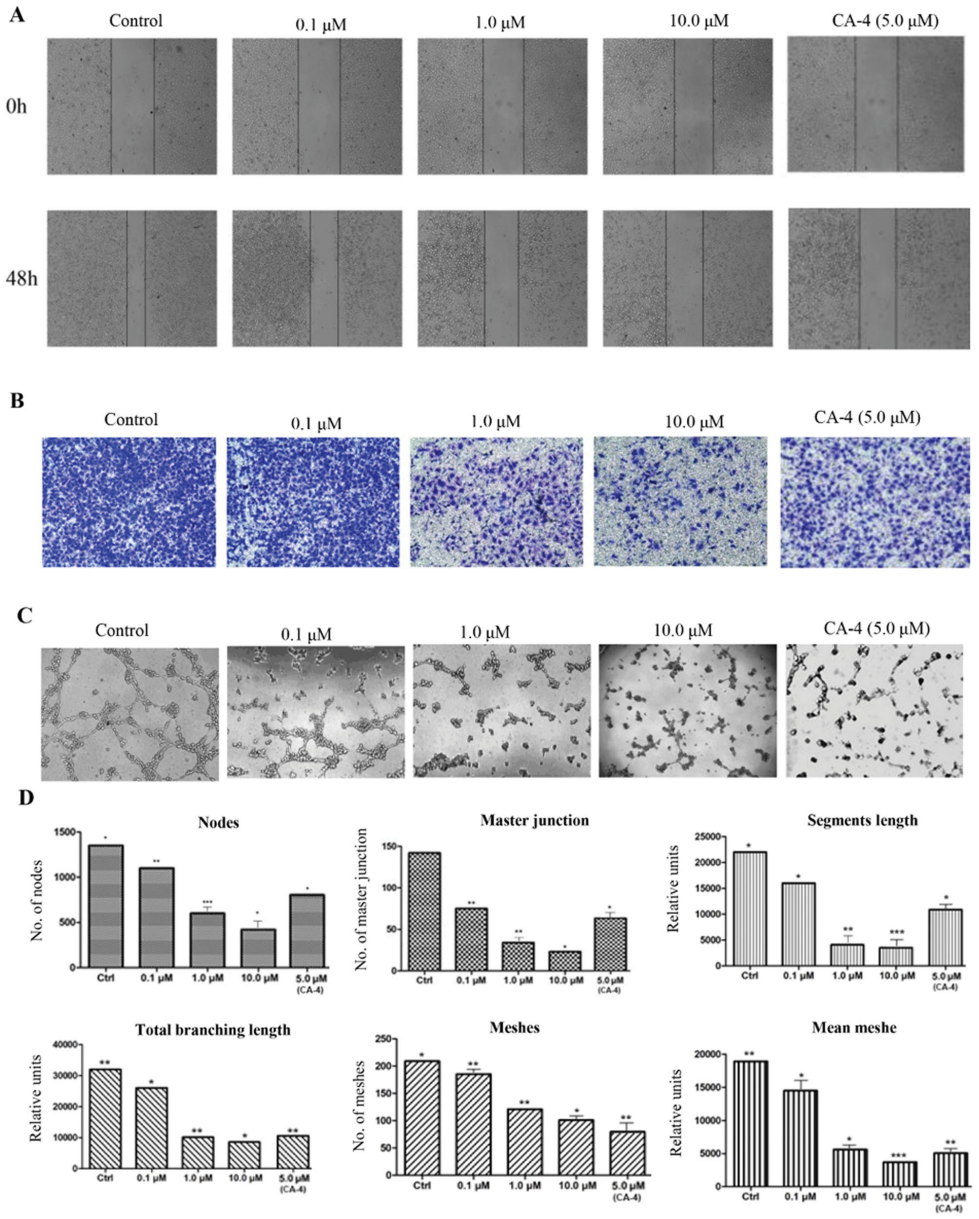
Compound **29e** showed antivascular activity *in vitro*. (A) The wound-healing assay was used to evaluate the migration of HUVEC cells, and images were captured at 0 h and 48 h after treatments with **29e**. (B) The invasion suppressing effects of **29e** against HUVECs cells by Transwell assay. (C) Typical images depicting tubule formation of HUVEC cells by treatments with **29e** for 6 h. (D) Quantitative analysis of the migration ability of HUVEC tubule formation.

Human umbilical vein endothelial cells (HUVECs) seeded on matrigel is a canonical tool to confirm anti-angiogenesis effects.^28^^,^^29^ As shown in Figure 5(A), we adopted Transwell to explore the inhibitory potency of compound **29e** on HUVEC invasion. **29e** did not cause any toxicity on HUVEC at the concentration of 10 µM with IC_50_ value of 58.6 µM. Also, considering the toxicity of **29e** on the leukaemia cell lines, we selected the concentration of 0.1 µM, 1.0 µM and 10 µM to perform the anti-angiogenesis effect. As the concentration of **29e** increased from 0.1 µM to 10 µM, the number of HUVEC invasion decreased, which demonstrated that the invasion inhibitory effect relied on the concentration-dependent manner. The result of the wound healing assay (Figure 5(B)) further confirmed that **29e** had the capacity to prevent HUVEC motility and migration. Exposed to different concentrations of compound **29e** at 0.1, 1 and 10µΜ, **29e** markedly decreased the closure of wound scratching in a confluent monolayer of HUVEC. To evaluate anti-vascular activity of compound **29e**, we examined 0.1, 1 and 10µΜ **29e** on HUVEC by a 1 h treatment (Figure 5(C,D)). Compared to the control, **29e** sharply reduced the width and length of “tubule-like” networks. The parameters from Figure 5(D) (segment length, the area and the number of meshes, the percent of area, the number of branching points) obtained by the standard image analysis suggested the promising potential of the anti-angiogenesis activity of **29e**.

#### 2.2.6. Antiangiogenic activity of compound 29e in vivo

**Figure 6.**
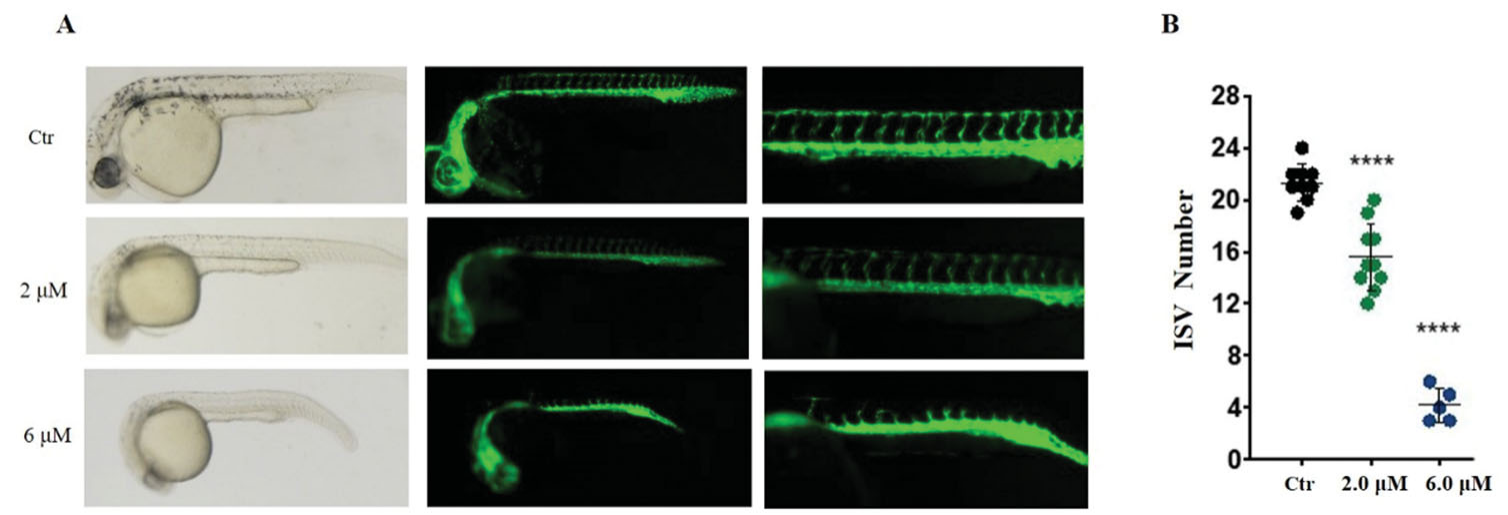
Anti-angiogenic effect of **29e** evaluated by zebrafish embryos assay. (A)Inhibitory effect of **29e** on the angiogenesis of transgenic zebrafish. (B) Histogram showed the numbers of zebrafish ISVs per field under confocal microscopy.

The antiangiogenic effect of compound **29e** was evaluated in zebrafish embryos, in which vascular endothelial cells were labelled with green fluorescent protein (GFP).^30^ Figure 6 displays 3hpf zebrafish embryos with the treatment of different concentrations of 2.0, and 6.0 µM **29e**, as well as the control. 2.0 µM **29e** efficiently blocked the formation of intersegmental vessels (ISVs). At a concentration of 6.0 µM, the number and length of ISVs completely decreased, indicating a dose-dependent inhibition pattern.

#### 2.2.7. *In vivo* effects of compound 29e in a zebrafish xenograft

**Figure 7.**
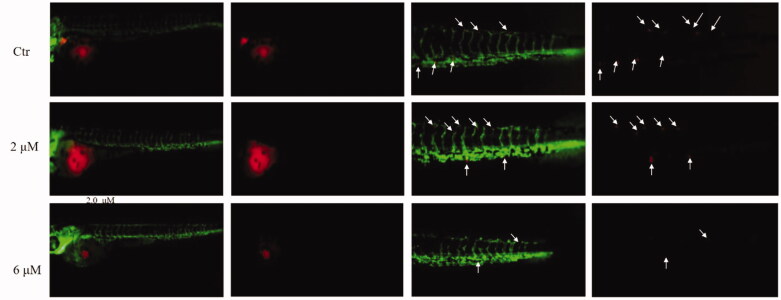
Inhibitory effects of **29e** on the proliferation and metastasis of K562 cells in zebrafish xenograft models. CMTPX labelled K562 cells (red) were microinjected into zebrafish embryos, and indicated concentration of **29e** were added. White solid arrows indicate disseminated cells.

Different publications had suggested that xenotransplantation of human tumour cells in zebrafish has served as a major model utilised by pharmacologists for several decades with the advantages of low-cost, high reproductive ability, superior imaging qualities and little immunorejection.^31^^,^^32^ The in vivo anti-leukaemia potency of compound **29e** was evaluated using the K562 cell xenograft in zebrafish embryos. Using a transgenic zebrafish (fil1:EGFP), a K562-bearing model was established, in which the blood vessels were labelled green fluorescence.The red fluorescently labelled K562 cells were serially transplanted in limiting dilutions to identify the leukaemia cells near the SIV of the zebrafish. A preliminary cytotoxicity study on embryos had performed, **29e** did not cause the toxicity at the concentration of 6µΜ with IC_50_ value of 44.6 µM. As shown in Figure 7, a spot of red K562 cells in the control group disseminated and widely migrated away from the primary area, whereas treatments with **2 **µM and 6 µM **29e** showed reduced intensities and decreased tumour area compared with the control group. These results indicated that **29e** can suppress the proliferation and metastasis of K562 cells in zebrafish xenografts in a dose-dependent manner, subsequently indicating that **29e** can be developed as a potential candidate against leukaemia.

#### 2.2.8. Physicochemical property and metabolic stability of compound 29e

To evaluate the drug-likeness of compound **29e**, various physicochemical and metabolic parameters had been tested. As shown in Table 4, **29e** was with a polar surface area (PSA) of 83.85 placed between 80 to 90 Å, well within the range considered appropriate for passive absorption. Distribution coefficients displayed an excellent drug-like cLogD value which was 3.7. Aqueous solubility ranged from a relatively low 25–50 µg/mL at pH 2.0 for **29e** to a more acceptable value of up to 150 µg/mL at pH 6.5. On the whole, the lead compound **29e** possessed favourable solubility and permeability properties. Metabolic liabilities of compound **29e** were also assessed with mouse (MLM) and human (HLM) liver microsomes, respectively. The *in vitro* degradation half-life in HLM was lower than in MLM, and there was also a significant difference for intrinsic clearance (CLint) between them.

**Table 4.** Physicochemical parameters and metabolic stability of compound **29e**

MW^a^	PSA^a^	cLogD^a^	Solubility^b^(μg/mL)		Degradation half-life (min)		In Vitro Clint (µL/min/mg protein)	
			pH 2.0	pH 6.5	Human	Mouse	Human	Mouse
477.5	83.85	3.7	25–50	75–150	35	60	16	77

^a^Calculated using ChemAxon JChem software; ^b^Kinetic solubility determined by nephelometry (SolpH).

## 3. Conclusion

Potent anti-angiogenic agents binding to the tubulin colchicine binding site are effective for leukaemia therapy in clinic. Although the huge number of tubulin inhibitors had been reported in the past decades, the research about anti-angiogenic activity especially with strong leukaemia cell lines inhibition activity is very limited. Here, we used DYT-1 from our in-house library with a novel indole-chalcone scaffold as a lead, and explored the SAR of its derivatives based on cell and zebrafish assays, which resulted in the discovery of compound **29e** with considerably antiproliferative potency against a variety of leukaemia cell lines. Compound **29e** showed the most potent tumour cell growth inhibitory activity towards K562 and induced its apoptosis with the expression change of apoptosis-related protein including caspase-3, caspase-9 and PARP. Further molecular docking, molecular dynamics (MD) simulation, radioligand binding assay and cellular microtubule networks disrupting result of compound **29e** were in agreement with its ability of inhibiting tubulin polymerisation and predicted binding mode. By tube formation assay, Transwell, and wound healing analysis, compound **29e** could inhibit HUVEC tube formation, invasion and migration. The inhibitory effect of compound **29e** on angiogenesis in vivo was verified in blood vessel-specific fluorescent transgenic zebrafish. **29e** also prominently reduced K562 cell proliferation and metastasis in blood vessels and surrounding tissues in zebrafish xenograft model. Further in-depth assays were performed with compound **29e,** which possessed good physicochemical property and metabolic stability. Based on the above findings, compound **29e** could be considered as a potential anti-leukaemia drug candidate for further development.

## 4. Experimental part

### 4.1. Chemistry

#### 4.1.1. General methods

Unless otherwise stated, all chemical reagents and solvents were purchased from commercial sources and could be used without further purification and melting points were measured on a capillary electrothermal melting point apparatus without calibration. Column chromatography (CC) was conducted on silica gel (200–300 mesh, Qingdao Ocean Chemical Company, China). Thin-layer chromatography (TLC) analyses were conducted on silica gel GF254 (Qingdao Ocean Chemical Company, China) glass plates. ^1^HNMR was recorded on a Bruker AV-400 nuclear magnetic resonance spectrometer as deuterated chloroform (CDCl_3_) or deuterated dimethyl sulfoxide (DMSO-d6) solutions. Chemical shifts were reported in parts per million (ppm) relative to tetramethyl silanes as an internal standard.

#### 4.1.2. General procedure for synthesis of compound DYT-1, 6–9

Different aldehydes and 3′,4′,5′-trimethoxyacetophenone (10 mmol) in ethanol (25 ml) were mixed gently at 0 °C. Then 40% NaOH (5 ml) was added and stirred for 30 min. The mixture was placed to room temperature to continue the reaction for 4 h and the solid was filtered, washed with water and dried to obtain compound ***DYT-1*** *and* **6–9.**^33^^,^^34^

#### 4.1.3. General procedure for synthesis of compound 23a-29e

Compounds **23a**-**29e** were prepared similarly to step i above mentioned. Then NaH (60% dispersion in mineral oil, 2.0 mmol) was added in portions to a stirred solution of intermediate compounds (**5,10–15**) in anhydrous THF (10.0 ml) cooled in an ice bath. The resulting mixture was then allowed slowly to warm to r.t. After stirring for 30 min, different haloalkanes (3.0 mmol) in anhydrous THF (3.0 ml) was added drop wise.^35^ When TLC monitoring showed complete consumption of the starting material, the reaction mixture was evaporated under reduced pressure to leave a residue that was treated with ice water (100 ml). The resulting solid was filtrated off and recrystallized from acetone/petroleum ether (60–90 °C) to give the target compounds. The purity of all these synthesised compounds were above 95%.

##### 4.1.3.1. (*E*)-3-(quinolin-4-yl)-1–(3,4,5-trimethoxyphenyl)prop-2-en-1-one (DYT-1)

^1^H NMR (400 MHz, Chloroform-*d*) δ 8.99 (d, *J* = 4.5 Hz, 1H), 8.54 (d, *J* = 15.5 Hz, 1H), 8.24–8.16 (m, 2H), 7.80 (ddd, *J* = 8.4, 6.9, 1.4 Hz, 1H), 7.71–7.63 (m, 3H), 7.33 (s, 2H), 3.97 (d, *J* = 1.6 Hz, 9H). ^13 ^C NMR (101 MHz, Chloroform-*d*) δ 188.28, 153.29, 150.09, 148.72, 140.69, 138.85, 132.72, 130.27, 129.85, 127.78, 127.39, 126.29, 123.44, 118.15, 106.33, 61.05, 56.47, 56.44. ESI-MS Calcd for [M + H]^+^ C_21_H_20_NO_4_: 350.1392, found: 350.1387.

##### 4.1.3.2. (*E*)-3–(1-Methyl-1H-indol-3-yl)-1-phenylprop-2-en-1-one (23a)

Yellow solid, yield 87%, m.p. 160–162 °C; ^1^H NMR (DMSO-d_6_, 300 MHz) *δ*: 8.21–8.02 (m, 5H, ArH), 7.68–7.56 (m, 5H, ArH, CH), 7.34–7.26 (m, 2H, CH), 3.87 (S, 3H, CH_3_), ^13 ^C NMR (101 MHz, Chloroform-*d*) δ 190.65, 139.16, 138.62, 138.26, 134.56, 132.08, 128.46, 128.25, 126.13, 123.14, 121.54, 120.74, 116.99, 112.96, 110.13, 33.22. ESI-MS Calcd for [M + H]^+^ C_18_H_16_NO: 262.1232, found: 262.1223.

##### 4.1.3.3. (*E*)-3–(1-Ethyl-1H-indol-3-yl)-1-phenylprop-2-en-1-one (23 b)

Yellow solid, yield 73%, m.p. 165–168 °C; ^1^H NMR (DMSO-d_6_, 300 MHz) *δ*: 8.13–8.02 (m, 5H, ArH), 7.67–7.57 (m, 5H, ArH, CH), 7.36–7.27 (m, 2H, CH), 4.31–4.26 (m, 2H, CH_2_), 1.453 (t, 3H, *J* = 4.502 Hz, CH_3_), ^13 ^C NMR (101 MHz, Chloroform-*d*) δ 189.68, 138.18, 137.71, 136.34, 131.80, 131.03, 127.44, 127.24, 125.34, 122.01, 120.50, 119.85, 115.97, 112.07, 109.18, 40.42, 14.14. ESI-MS: m/z 275.13 (M^+^). Anal. Calcd for C_19_H_17_NO: C, 82.88; H, 6.22; N, 5.09. ESI-MS Calcd for [M + H]^+^ C_19_H_18_NO: 275.1388, found: 275.1378.

##### 4.1.3.4. (*E*)-1-Phenyl-3–(1-propyl-1H-indol-3-yl)prop-2-en-1-one (23c)

Light yellow solid, yield 83%, m.p. 161–162 °C; ^1^H NMR (DMSO-d_6_, 300 MHz) *δ*: 8.13–8.02 (m, 5H, ArH), 7.67–7.57 (m, 5H, ArH, CH), 7.36–7.27 (m, 2H, CH), 4.31–4.26 (m, 2H, CH_2_), 1.44 (t, 3H, *J* = 4.502 Hz, CH_3_), ^13 ^C NMR (101 MHz, Chloroform-*d*) δ 190.72, 139.18, 138.79, 137.62, 133.74, 132.07, 128.46, 128.26, 126.28, 123.01, 121.48, 120.87, 116.97, 112.91, 110.35, 48.48, 23.22, 11.43. ESI-MS Calcd for [M + H]^+^ C_19_H_20_NO: 290.1545, found: 290.1535.

##### 4.1.3.5. (*E*)-3–(1-Benzyl-1H-indol-3-yl)-1-phenylprop-2-en-1-one (23d)

Red solid, yield 87%, m.p. 161–163 °C; ^1^H NMR (DMSO-d_6_, 300 MHz) *δ*: 8.36 (s, 1H, ArH), 8.15–8.03 (m, 4H, ArH), 7.36–7.26 (m, 5H, ArH), 7.36–7.26 (m, 7H, ArH, CH), 5.50 (S, 2H, CH_2_), ^13 ^C NMR (101 MHz, Chloroform-*d*) δ 188.88, 162.92, 137.72, 137.61, 136.07, 133.47, 131.81, 130.47, 128.92, 128.02, 126.92, 123.19, 121.56, 120.79, 117.18, 113.68, 113.51, 110.62, 55.42, 50.42. ESI-MS Calcd for [M + H]^+^ C_24_H_20_NO: 338.1545, found: 338.1534.

##### 4.1.3.6. (*E*)-1-Phenyl-3–(1-(phenylsulfonyl)-1H-indol-3-yl)prop-2-en-1-one (23e)

Yellow solid, yield 73%, m.p. 165–168 °C; ^1^H NMR (DMSO-d_6_, 300 MHz) *δ*: 8.71 (s, 1H, ArH), 8.20–8.12 (m, 3H, ArH), 8.02–7.94 (m, 5H, ArH), 7.71–7.58 (m, 3H, ArH, CH), 7.48–7.39 (m, 5H, ArH), ^13 ^C NMR (101 MHz, Chloroform-*d*) δ 190.19, 138.14, 137.69, 135.68, 135.62, 134.34, 132.84, 129.54, 128.90, 128.70, 128.47, 128.30, 126.96, 125.70, 124.30, 122.21, 120.78, 119.00, 113.90. ESI-MS Calcd for [M + H]^+^ C_24_H_18_NO_3_S: 388.1007, found: 338.1000.

##### 4.1.3.7. (*E*)-1–(3-Chlorophenyl)-3–(1-methyl-1H-indol-3-yl)prop-2-en-1-one (24a)

Yellow solid, yield 91%, m.p. 173–175 °C; ^1^H NMR (DMSO-d_6_, 300 MHz) *δ*: 8.22–8.03 (m, 5H, ArH), 7.85–7.81 (m, 1H, ArH), 7.64–7.52 (m, 3H, ArH,), 7.35–7.25 (m, 2H, CH), 3.88 (S, 3H, CH_3_), ^13 ^C NMR (101 MHz, Chloroform-*d*) δ 189.14, 140.86, 139.45, 138.32, 134.93, 134.73, 131.97, 129.79, 128.35, 126.32, 126.13, 123.30, 121.75, 120.76, 116.27, 112.95, 110.22, 33.32. ESI-MS Calcd for [M + H]^+^ C_18_H_15_ClNO: 296.0842, found: 296.0832.

##### 4.1.3.8. (*E*)-1–(3-Chlorophenyl)-3–(1-ethyl-1H-indol-3-yl)prop-2-en-1-one (24 b)

Yellow solid, yield 73%, m.p. 165–168 °C; ^1^H NMR (DMSO-d_6_, 300 MHz) *δ*: 8.21–8.04 (m, 5H, ArH), 7.85–7.83 (m, 2H, ArH), 7.65–7.53 (m, 3H, ArH), 7.35–7.25 (m, 2H, CH), 4.31–4.26 (m, 2H, CH_2_), 1.43 (t, 3H, *J* = 4.51 Hz, CH_3_), ^13 ^C NMR (101 MHz, Chloroform-*d*) δ 189.21, 140.91, 139.57, 137.42, 134.73, 133.19, 131.94, 129.78, 128.36, 126.36, 126.33, 123.19, 121.72, 120.88, 116.25, 113.06, 110.29, 41.53, 15.17. ESI-MS: m/z 309.09 (M^+^). Anal. Calcd for C_19_H_16_ClNO: C,73.66; H, 5.22; N, 4.53. ESI-MS Calcd for [M + H]^+^ C_19_H_17_ClNO: 310.0999, found: 310.0990.

##### 4.1.3.9. (*E*)-1–(3-Chlorophenyl)-3–(1-propyl-1H-indol-3-yl)prop-2-en-1-one (24c)

Light yellow solid, yield 83%, m.p. 161–162 °C; ^1^H NMR (DMSO-d_6_, 300 MHz) *δ*: 8.13–8.02 (m, 5H, ArH), 7.85–7.83 (m, 2H, ArH), 7.65–7.53 (m, 3H, ArH,), 7.33–7.27 (m, 2H, CH), 4.23–4.20 (m, 2H, CH_2_), 1.87–1.81 (m, 2H, CH_2_), ^13 ^C NMR (101 MHz, Chloroform-*d*) δ 189.19, 140.86, 139.60, 137.65, 134.70, 134.10, 131.94, 129.77, 128.34, 126.31, 126.26, 123.14, 121.66, 120.84, 116.19, 112.85, 110.42, 48.53, 23.21, 11.42. ESI-MS Calcd for [M + H]^+^ C_20_H_19_ClNO: 324.1155, found: 324.1158.

##### 4.1.3.10. (*E*)-3–(1-Benzyl-1H-indol-3-yl)-1–(3-chlorophenyl)prop-2-en-1-one (24d)

red solid, yield 87%, m.p. 161–163 °C; ^1^H NMR (DMSO-d_6_, 300 MHz) *δ*: 8.38 (s, 1H, ArH), 8.23 (s, 1H, ArH), 8.17–8.06 (m, 3H, ArH), 7.86–7.84 (m, 1H, ArH), 7.68–7.53 (m, 3H, ArH, CH), 7.38–7.27 (m, 7H, ArH, CH) 5.52 (S, 2H, CH_2_), ^13 ^C NMR (101 MHz, Chloroform-*d*) δ 189.14, 140.71, 139.34, 137.80, 135.86, 134.69, 134.12, 131.98, 129.77, 128.97, 128.32, 128.13, 126.97, 126.34, 126.31, 123.41, 121.86, 120.81, 116.68, 113.39, 110.71, 50.53. ESI-MS Calcd for [M + H]^+^ C_24_H_19_ClNO: 372.1255, found: 372.1144.

##### 4.1.3.11. (*E*)-1–(3-Chlorophenyl)-3–(1-(phenylsulfonyl)-1H-indol-3-yl)prop-2-en-1-one (24e)

Yellow solid, yield 73%, m.p. 165–168 °C; ^1^H NMR (DMSO-d_6_, 300 MHz) *δ*: 8.75 (s, 1H, ArH), 8.32 (s, 1H, ArH), 8.23–8.13 (m, 2H, ArH), 8.01–7.86 (m, 6H, ArH), 7.58–7.54 (m, 1H, CH), 7.49–7.39 (m, 4H, ArH, CH), ^13 ^C NMR (101 MHz, Chloroform-*d*) δ 188.79, 139.75, 137.65, 136.47, 135.66, 134.98, 134.39, 132.72, 130.01, 129.56, 129.21, 128.51, 128.18, 126.98, 126.51, 125.78, 124.37, 121.48, 120.73, 118.78, 113.91. ESI-MS Calcd for [M + H]^+^ C_23_H_17_ClNO_3_S: 422.0618, found: 422.0610.

##### 4.1.3.12. (E)-1–(4-chlorophenyl)-3–(1-methyl-1H-indol-3-yl)prop-2-en-1-one (25a)

Yellow solid, yield 91%, m.p. 171–173 °C; ^1^H NMR (DMSO-d_6_, 300 MHz) *δ*: 8.25–8.03 (m, 5H, ArH), 7.84–7.81 (m, 1H, ArH), 7.66–7.52 (m, 3H, ArH,), 7.35–7.27 (m, 2H, CH), 3.84 (S, 3H, CH_3_), ^13 ^C NMR (101 MHz, Chloroform-*d*) δ 189.20, 139.12, 138.37, 138.31, 137.48, 134.85, 129.68, 128.74, 126.12, 123.27, 121.69, 120.75, 116.32, 112.96, 110.21, 33.29. ESI-MS Calcd for [M + H]^+^ C_18_H_15_ClNO: 296.0842, found: 296.0835.

##### 4.1.3.13. (E)-1–(4-chlorophenyl)-3–(1-ethyl-1H-indol-3-yl)prop-2-en-1-one (25 b)

Yellow solid, yield 79%, m.p. 165–168 °C; ^1^H NMR (DMSO-d_6_, 300 MHz) *δ*: 8.27–8.04 (m, 5H, ArH), 7.81–7.76 (m, 2H, ArH), 7.61–7.52 (m, 3H, ArH), 7.38–7.27 (m, 2H, CH), 4.31–4.20 (m, 2H, CH_2_), 1.45 (t, 3H, *J* = 4.51 Hz, CH_3_), ^13 ^C NMR (101 MHz, Chloroform-*d*) δ 188.29, 138.25, 137.33, 136.48, 136.37, 132.11, 128.66, 127.72, 125.29, 122.13, 120.63, 119.85, 115.26, 112.03, 109.25, 40.49, 14.16. ESI-MS Calcd for [M + H]^+^ C_19_H_17_ClNO: 310.0999, found: 310.0994.

##### 4.1.3.14. (E)-1–(4-chlorophenyl)-3–(1-propyl-1H-indol-3-yl)prop-2-en-1-one (25c)

Light yellow solid, yield 85%, m.p. 160–162 °C; ^1^H NMR (DMSO-d_6_, 300 MHz) *δ*: 8.11–8.01 (m, 5H, ArH), 7.86–7.84 (m, 2H, ArH), 7.62–7.54 (m, 3H, ArH,), 7.31–7.20 (m, 2H, CH), 4.25–4.22 (m, 2H, CH_2_), 1.84–1.82 (m, 2H, CH_2_), 0.89 (t, 3H, *J* = 4.52 Hz, CH_3_), ^13 ^C NMR (101 MHz, Chloroform-*d*) δ 189.28, 139.30, 138.36, 137.67, 137.51, 134.04, 129.70, 128.75, 126.27, 123.14, 121.62, 120.86, 116.28, 112.89, 110.45, 48.53, 23.24, 11.45. ESI-MS Calcd for [M + H]^+^ C_20_H_19_ClNO: 324.1155, found: 324.1152.

##### 4.1.3.15. (E)-3–(1-benzyl-1H-indol-3-yl)-1–(4-chlorophenyl)prop-2-en-1-one (25d)

red solid, yield 87%, m.p. 163–165 °C; ^1^H NMR (DMSO-d_6_, 300 MHz) *δ*: 8.32 (s, 1H, ArH), 8.20 (s, 1H, ArH), 8.15–8.04 (m, 3H, ArH), 7.83–7.81 (m, 1H, ArH), 7.66–7.52 (m, 3H, ArH, CH), 7.36–7.24 (m, 7H, ArH, CH) 5.50 (S, 2H, CH_2_), ^13 ^C NMR (101 MHz, Chloroform-*d*) δ 189.22, 139.01, 137.80, 137.34, 135.89, 134.02, 129.67, 128.97, 128.72, 128.12, 126.96, 126.33, 123.39, 121.81, 120.80, 116.74, 113.41, 110.71, 50.52. ESI-MS Calcd for [M + H]^+^ C_24_H_19_ClNO: 372.1155, found: 372.1153.

##### 4.1.3.16. (E)-1–(4-chlorophenyl)-3–(1-(phenylsulfonyl)-1H-indol-3-yl)prop-2-en-1-one (25e)

Yellow solid, yield 75%, m.p. 162–165 °C; ^1^H NMR (DMSO-d_6_, 300 MHz) *δ*: 8.70 (s, 1H, ArH), 8.32 (s, 1H, ArH), 8.23–8.13 (m, 2H, ArH), 8.21–7.83 (m, 6H, ArH), 7.56–7.52 (m, 1H, CH), 7.41–7.33 (m, 4H, ArH, CH), ^13 ^C NMR (101 MHz, Chloroform-*d*) δ 188.73, 139.17, 137.60, 136.38, 136.04, 135.60, 134.33, 129.82, 129.51, 129.04, 128.94, 128.17, 126.93, 125.71, 124.29, 121.48, 120.67, 118.79, 113.86. ESI-MS Calcd for [M + H]^+^ C_23_H_17_ClNO_3_S: 422.0618, found: 422.0606.

##### 4.1.3.17. (*E*)-1–(4-Methoxyphenyl)-3–(1-methyl-1H-indol-3-yl)prop-2-en-1-one (26a)

Yellow solid, yield 87%, m.p. 160–162 °C; ^1^H NMR (DMSO-d_6_, 300 MHz) *δ*: 8.02–7.98 (m, 5H, ArH), 7.69–7.55 (m, 2H, ArH), 7.34–7.26 (m, 2H, ArH), 7.10–7.08 (m, 2H, CH), 3.88–4.86 (m, 6H, CH_3_), ^13 ^C NMR (101 MHz, Chloroform-*d*) δ 188.95, 162.96, 138.25, 137.76, 134.27, 134.25, 131.97, 130.47, 126.20, 123.07, 121.44, 120.75, 116.81, 113.73, 113.05, 110.12, 55.47, 33.22. ESI-MS Calcd for [M + H]^+^ C_19_H_18_NO_2_: 292.1338, found: 292.1332.

##### 4.1.3.18. (*E*)-3–(1-Ethyl-1H-indol-3-yl)-1–(4-methoxyphenyl)prop-2-en-1-one (26 b)

Yellow solid, yield 73%, m.p. 165–168 °C; ^1^H NMR (DMSO-d_6_, 300 MHz) *δ*: 8.19–7.98 (m, 5H, ArH), 7.69–7.58 (m, 2H, ArH, CH), 7.33–7.25 (m, 2H, ArH), 7.11–7.06 (m, 2H, CH),4.36–4.25 (m, 2H, CH_2_), 3.88 (S, 3H, CH_3_), 1.43 (t, 3H, *J* = 4.502 Hz, CH_3_), ^13 ^C NMR (101 MHz, Chloroform-*d*) δ 188.98, 162.91, 137.87, 137.31, 132.54, 131.96, 130.46, 126.37, 122.94, 121.39, 120.86, 116.72, 113.69, 113.13, 110.17, 55.45, 41.42, 15.19. ESI-MS Calcd for [M + H]^+^ C_20_H_20_NO_2_: 306.1494, found: 306.1479.

##### 4.1.3.19. (*E*)-1–(4-Methoxyphenyl)-3–(1-propyl-1H-indol-3-yl)prop-2-en-1-one (26c)

Light yellow solid, yield 83%, m.p. 161–162 °C; ^1^H NMR (DMSO-d_6_, 300 MHz) *δ*: 8.17–7.98 (m, 5H, ArH), 7.69–7.61 (m, 2H, ArH), 7.32–7.25 (m, 2H, ArH), 7.10–7.05 (m, 2H, CH), 4.23–4.16 (m, 2H, CH_2_), 3.88 (S, 3H, CH_3_), 1.86–1.80 (m, 2H, CH_2_), 0.87 (t, 3H, *J* = 4.62 Hz, CH_3_), ^13 ^C NMR (101 MHz, Chloroform-*d*) δ 189.00, 162.94, 137.95, 137.60, 133.49, 131.97, 130.49, 126.33, 122.95, 121.38, 120.86, 116.72, 113.71, 112.96, 110.36, 55.48, 55.45, 48.46, 23.26, 11.46. ESI-MS Calcd for [M + H]^+^ C_21_H_22_NO_2_: 320.1651, found: 320.1642.

##### 4.1.3.20. (*E*)-3–(1-Benzyl-1H-indol-3-yl)-1–(4-methoxyphenyl)prop-2-en-1-one (26d)

red solid, yield 87%, m.p. 161–163 °C; ^1^H NMR (DMSO-d_6_, 300 MHz) *δ*: 8.31 (s, 1H, ArH), 8.17–8.09 (m, 3H, ArH), 8.05–8.00 (m, 1H, ArH), 7.72–7.69 (m, 1H, ArH), 7.62–7.59 (m, 1H, CH), 7.35–7.25 (m, 8H, ArH), 7.10–7.07 (m, 2H, CH), 5.50 (S, 2H, CH_2_), 3.88 (S, 3H, CH_3_), ^13 ^C NMR (101 MHz, Chloroform-*d*) δ 188.88, 162.92, 137.72, 137.61, 136.07, 133.47, 131.81, 130.47, 128.92, 128.02, 126.92, 123.19, 121.56, 120.79, 117.18, 113.68, 113.51, 110.62, 55.42, 50.42. ESI-MS Calcd for [M + H]^+^ C_25_H_22_NO_2_: 368.1651, found: 368.1643.

##### 4.1.3.21. (*E*)-1–(4-Methoxyphenyl)-3–(1-(phenylsulfonyl)-1H-indol-3-yl)prop-2-en-1-one (26e)

Yellow solid, yield 73%, m.p. 165–168 °C; ^1^H NMR (DMSO-d_6_, 300 MHz) *δ*: 8.66 (s, 1H, ArH), 8.20–8.16 (m, 2H, ArH), 8.01–7.92 (m, 5H, ArH), 7.45–7.38 (m, 4H, ArH, CH), 7.12–7.10 (m, 2H, CH), 3.88 (S, 3H, CH_3_), ^13 ^C NMR (101 MHz, Chloroform-*d*) δ 188.36, 163.46, 137.72, 135.66, 134.67, 134.28, 130.99, 130.76, 129.50, 128.47, 128.40, 126.93, 125.62, 124.21, 122.08, 120.73, 119.16, 113.90, 113.88, 55.50. ESI-MS Calcd for [M + H]^+^ C_24_H_19_NO_4_S: 418.1113, found: 418.1099.

##### 4.1.3.22. (*E*)-1–(3-Methoxyphenyl)-3–(1-methyl-1H-indol-3-yl)prop-2-en-1-one (27a)

Yellow solid, yield 85%, m.p. 161–162 °C; ^1^H NMR (DMSO-d_6_, 300 MHz) *δ*: 8.02–7.95 (m, 5H, ArH), 7.64–7.52 (m, 2H, ArH), 7.31–7.22 (m, 2H, ArH), 7.10–7.08 (m, 2H, CH), 3.88–3.86 (m, 6H, CH_3_), ^13 ^C NMR (101 MHz, Chloroform-*d*) δ 190.40, 159.83, 140.61, 138.68, 138.27, 134.58, 134.56, 129.42, 126.14, 123.17, 121.58, 120.77, 118.49, 117.06, 113.00, 112.87, 110.13, 55.48, 33.25. ESI-MS Calcd for [M + H]^+^ C_19_H_18_NO_2_: 292.1338, found: 292.1331.

##### 4.1.3.23. (*E*)-3–(1-Ethyl-1H-indol-3-yl)-1–(3-methoxyphenyl)prop-2-en-1-one (27 b)

Yellow solid, yield 72%, m.p. 165–166 °C; ^1^H NMR (DMSO-d_6_, 300 MHz) *δ*: 8.14–7.90 (m, 5H, ArH), 7.63––7.51 (m, 2H, ArH, CH), 7.34–7.26 (m, 2H, ArH), 7.14–7.08 (m, 2H, CH),4.39–4.26 (m, 2H, CH_2_), 3.88 (S, 3H, CH_3_), 1.41 (t, 3H, *J* = 4.502 Hz, CH_3_), ^13 ^C NMR (101 MHz, Chloroform-*d*) δ 189.41, 158.78, 139.60, 137.79, 136.32, 131.87, 128.38, 125.31, 122.03, 120.52, 119.87, 119.76, 117.46, 115.94, 112.06, 111.80, 54.46, 54.42, 40.44, 14.16. ESI-MS Calcd for [M + H]^+^ C_20_H_20_NO_2_: 306.1494, found: 306.1484.

##### 4.1.3.24. (*E*)-1–(3-Methoxyphenyl)-3–(1-propyl-1H-indol-3-yl)prop-2-en-1-one (27c)

Light yellow solid, yield 84%, m.p. 161–162 °C; ^1^H NMR (DMSO-d_6_, 300 MHz) *δ*: 8.19–7.93 (m, 5H, ArH), 7.67–7.60 (m, 2H, ArH), 7.34–7.28 (m, 2H, ArH), 7.10–7.07 (m, 2H, CH), 4.25–4.18 (m, 2H, CH_2_), 3.88 (S, 3H, CH_3_), 1.84–1.80 (m, 2H, CH_2_), 0.87 (t, 3H, *J* = 4.64 Hz, CH_3_), ^13 ^C NMR (101 MHz, Chloroform-*d*) δ 189.44, 158.78, 139.61, 137.82, 132.74, 128.39, 125.26, 122.01, 120.49, 119.87, 119.77, 117.49, 115.99, 111.91, 111.78, 109.33, 54.47, 47.48, 22.21, 10.42. ESI-MS Calcd for [M + H]^+^ C_21_H_222_NO_2_: 320.1651, found: 320.1644.

##### 4.1.3.25. (*E*)-3–(1-Benzyl-1H-indol-3-yl)-1–(3-methoxyphenyl)prop-2-en-1-one (27d)

Red solid, yield 64%, m.p. 161–163 °C; ^1^H NMR (DMSO-d_6_, 300 MHz) *δ*: 8.34 (s, 1H, ArH), 8.19–8.11 (m, 3H, ArH), 8.08–8.02 (m, 1H, ArH), 7.74–7.65 (m, 1H, ArH), 7.66–7.62 (m, 1H, CH), 7.37–7.28 (m, 8H, ArH), 7.12–7.09 (m, 2H, CH), 5.52 (S, 2H, CH_2_), 3.89 (S, 3H, CH_3_), ^13 ^C NMR (101 MHz, Chloroform-*d*) δ 190.34, 159.77, 140.47, 138.56, 137.76, 135.97, 133.82, 129.41, 128.94, 128.07, 126.94, 126.36, 123.29, 121.71, 120.83, 120.78, 118.52, 117.43, 113.46, 112.78, 110.65, 55.43, 50.46. ESI-MS Calcd for [M + H]^+^ C_25_H_22_NO_2_: 368.1651, found: 368.1640.

##### 4.1.3.26. (*E*)-1–(3-Methoxyphenyl)-3–(1-(phenylsulfonyl)-1H-indol-3-yl)prop-2-en-1-one (27e)

Yellow solid, yield 76%, m.p. 167–168 °C; ^1^H NMR (DMSO-d_6_, 300 MHz) *δ*: 8.68 (s, 1H, ArH), 8.22–8.18 (m, 2H, ArH), 8.02–7.96 (m, 5H, ArH), 7.47–7.39 (m, 4H, ArH, CH), 7.15–7.11 (m, 2H, CH), 3.85 (S, 3H, CH_3_), ^13 ^C NMR (101 MHz, Chloroform-*d*) δ 189.89, 159.92, 139.52, 137.68, 135.66, 135.63, 134.31, 129.62, 129.52, 128.89, 128.26, 126.94, 125.68, 124.28, 122.21, 120.97, 120.76, 119.25, 118.98, 113.87, 112.88. ESI-MS Calcd for [M + H]^+^ C_24_H_19_NO_4_S: 418.1113, found: 418.1099.

##### 4.1.3.27. (*E*)-3–(1-Methyl-1H-indol-3-yl)-1–(4-(trifluoromethyl)phenyl)prop-2-en-1-one (28a)

Yellow solid, yield 87%, m.p. 160–162 °C; ^1^H NMR (DMSO-d_6_, 300 MHz) *δ*: 8.30–8.29 (m, 2H, ArH), 8.16–8.05 (m, 3H, ArH), 7.94–7.91 (m, 2H, ArH), 7.68–7.58 (m, 2H, ArH, CH), 7.36–7.28 (m, 2H, CH), 3.87 (S, 3H, CH_3_), ^13 ^C NMR (101 MHz, Chloroform-*d*) δ 189.64, 142.19, 139.87, 138.37, 135.19, 135.17, 128.51, 126.10, 125.55, 125.51, 125.47, 123.39, 121.84, 120.77, 116.44, 112.95, 110.27, 33.33, 33.30. ESI-MS Calcd for [M + H]^+^ C_19_H_15_F_3_NO:330.1106, found: 330.1097.

##### 4.1.3.28. (*E*)-3–(1-ethyl-1H-indol-3-yl)-1–(4-(trifluoromethyl)phenyl)prop-2-en-1-one (28 b)

Yellow solid, yield 72%, m.p. 165–168 °C; ^1^H NMR (DMSO-d_6_, 300 MHz) *δ*: 8.30–8.07 (m, 5H, ArH), 7.92–7.90 (m, 2H, ArH), 7.66–7.61 (m, 2H, ArH, CH), 7.34–7.26 (m, 2H, CH), 4.30–4.24 (m, 2H, CH_2_), 1.44 (t, 3H, *J* = 4.51 Hz, CH_3_), ^13 ^C NMR (101 MHz, Chloroform-*d*) δ 189.69, 142.20, 140.02, 137.46, 133.52, 128.52, 126.29, 125.51, 125.47, 123.27, 121.81, 120.89, 116.33, 113.02, 110.36, 41.56, 15.16. ESI-MS Calcd for [M + H]^+^ C_20_H_17_F_3_NO: 344.1262, found: 344.1253.

##### 4.1.3.29. (*E*)-3–(1-Propyl-1H-indol-3-yl)-1–(4-(trifluoromethyl)phenyl)prop-2-en-1-one (28c)

Light yellow solid, yield 83%, m.p. 161–162 °C; ^1^H NMR (DMSO-d_6_, 300 MHz) *δ*: 8.31–8.06 (m, 5H, ArH), 7.92–7.8 (m, 2H, ArH), 7.67–7.62 (m, 2H, ArH, CH), 7.33–7.26 (m, 2H, CH), 1.87–1.78 (m, 2H, CH_2_), 0.86 (t, 3H, *J* = 4.62 Hz, CH_3_), ^13 ^C NMR (101 MHz, Chloroform-*d*) δ 189.71, 142.21, 140.05, 137.72, 134.38, 128.52, 126.24, 125.51, 125.47, 123.24, 121.76, 120.87, 116.37, 112.85, 110.50, 48.58, 23.22, 11.42. ESI-MS Calcd for [M + H]^+^ C_21_H_19_F_3_NO: 358.1419, found: 358.1418.

##### 4.1.3.30. (*E*)-3–(1-Benzyl-1H-indol-3-yl)-1–(4-(trifluoromethyl)phenyl)prop-2-en-1-one (28d)

Red solid, yield 87%, m.p. 160–163 °C; ^1^H NMR (DMSO-d_6_, 300 MHz) *δ*: 8.37–8.30 (m, 3H, ArH), 8.16–8.08 (m, 2H, ArH), 7.94–7.90 (m,2H, ArH), 7.70–7.59 (m, 2H, ArH, CH), 7.36–7.21 (m, 8H, ArH, CH), 5.52 (S, 2H, CH_2_), ^13 ^C NMR (101 MHz, Chloroform-*d*) δ 189.66, 142.05, 139.79, 137.88, 135.83, 134.41, 129.02, 128.52, 128.19, 127.01, 126.32, 125.50, 125.47, 123.51, 121.97, 120.84, 116.84, 113.39, 110.80, 50.57. ESI-MS Calcd for [M + H]^+^ C_25_H_19_F_3_NO: 406.1419, found: 406.1407.

##### 4.1.3.31. (E)-3–(1-(phenylsulfonyl)-1H-indol-3-yl)-1–(4-(trifluoromethyl)phenyl)prop-2-en-1-one (28e)

Yellow solid, yield 73%, m.p. 167–170 °C; ^1^H NMR (DMSO-d_6_, 300 MHz) *δ*: 8.31–8.29 (m, 3H, ArH), 8.18–8.11 (m, 4H, ArH), 7.96–7.11 (m, 3H, ArH), 7.67–7.52 (m, 3H, ArH, CH), 7.29–7.24 (m, 3H, ArH, CH), ^13 ^C NMR (101 MHz, Chloroform-*d*) δ 189.30, 137.64, 136.92, 135.68, 134.41, 129.57, 129.43, 128.72, 128.10, 126.99, 125.83, 125.74, 125.70, 124.40, 121.56, 120.71, 118.68, 113.94. ESI-MS Calcd for [M + H]^+^ C_24_H_17_F_3_NO_3_S: 456.0881, found: 456.0873.

##### 4.1.3.32. (*E*)-3–(1-Methyl-1H-indol-3-yl)-1–(3,4,5-trimethoxyphenyl)prop-2-en-1-one (29a)

Yellow solid, yield 87%, m.p. 160–162 °C; ^1^H NMR (DMSO-d_6_, 300 MHz) *δ*: 8.18 (S, 1H, ArH), 8.09–8.01 (m, 5H, ArH), 7.65–7.55 (m, 2H, ArH, CH), 7.38–7.25 (m, 4H, ArH, CH), 3.93–3.87 (m, 9H, CH_3_), 3.77 (m, 3H, CH_3_), ^13 ^C NMR (101 MHz, Chloroform-*d*) δ 189.53, 153.07, 138.53, 138.25, 134.57, 134.44, 134.42, 126.17, 123.15, 121.56, 120.56, 116.78, 112.93, 110.18, 105.95, 60.95, 56.41, 56.37, 33.25. ESI-MS: m/z 351.12 (M^+^). Anal. Calcd for C_21_H_11_NO_4_: C, 71.78; H, 6.09; N, 3.96. ESI-MS Calcd for [M + H]^+^ C_21_H_22_NO_4_: 352.1549, found: 352.1535.

##### 4.1.3.33. (*E*)-3–(1-Ethyl-1H-indol-3-yl)-1–(3,4,5-trimethoxyphenyl)prop-2-en-1-one (29 b)

Yellow solid, yield 73%, m.p. 155–158 °C; ^1^H NMR (DMSO-d_6_, 300 MHz) *δ*: 8.23 (S, 1H, ArH), 8.09–8.01 (m, 2H, ArH), 7.65–7.61 (m, 2H, ArH), 7.38–7.25 (m, 3H, ArH, CH), 4.32–4.26 (m, 2H, CH_2_), 3.92 (m, 6H, CH_3_), 3.78 (m, 3H, CH_3_), 1.45 (t, 3H, *J* = 4.52 Hz, CH_3_), ^13 ^C NMR (101 MHz, Chloroform-*d*) δ 189.63, 153.05, 138.70, 137.32, 134.61, 132.79, 126.35, 123.05, 121.54, 120.70, 116.70, 113.02, 110.27, 105.86, 60.97, 56.39, 56.36, 41.48, 15.20. ESI-MS Calcd for [M + H]^+^ C_22_H_24_NO_4_: 366.1705, found: 366.1692.

##### 4.1.3.34. (*E*)-3–(1-Propyl-1H-indol-3-yl)-1–(3,4,5-trimethoxyphenyl)prop-2-en-1-one (29c)

Light yellow solid, yield 85%, m.p. 162–164 °C; ^1^H NMR (DMSO-d_6_, 300 MHz) *δ*: 8.23 (S, 1H, ArH), 8.09–8.01 (m, 2H, ArH), 7.66–7.62 (m, 2H, ArH, CH), 7.38–7.23 (m, 4H, ArH, CH), 4.23–4.20 (m, 2H, CH_2_), 3.92 (m, 6H, CH_3_), 3.78 (m, 3H, CH_3_), 1.87–1.81 (m, 3H, CH_2_), 0.87 (t, 3H, *J* = 4.502 Hz, CH_3_), ^13 ^C NMR (101 MHz, Chloroform-*d*) δ 188.63, 152.04, 137.70, 136.59, 133.59, 132.63, 125.27, 122.01, 120.48, 119.67, 115.72, 111.85, 109.39, 104.87, 59.95, 55.38, 55.35, 47.46, 22.21, 10.40. ESI-MS Calcd for [M + H]^+^ C_23_H_26_NO_4_: 380.1862, found: 380.1858.

##### 4.1.3.35. (*E*)-3–(1-Benzyl-1H-indol-3-yl)-1–(3,4,5-trimethoxyphenyl)prop-2-en-1-one (29d)

red solid, yield 85%, m.p. 160–163 °C; ^1^H NMR (DMSO-d_6_, 300 MHz) *δ*: 8.38 (s, 1H, ArH), 8.11–8.03 (m, 2H, ArH), 7.70–7.58 (m, 2H, ArH), 7.39–7.24 (m, 11H, ArH, CH), 5.50 (S, 2H, CH_2_), 3.92 (m, 6H, CH_3_), 3.78 (m, 3H, CH_3_), ^13 ^C NMR (101 MHz, Chloroform-*d*) δ 189.54, 153.05, 138.44, 137.77, 136.01, 134.48, 133.70, 128.96, 128.09, 126.92, 126.41, 123.31, 121.72, 120.66, 117.20, 113.44, 110.72, 105.87, 60.95, 56.38, 56.35, 50.49. ESI-MS Calcd for [M + H]^+^ C_27_H_26_NO_4_: 428.1862, found: 428.1852.

##### 4.1.3.36. (*E*)-3–(1-(Phenylsulfonyl)-1H-indol-3-yl)-1–(3,4,5-trimethoxyphenyl)prop-2-en-1-one (29e)

Yellow solid, yield 74%, m.p. 162–165 °C; ^1^H NMR (DMSO-d_6_, 300 MHz) *δ*: 8.66 (s, 1H, ArH), 8.12–7.94 (m, 6H, ArH), 7.47–7.39 (m, 6H, ArH, CH), 3.92 (m, 6H, CH_3_), 3.78 (m, 3H, CH_3_), ^13 ^C NMR (101 MHz, Chloroform-*d*) δ 189.00, 153.21, 137.71, 135.64, 135.42, 134.34, 133.45, 129.54, 128.51, 126.95, 125.70, 124.98, 124.28, 122.02, 120.57, 118.97, 113.92, 106.17, 61.05, 56.50. ESI-MS Calcd for [M + H]^+^ C_26_H_24_NO_6_S: 478.1345, found: 478.1271.

### 4.2. Biological section

#### 4.2.1. Cck-8 assay *in vitro*

Cells were seeded in 96-well plates at a density of 8 × 10^3^ per well and exposed to different concentrations of compounds for 72 h. Subsequently, 10 µL CCK8 solution was added to each well and co-incubated for another 3–4 h at 37 °C. The absorbance was determined at 450 nm. Data was calculated with GraphPad Prism5 software.^32^

#### 4.2.2. Cell apoptosis assay

K562 cells at 8х10^4^ cells/well were seeded in 12-well cell culture plates overnight and the cultured cells were incubated with a dose range of compounds **29e** for 48 h. The treated or untreated cells were washed twice with PBS and stained by Annexin V-PE and PI for 15 min without light exposure according to the manufacturer’s protocol. Apoptosis was quantified using a flow cytometer ((Becton, Dickinson and Company, USA).^32^

#### 4.2.3. Western blot assay

Exponentially growing K562 cells in were seeded in 10 mm dishes at a density of 6х10^5^/dish and incubated overnight. The cultured cells were treated with indicated concentrations of **29e** for 48 h. After the cells were washed with cold PBS, the supernatant was removed. Cells were scraped off the tissue culture dish, and then lysed by 100 µl ice-cold RIPA lysis buffer for 20 min with occasional agitation. The supernatant was collected by centrifuging at 12,000 g for 10 min at 4 °C. The protein concentration in the supernatant was determined by using a BCA protein assay kit. After BCA analysis to quantify proteins, samples were prepared in SDS-PAGE loading buffer, then boiled for 10 min at 100 °C. Western blot analyses were conducted after separation by SDS-PAGE electrophoresis and transfer to nitrocellulose filter (NC) membranes. Immunoblotting was performed according to the antibody manufacturers’ recommendations. Antibodies: caspase-3, caspase-9, PARP and β-actin.

#### 4.2.4. Tube formation assay

Matrigel matrix (Basement Membrane Matrix, BD Biosciences) was thawed at 4 °C overnight. At this point, 50 µL of the matrix solution was added to each well of a 96-well plate. After gelling at 37 °C for 30 min, the matrix was overlaid with 200 µL of medium containing 1 × 10^5^ HUVECs per well, which was incubated for 6 h to allow capillary tubes to form. Different concentrations of compounds were added in the cultures and incubated for 6 h to monitor the morphological changes of cells and tubes. The disappearance of existing vasculature was photographed at 10х magnification. Values were expressed as percent change form control cultures grown with complete medium.^36^ Standard dimensional parameters (percent area covered by HUVECs and total length of the HUVEC network per field) were noted, and standard topological parameters (number of meshes and branching points per field) were estimated.

#### 4.2.5. Would healing migration assays

K562 cells were seeded in 6-well plates and cultivated for 24 h. Scratches were made in confluent monolayer using 200 µL pipette tips and washed with PBS to remove the detached cells. The media containing different concentrations of compounds were added to the scratched monolayers. The migrated cells were photographed under a light microscope at indicated time points from the scratch. The migration distance of cells migrated to the wound area was measured manually and compared with the initial distance.

#### 4.2.6. Transwell cell invasion experiment

The upper surface of Transwell membrane was precoated with 40 µL Matrigel gel and incubated for 45 min at 37 °C for gelling. Then, cells were trypsinized and seeded at 6х10^4^ per upper chamber, and cultured in 1% foetal bovine serum medium containing different concentrations of compounds. The chamber was then placed in a 24-well plate to which 10% FBS had been added. After 48 h incubation at 37 °C, both chambers were washed with PBS three times, and then fixed with 4% paraformaldehyde for 15 min, followed by staining with crystal violet. The non-invasive cells on the upper surface membrane were wiped with cotton swabs. The image of the cells on the bottom face was recorded and quantified under an inverted microscope. The results were the means calculated from three replicates of each experiment.^37^

#### 4.2.7. Zebrafish angiogenesis assay

The transgenic flk: enhanced GFP zebrafish embryos were generated by pair-wise mating, collected and sorted into the 6-well plate (*n* = 30 per well) with 1 ml of aquaculture water (0.2 g/L of Instant Ocean Salt in distilled water) and raised at 28 °C. Then the embryos staged at 12 h post fertilisation (hpf) were treated with the indicated concentrations of compounds which were added into embryo water. About 30 embryos were screened for each concentration gradient. At 30hpf, the treated embryos were anaesthetised with 0.02% tricaine in embryo water and photographed. The numbers of ISVs were recorded under confocal microscopy. Results were obtained from three independent determinations and presented as mean ± SD.^38^

#### 4.2.8. Tubulin polymerisation assay

Tubulin polymerisation was determined with the tubulin polymerisation assay kit (Cytoskeleton, Denver, CO which was purchased from Cytoskeleton, Beyotime, China. Tubulin isolated from porcine brain tissue was used in this commercial kit.) It is based on the principal that light is scattered by microtubules to an extent that is proportional to the concentration of the microtubule polymer. Compounds that interact with tubulin will alter the polymerisation of tubulin, and this can be detected using a spectrophotometer. Tubulin polymerisation dynamics was monitored through measuring the change of absorbance at 340 nm every 2 min for 30 min at 37 °C on a spectrophotometer.

#### 4.2.9. [^3^h] colchicine–tubulin binding assay

One micromolar radiolabeled colchicine [ring C, Methoxy-3H] (Perkin-Elmer), 1% DMSO and various concentrations of test compounds in 50 µL G-PEM buffer containing 80 mmol/L PIPES (pH 6.8), 1 mmol/L EGTA, 1 mmol/L MgC_l2_ and 1 mmol/L GTP, and 5% glycerol were incu-bated with 1 mmol/L tubulin (> 99% pure; Cytoskeleton, Inc.; 0.2 mg/mL) at 37 °C for 60 min. The binding solutions were filtered through a stack of 2 DEAE-cellulose filters and washed twice. The radioactivity in the filtrates was determined by liquid scintillation spectrometry (Perkin-Elmer Wallac). Nonlinear regression was used to analyse the data using GraphPad Prism.

#### 4.2.10. Molecular Docking

Molecular docking of compounds into the three dimensional X-ray structure of tubulin (PDB code: 1SA0) was carried out using the MOE 2015. During the docking simulations, all the side chains of residues surrounding the defined binding site were regarded as rotatable bonds, and the ligand and its single bonds were allowed to move freely within the potential binding pocket. After the docking simulations, the 20 best-scored ligand–protein complexes of each ligand were kept for further analyses.

#### 4.2.11. Zebrafish xenografts injection and treatment

K562 cells were labelled with Red CMTPX according to the manufacturer’s protocol. Before the microinjections, Tg(flk:EGFP) embryos were kept at 28 °C and manually dechorionated few hours. At 48 hpf, 4 nL of cells suspension containing 400 labelled cells was microinjected into the yolk of anaesthetised embryos by a pneumatic pico pump (PV820, World Precision Instruments, USA). After injection, embryos were incubated to recover for at least 1 h at 28 °C, the embryos were distributed to 6-well plates with 20 embryos placed in each well. The injected xenografts were exposed to the indicated doses of compounds and maintained at 35 °C. DMSO (0.3%) was used as a vehicle control. The survival and development of the xenografted embryos was recorded every day until the end of experiment. At one day post injection (dpi), fluorescent microscopy was used to examine K562 cells size, death and migration in caudal region of anaesthetised zebrafish.

#### 4.2.12. Physicochemical experiment

Calculated physicochemical parameters. Physicochemical properties were calculated using the ChemAxon chemistry cartridge via JChem for Excel software (version 16.4.11). Compound **29e** in DMSO was spiked into either pH 6.5 phosphate buffer or 0.01 M HCl (approximately pH 2.0) with the final DMSO concentration being 1%. After 30 min had elapsed, samples were analysed via Nephelometry to determine a solubility range.

#### 4.2.13. *In vitro* metabolic stability

The metabolic stability assay was performed by incubating the test compound in mouse or human liver microsomes (XenoTech, lot no. 1510256) at 37 °C and a protein concentration of 0.4 mg/mL. Microsomal incubations were performed at a substrate concentration of 0.5 µM. Data analysis. Species scaling factors were used to convert the *in vitro* CLint(mL/min/mg) to an in vivo CLint(mL/min/kg).

## Supplementary Material

Supplemental Material
