# Supplementary material for: Angiogenesis and anti-leukaemia activity of novel indole derivatives as potent colchicine binding site inhibitors
Source: J Enzyme Inhib Med Chem. 2022 Feb 2;37(1):652–65. doi: 10.1080/14756366.2022.2032688 (PMC8820799; doi:10.1080/14756366.2022.2032688)
Supplement: Supplemental Material [file IENZ_A_2032688_SM8029.pdf]

1. The promising and representative screening result for another 130 compounds (inhibiting tubulin polymerization: IC_{50}<100 μM and anti-angiogenesis in Zebrafish: IC_{50}<100 μM) based on our library

Cpd.	Tubulin[a](μM)	Zebrafish[a](μM)
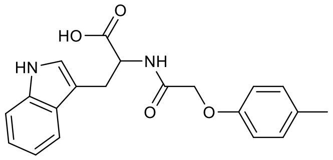	30.2±1.7	65.3±1.5
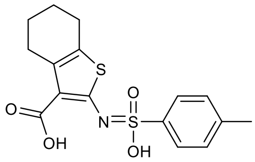	35.6±3.5	53.2±1.4
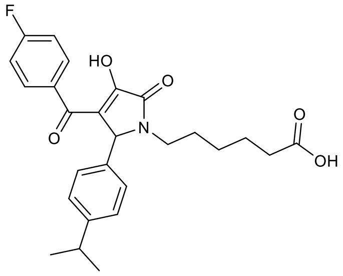	24.3±1.2	85.3±2.6
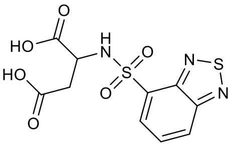	39.6±3.4	66.3±3.8
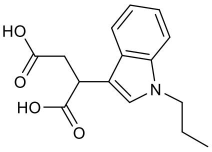	9.0±1.7	ND[b]
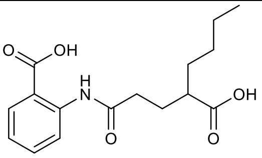	65.3±1.2	25.3±0.3

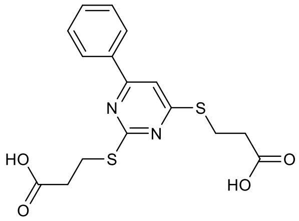	81.2±0.5	89.3±2.2
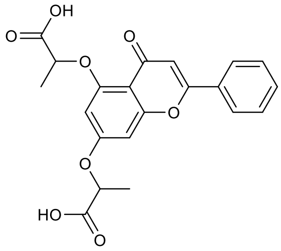	63.2±0.3	74.3±3.4
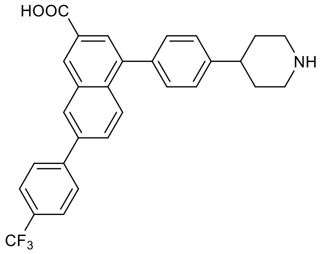	10.6±3.2	95.3±14.1
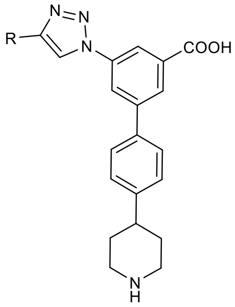	43.9±3.8	75.3±2.8
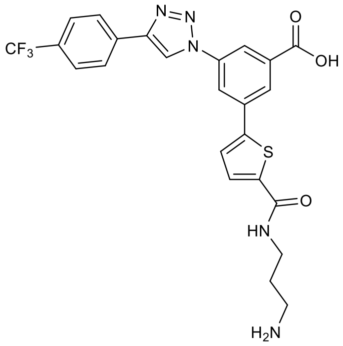	12.3±0.6	35.8±2.1

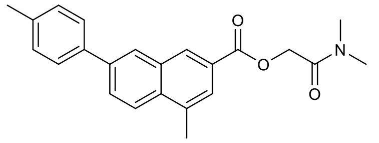	35.4±3.4	74.2±3.6
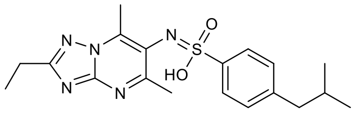	96.3±2.6	45.3±2.5
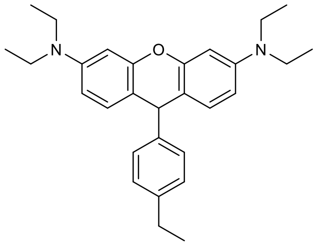	38.9±1.5	10.5±3.7
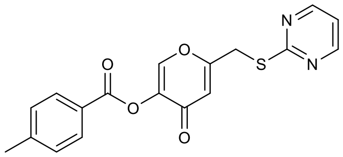	25.8±2.7	54.6±0.6
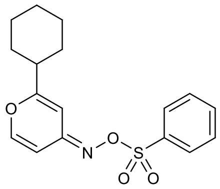	52.3±0.9	42.3±1.7
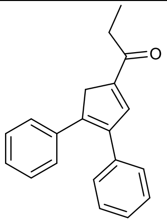	36.5±2.4	53.4±3.5
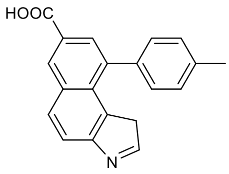	64.8±4.2	24.8±3.4

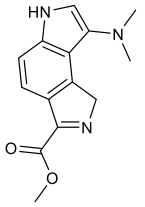	65.4±2.5	75.3±1.2
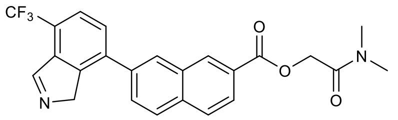	64.2±2.4	67.5±3.5
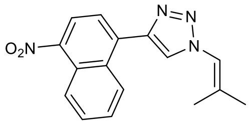	74.9±0.2	98.3±3.1
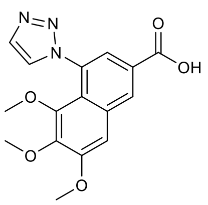	36.5±1.3	46.5±2.0
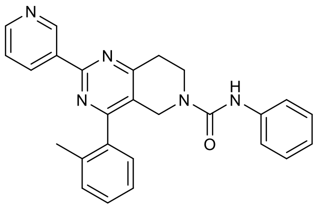	96.3±0.5	80.1±2.3
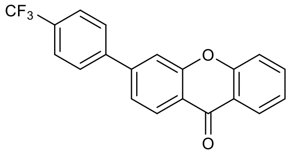	41.3±3.4	75.3±2.4
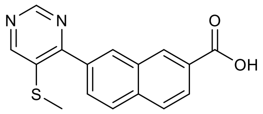	64.2±0.6	75.3±2.6

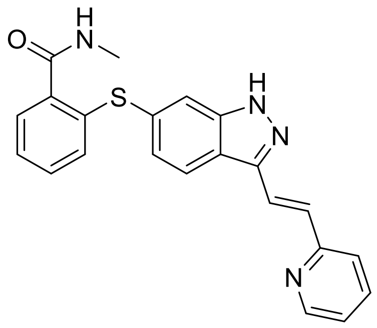	38.9±1.5	10.5±3.7
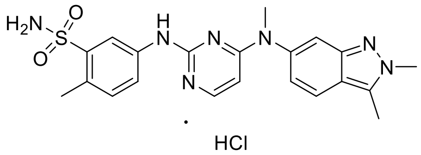	36.3±3.8	65.3±1.5
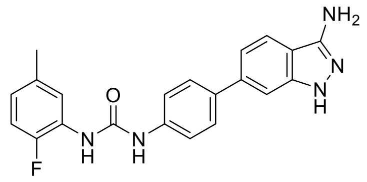	ND[b]	53.2±1.4
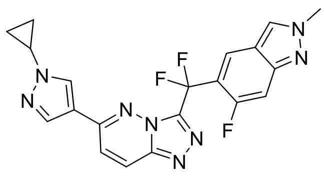	25.3±0.3	45.3±2.6
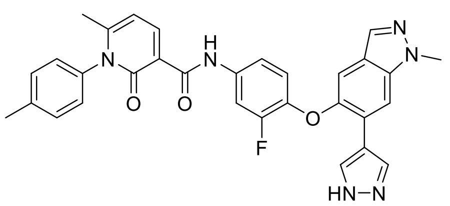	89.3±2.2	36.3±3.8
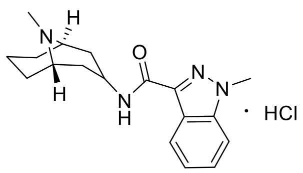	74.3±3.4	ND[b]

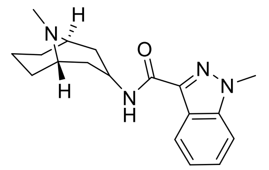	23.3±2.4	25.3±0.3
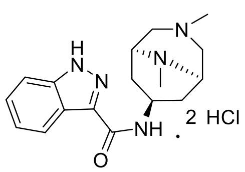	75.3±2.8	89.3±2.2
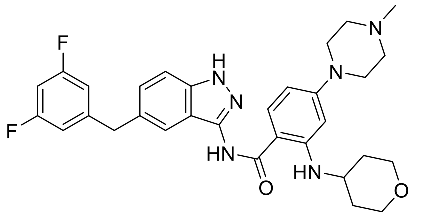	35.8±2.1	74.3±3.4
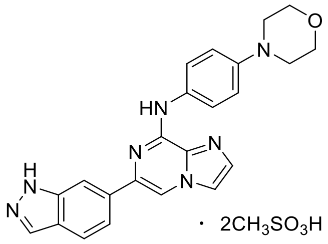	74.2±3.6	23.3±2.4
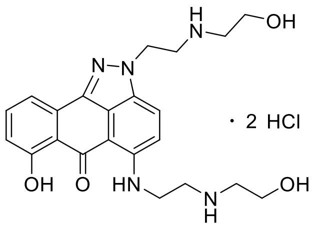	45.3±2.5	75.3±2.8
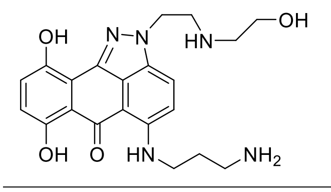	10.5±3.7	55.8±2.1

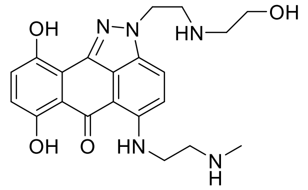	54.6±0.6	74.2±3.6
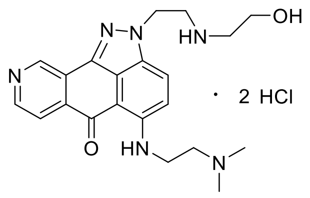	42.3±1.7	45.3±2.5
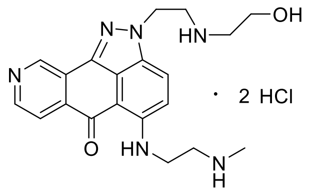	53.4±3.5	10.5±3.7
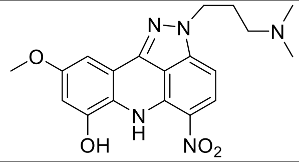	24.8±3.4	54.6±0.6
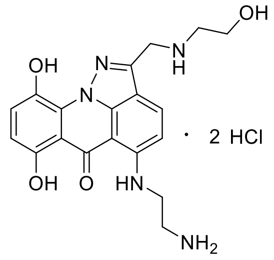	45.3±2.5	42.3±1.7
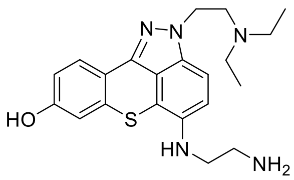	10.2±3.7	53.4±3.5

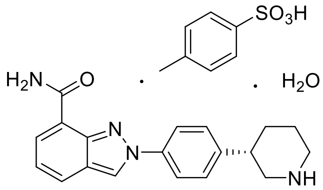	54.6±0.6	24.8±3.4
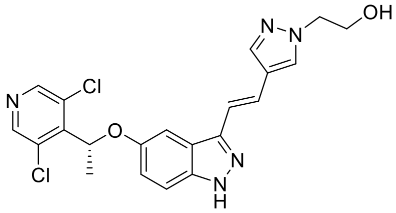	42.3±1.7	75.3±1.2
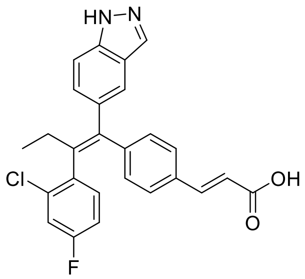	53.4±3.5	67.5±3.5
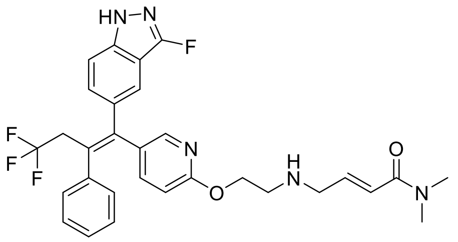	24.8±3.4	98.3±3.1
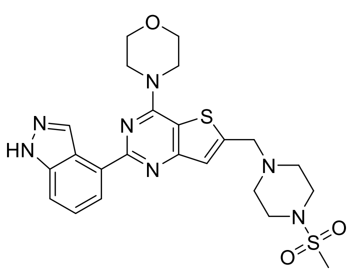	75.3±1.2	46.5±2.0

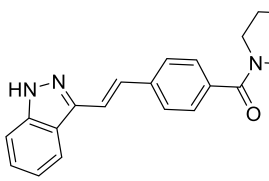	67.5±3.5	80.1±2.3
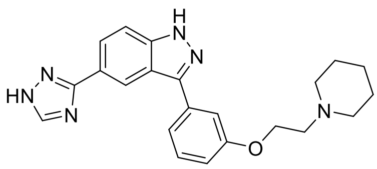	98.3±3.1	75.3±2.4
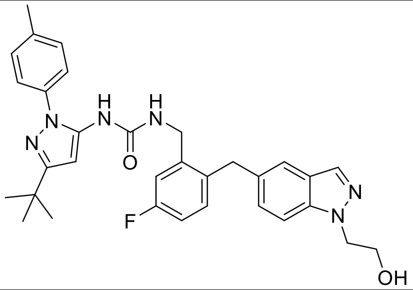	46.5±2.0	75.3±2.6
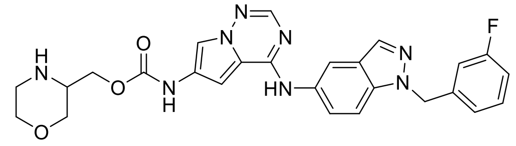	80.1±2.3	10.5±3.7
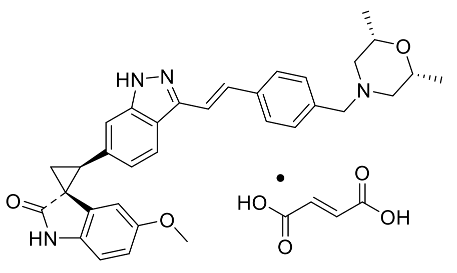	75.3±2.4	65.3±1.5
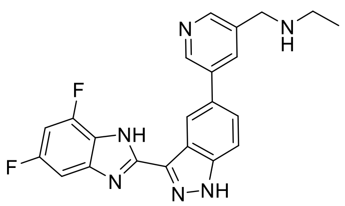	75.3±2.6	53.2±1.4

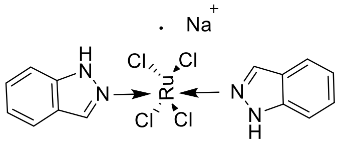	10.5±3.7	45.3±1.6
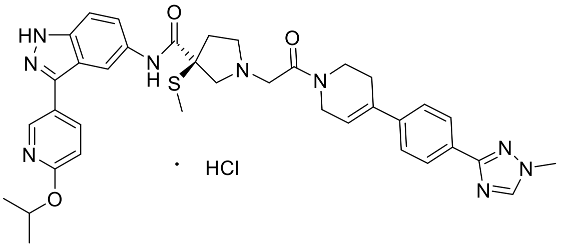	65.3±1.5	36.3±3.8
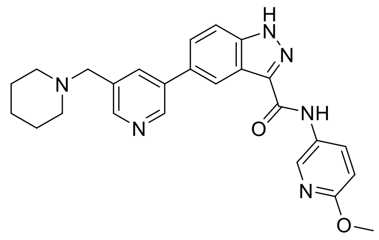	53.2±1.4	ND[b]
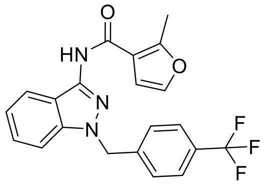	45.3±2.5	25.3±0.3
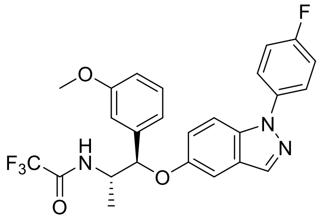	10.5±3.7	89.3±2.2
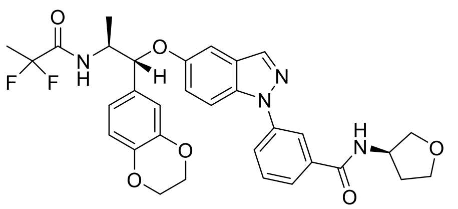	54.6±0.6	74.3±3.4

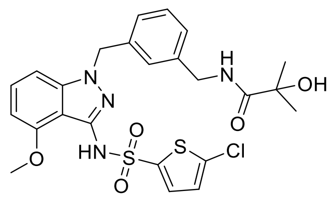	42.3±1.7	23.3±2.4
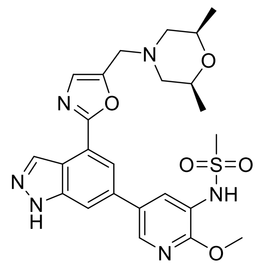	53.4±3.5	65.3±1.5
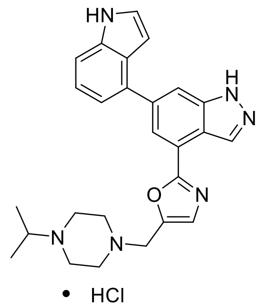	24.8±3.4	53.2±1.4
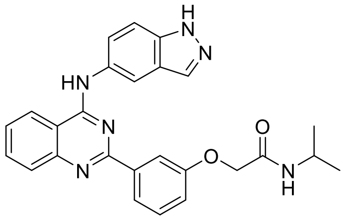	75.3±1.2	45.3±2.6
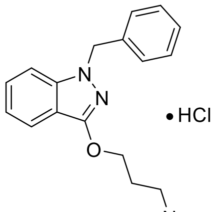	67.5±3.5	36.3±3.8

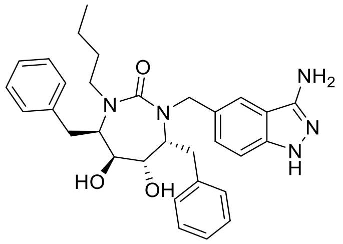	98.3±3.1	ND[b]
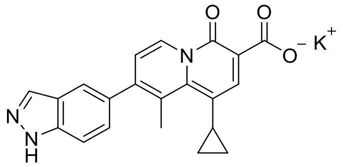	46.5±2.0	25.3±0.3
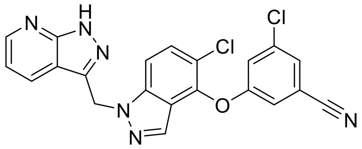	80.1±2.3	89.3±2.2
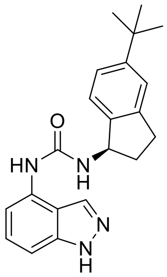	75.3±2.4	74.3±3.4
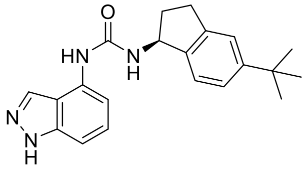	75.3±2.6	23.3±2.4
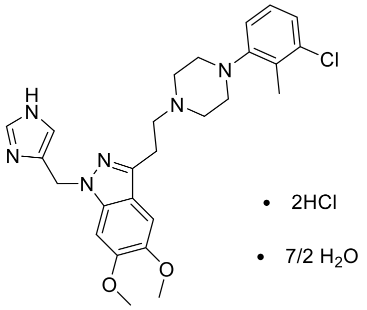	24.3±1.2	75.3±2.8

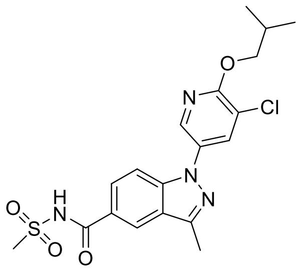	32.6±3.4	35.8±2.1
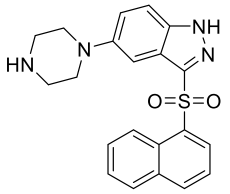	9.0±1.7	74.2±3.6
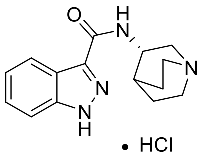	65.3±1.2	45.3±2.5
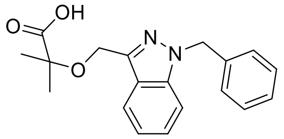	81.2±0.5	10.5±3.7
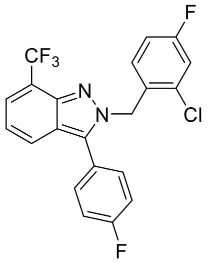	63.2±0.3	54.6±0.6
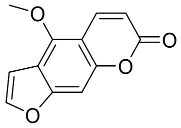	10.6±3.2	42.3±1.7

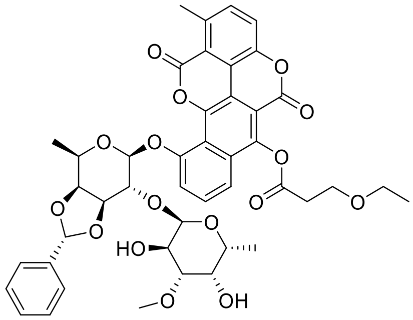	43.9±3.8	53.4±3.5
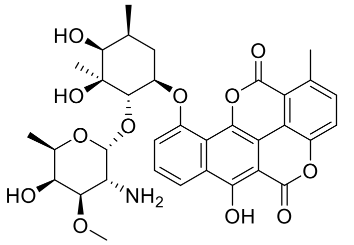	12.3±0.6	24.8±3.4
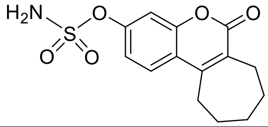	35.4±3.4	75.3±1.2
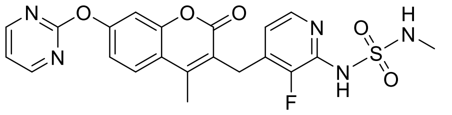	96.3±2.6	67.5±3.5
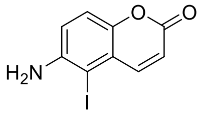	38.9±1.5	98.3±3.1
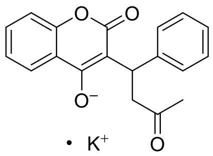	24.3±1.2	46.5±2.0
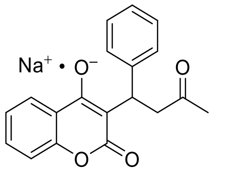	32.6±3.4	80.1±2.3

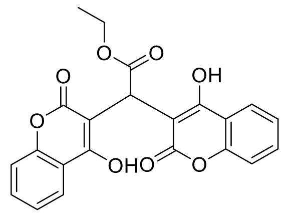	54.6±0.6	65.3±1.5
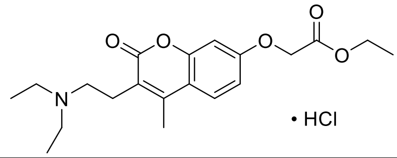	42.3±1.7	53.2±1.4
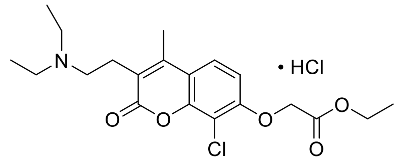	53.4±3.5	45.3±2.6
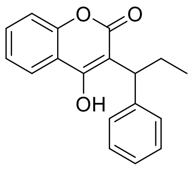	24.8±3.4	36.3±3.8
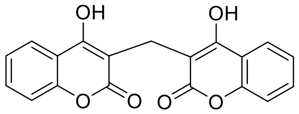	75.3±1.2	ND[b]
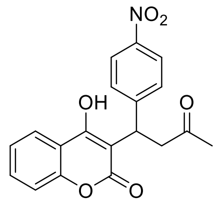	67.5±3.5	25.3±0.3
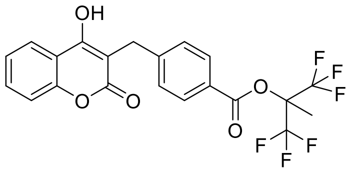	98.3±3.1	89.3±2.2

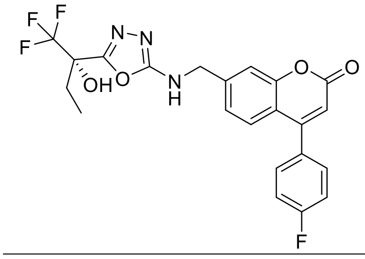	46.5±2.0	74.3±3.4
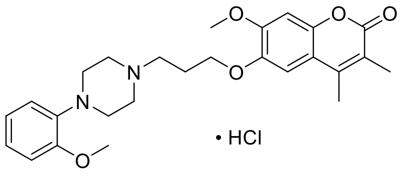	80.1±2.3	23.3±2.4
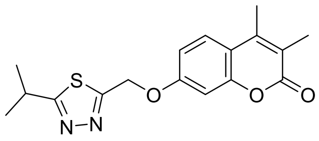	75.3±2.4	75.3±2.8
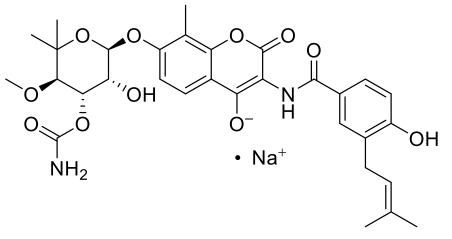	75.3±2.6	35.8±2.1
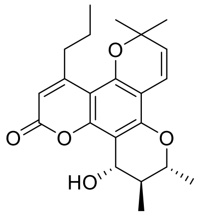	10.5±3.7	74.2±3.6
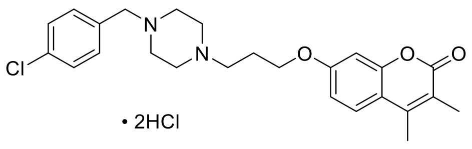	65.3±1.5	45.3±2.5
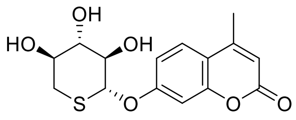	53.2±1.4	10.5±3.7

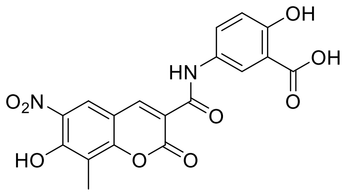	65.3±1.5	54.6±0.6
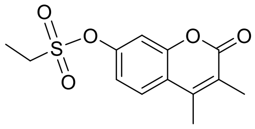	53.2±1.4	42.3±1.7
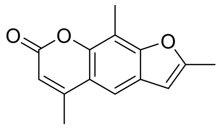	54.6±0.6	53.4±3.5
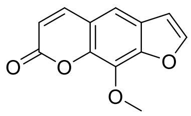	42.3±1.7	24.8±3.4
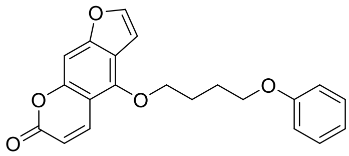	53.4±3.5	75.3±1.2
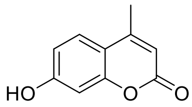	24.8±3.4	67.5±3.5
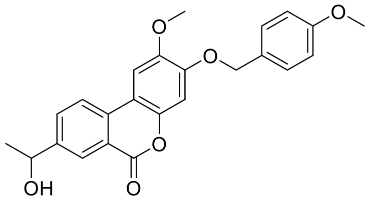	75.3±1.2	98.3±3.1
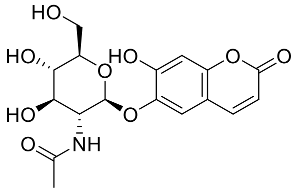	67.5±3.5	46.5±2.0

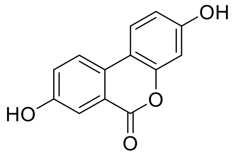	98.3±3.1	80.1±2.3
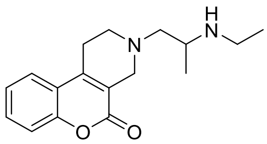	46.5±2.0	75.3±2.4
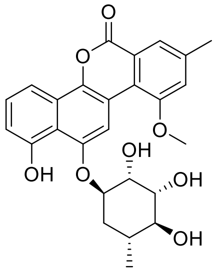	80.1±2.3	75.3±2.6
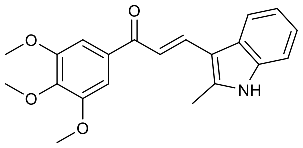	75.3±2.4	10.5±3.7
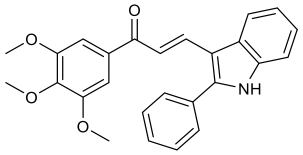	75.3±2.6	65.3±1.5
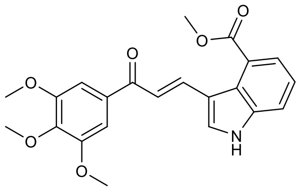	10.5±3.7	53.2±1.4
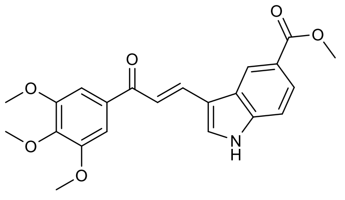	65.3±1.5	65.3±1.5

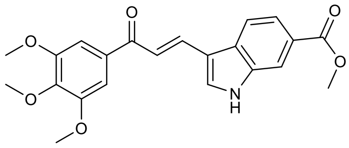	54.6±0.6	53.2±1.4
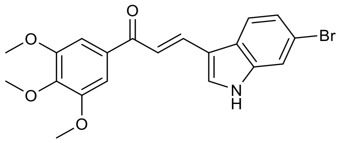	42.3±1.7	45.3±2.6
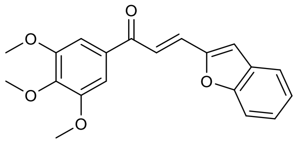	53.4±3.5	36.3±3.8
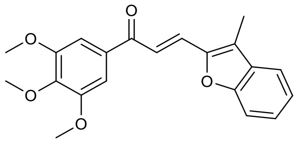	24.8±3.4	ND[b]
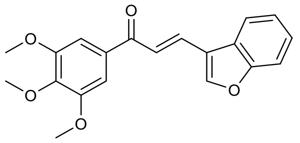	75.3±1.2	25.3±0.3
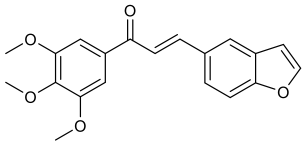	67.5±3.5	89.3±2.2
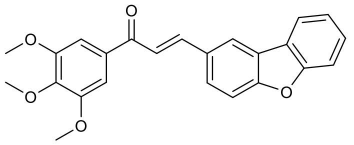	98.3±3.1	74.3±3.4
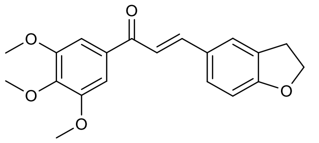	46.5±2.0	23.3±2.4

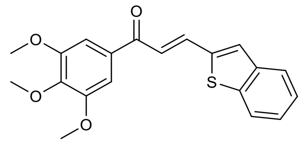	80.1±2.3	75.3±2.8
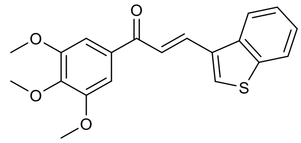	75.3±2.4	35.8±2.1
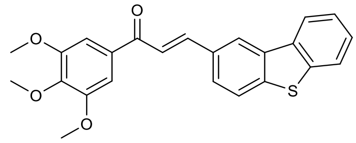	75.3±2.6	74.2±3.6
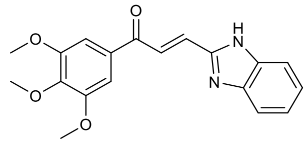	54.6±0.6	45.3±2.5
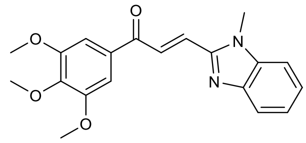	42.3±1.7	10.5±3.7
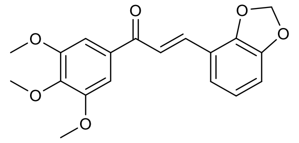	53.4±3.5	54.6±0.6
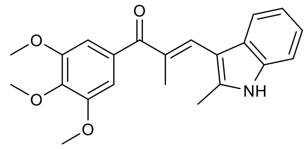	24.8±3.4	42.3±1.7
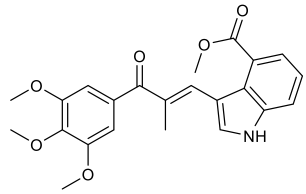	75.3±1.2	53.4±3.5

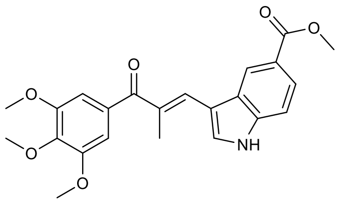		67.5±3.5	62

[a]Each compound was tested in triplicate; the data are presented as the mean ± SD.

[b]ND: not detected because of its strong toxicity.

2. ^{1}H-NMR of all the compounds

^{1}H-NMR of Compound of **DYT-1**

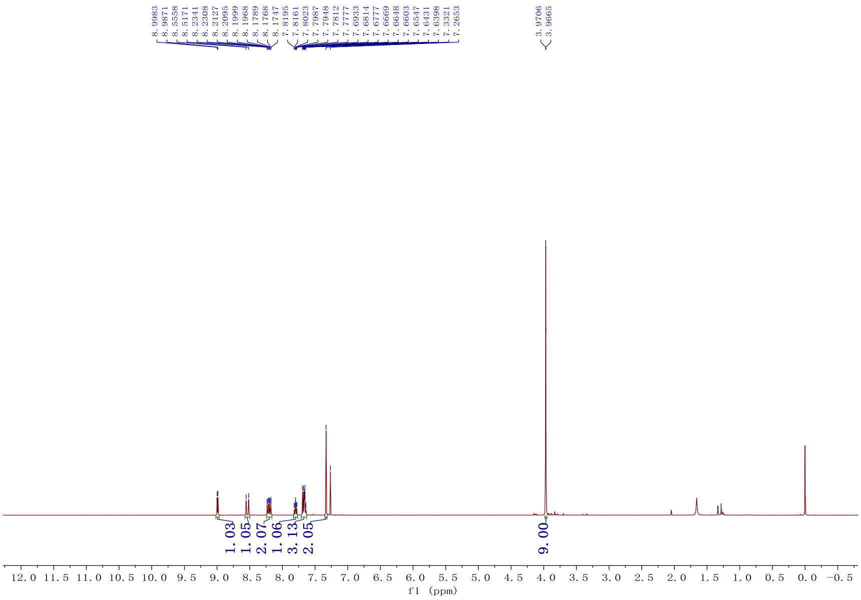

^{1}H-NMR of Compound **6**

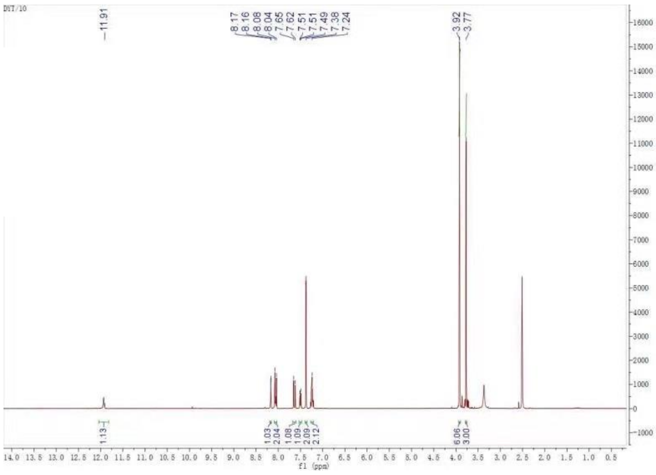

^{1}H-NMR of Compound **7**

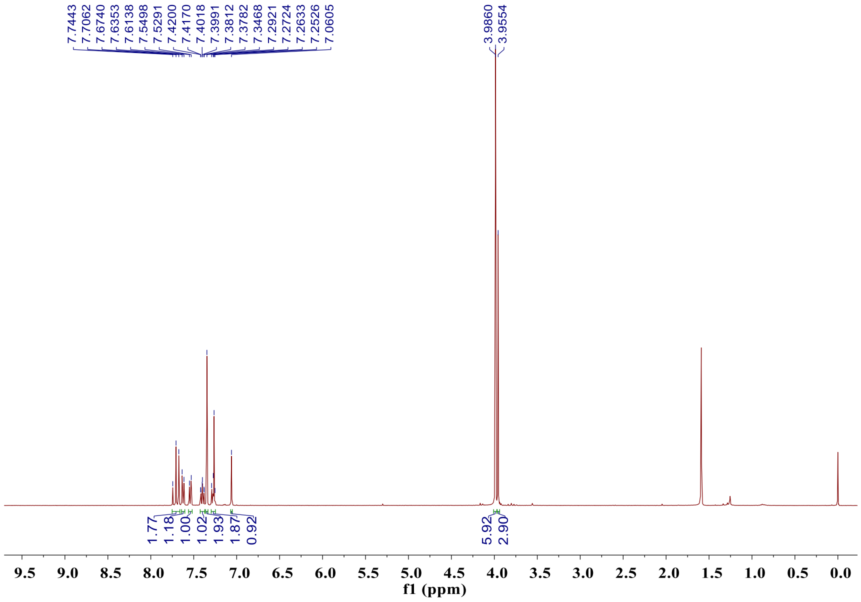

^{1}H-NMR of Compound **8:**

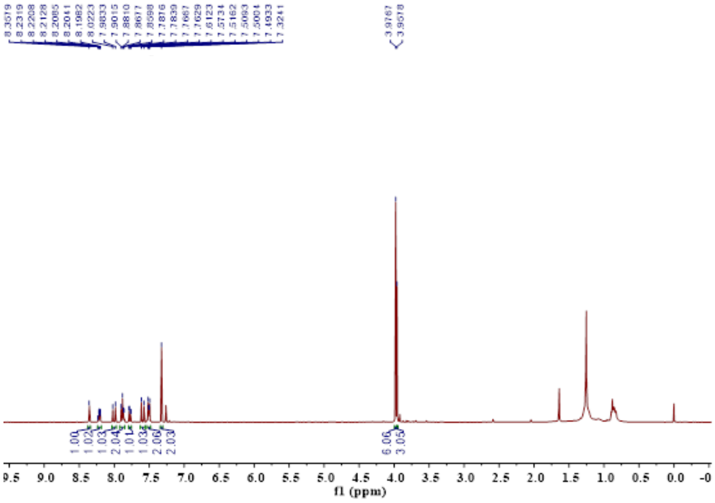

^{1}H-NMR of Compound **9:**

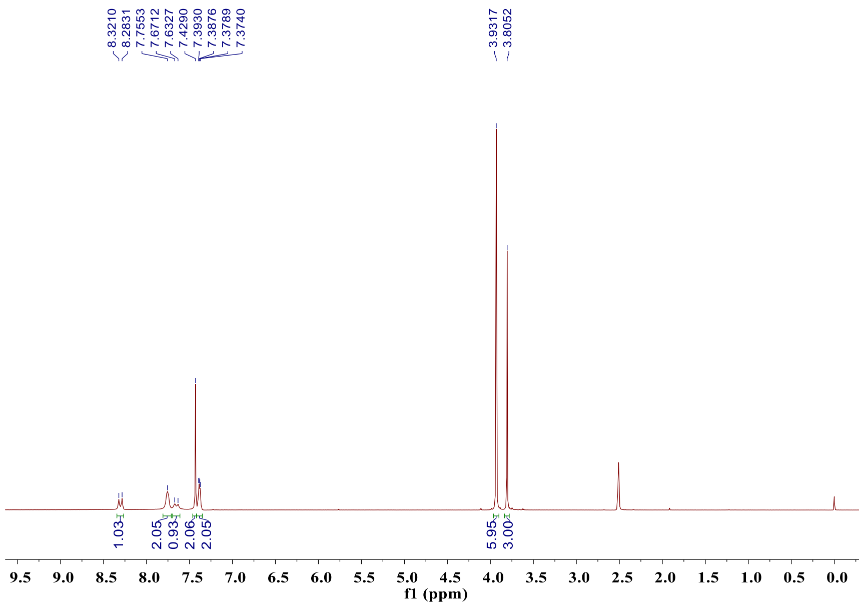

^{1}H-NMR of Compound of **23a**

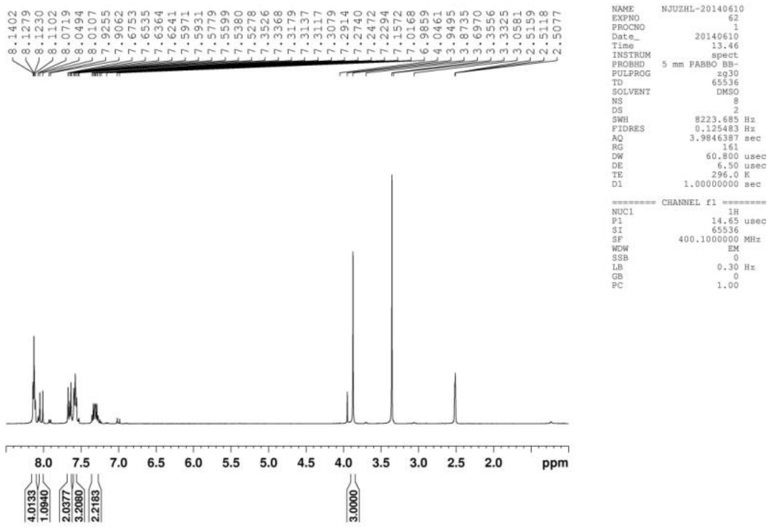

^{1}H-NMR of Compound of **23b**

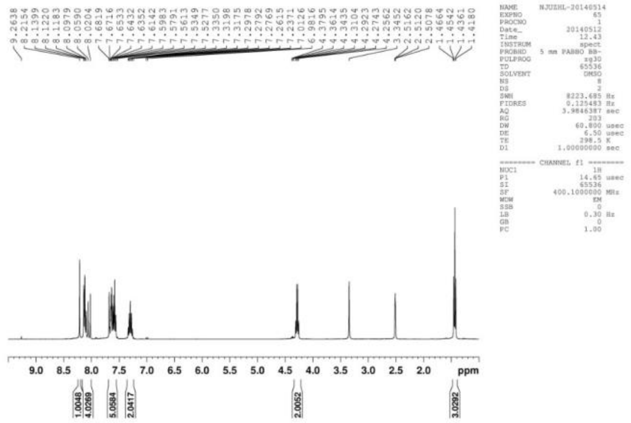

^{1}H-NMR of Compound of **23c**

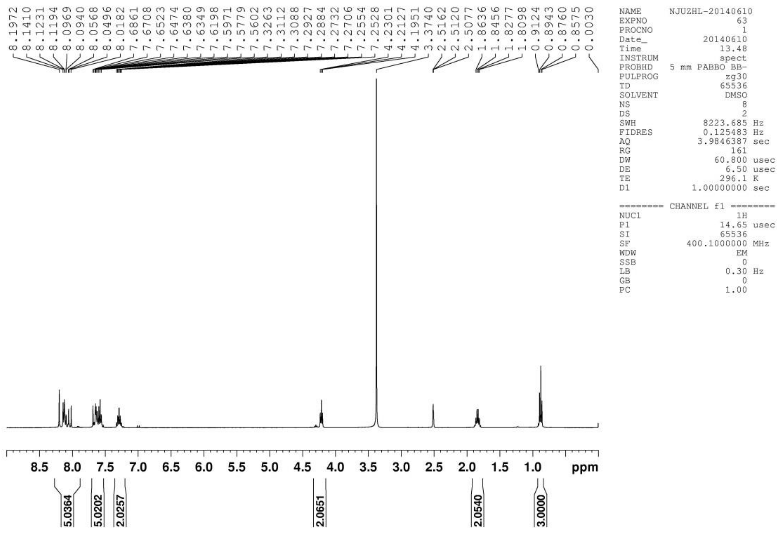

^{1}H-NMR of Compound of **23d**

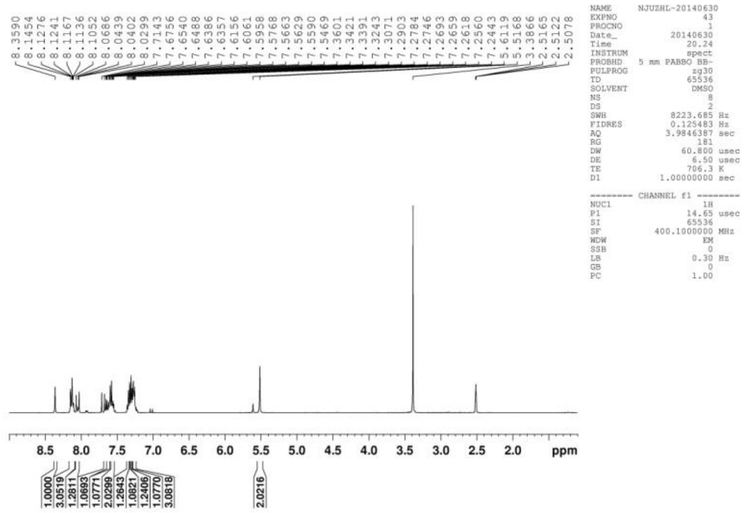

^{1}H-NMR of Compound of **23e**

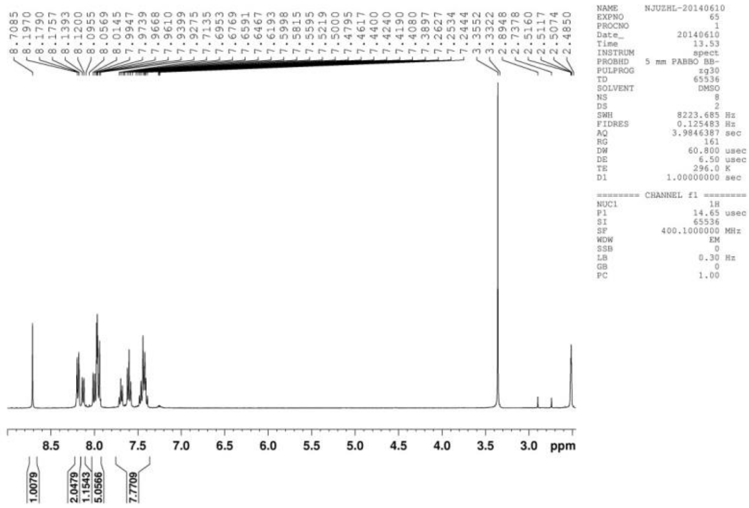

^{1}H-NMR of Compound of **24a**

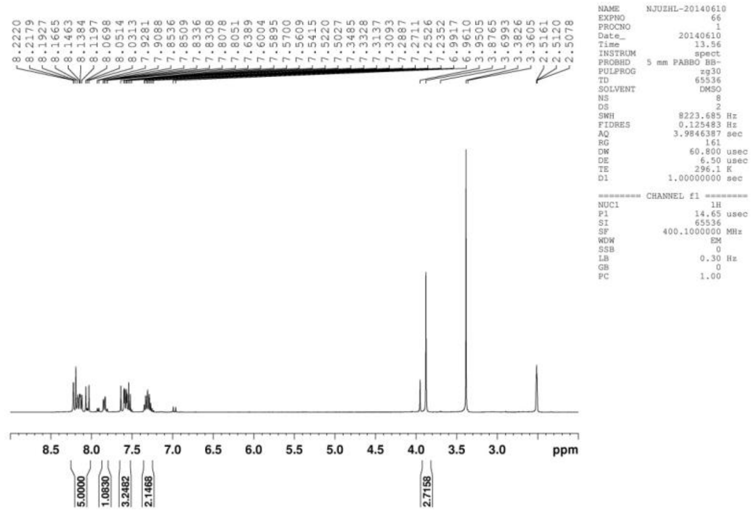

^{1}H-NMR of Compound of **24b**

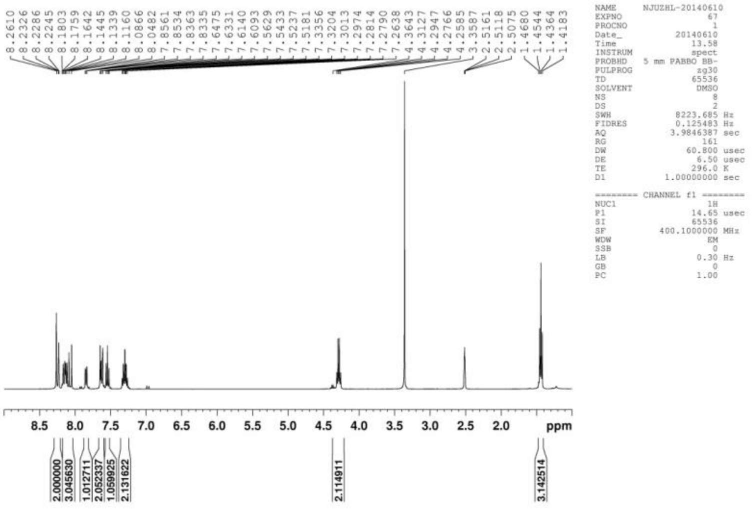

^{1}H-NMR of Compound of **24c**

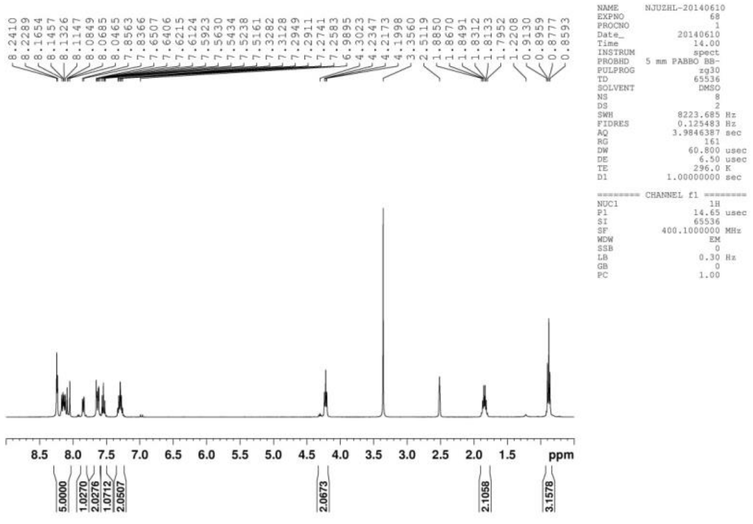

^{1}H-NMR of Compound of **24d**

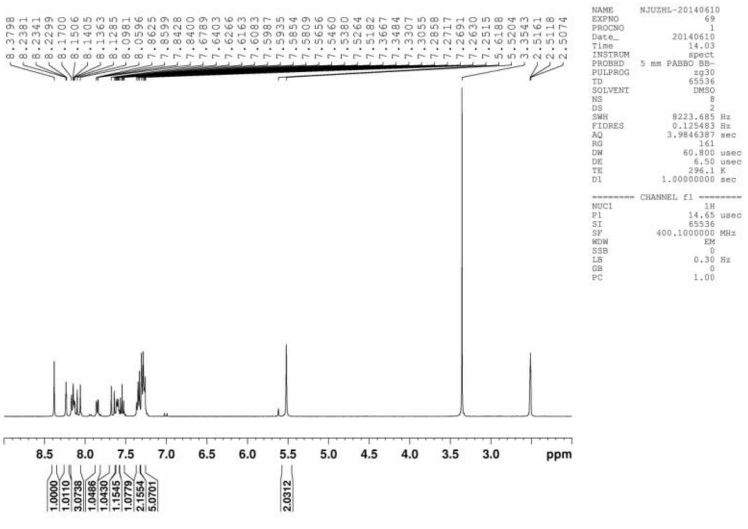

^{1}H-NMR of Compound of **24e**

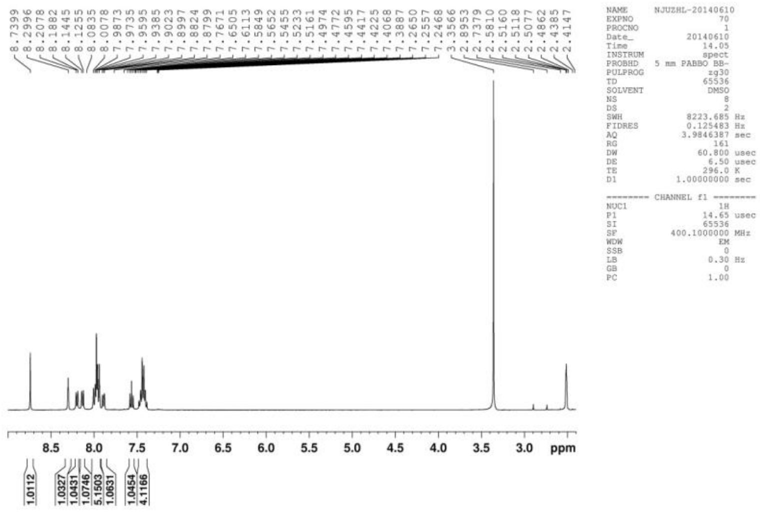

^{1}H-NMR of Compound of **25a**

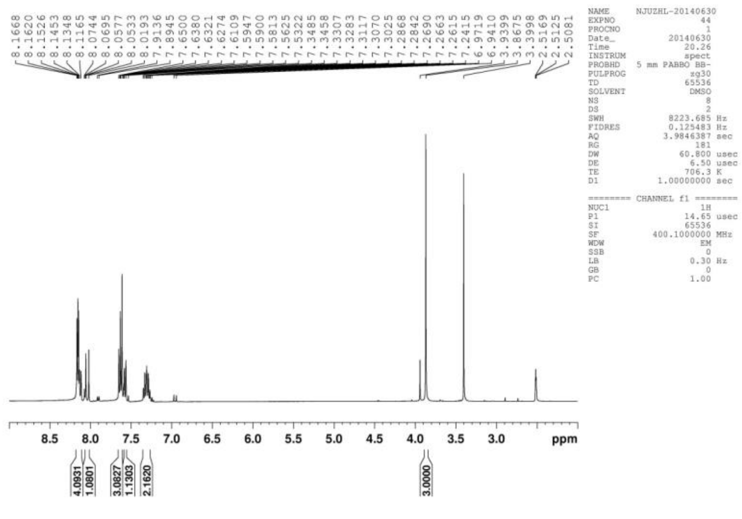

^{1}H-NMR of Compound of **25b**

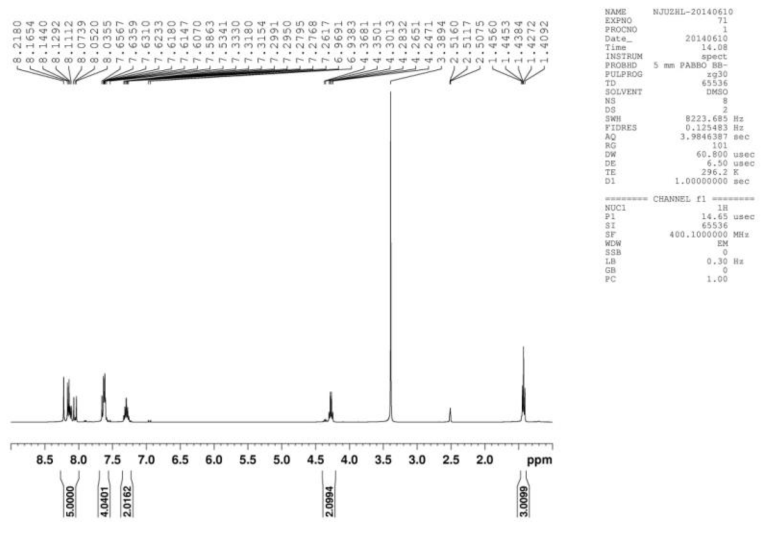

^{1}H-NMR of Compound of **25c**

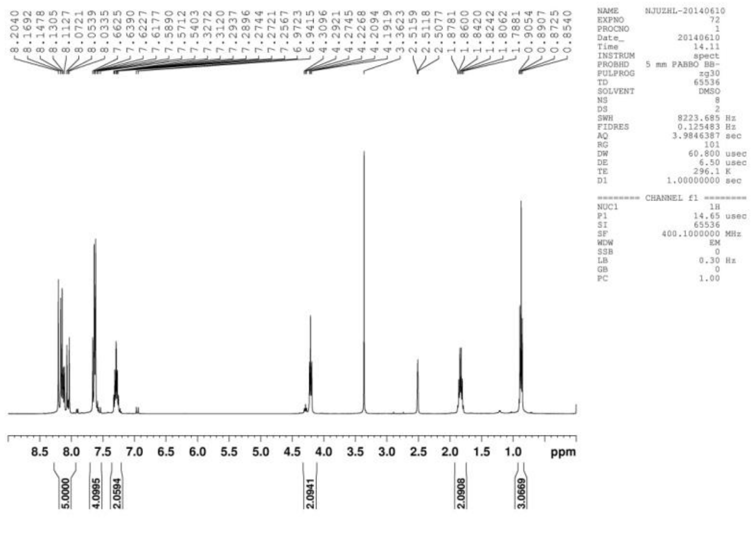

^{1}H-NMR of Compound of **25d**

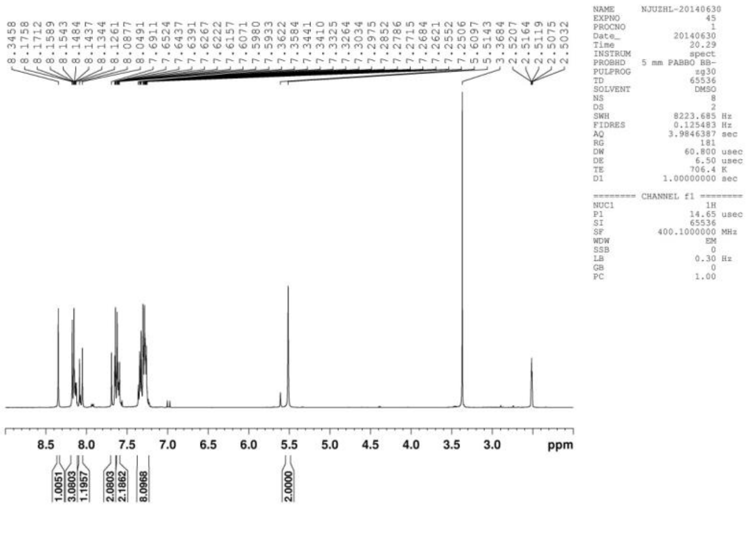

^{1}H-NMR of Compound of **25e**

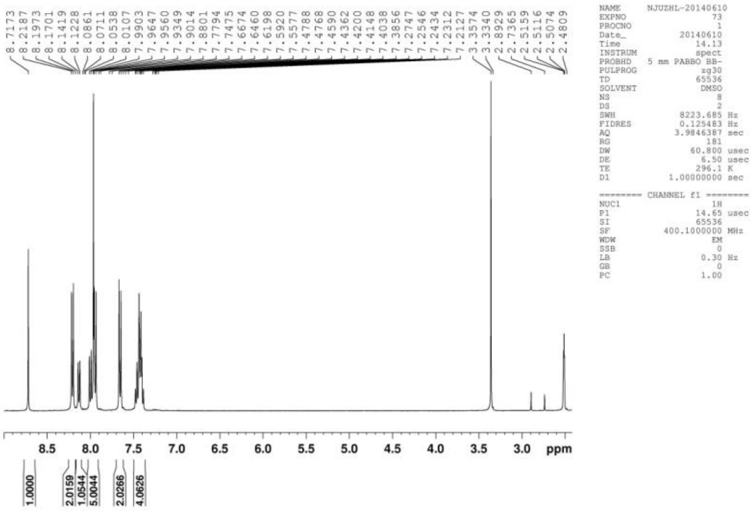

^{1}H-NMR of Compound of **26a**

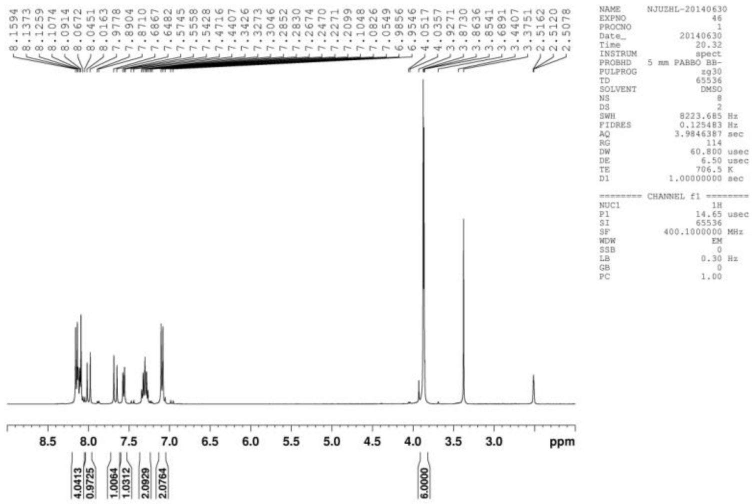

^{1}H-NMR of Compound of **26b**

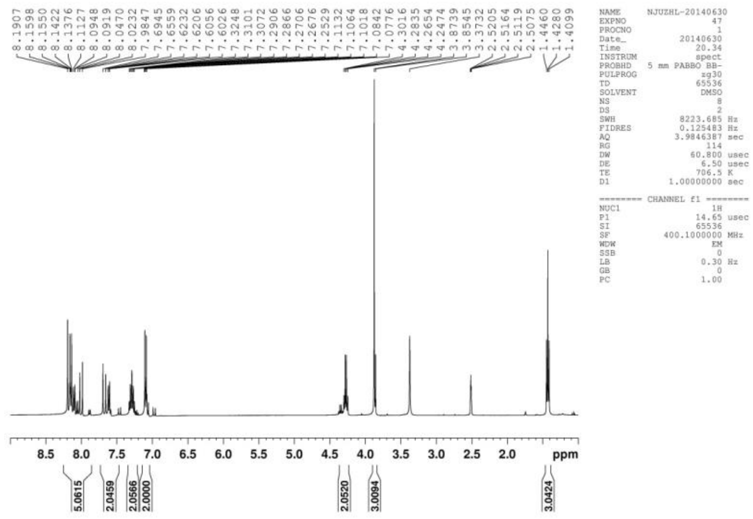

^{1}H-NMR of Compound of **26c**

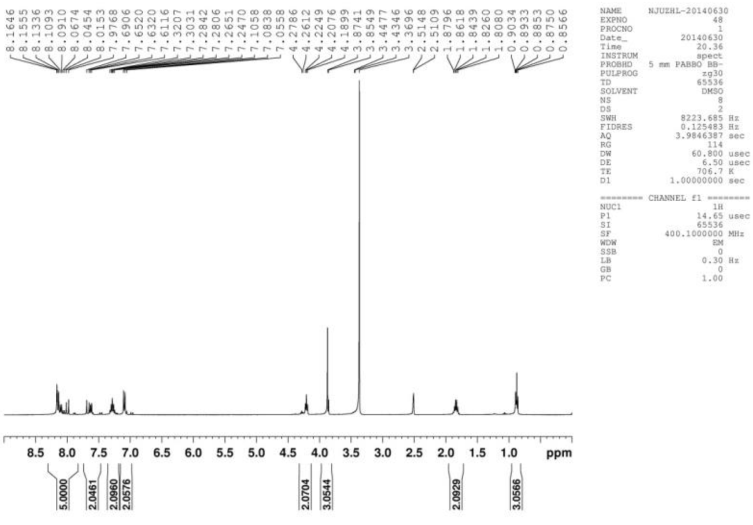

^{1}H-NMR of Compound of **26d**

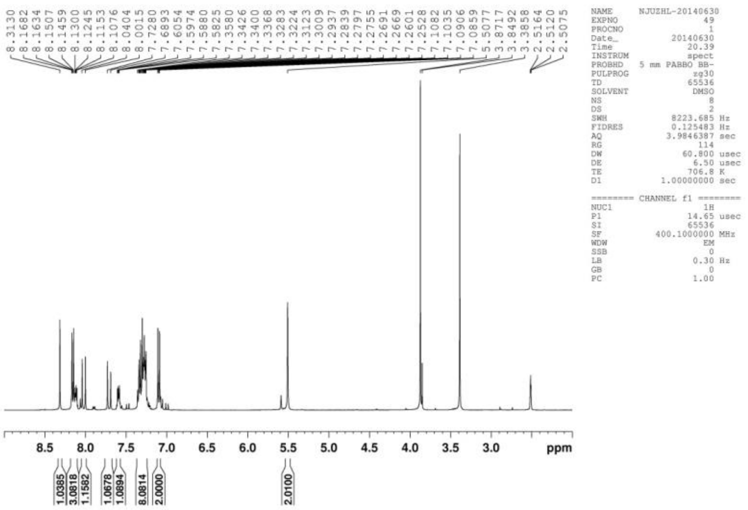

^{1}H-NMR of Compound of **26e**

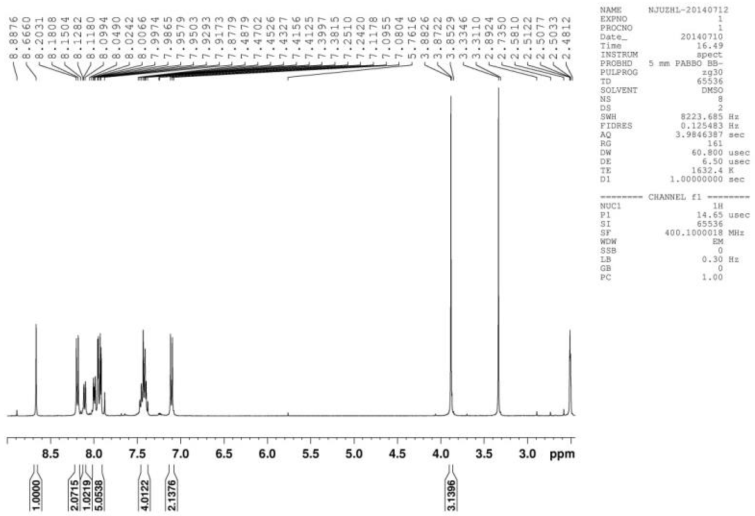

^{1}H-NMR of Compound of **27a**

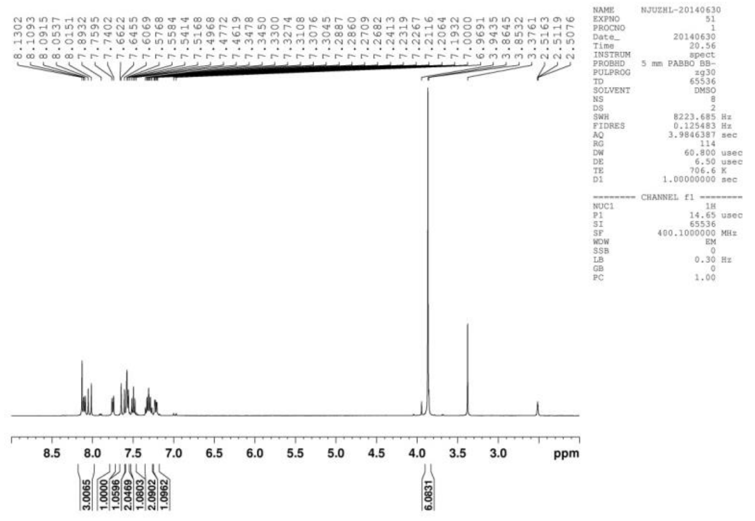

^{1}H-NMR of Compound of **27b**

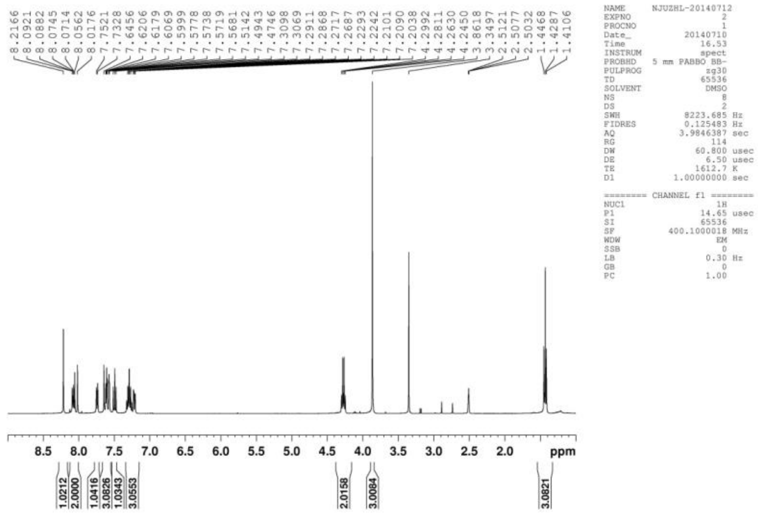

^{1}H-NMR of Compound of **27c**

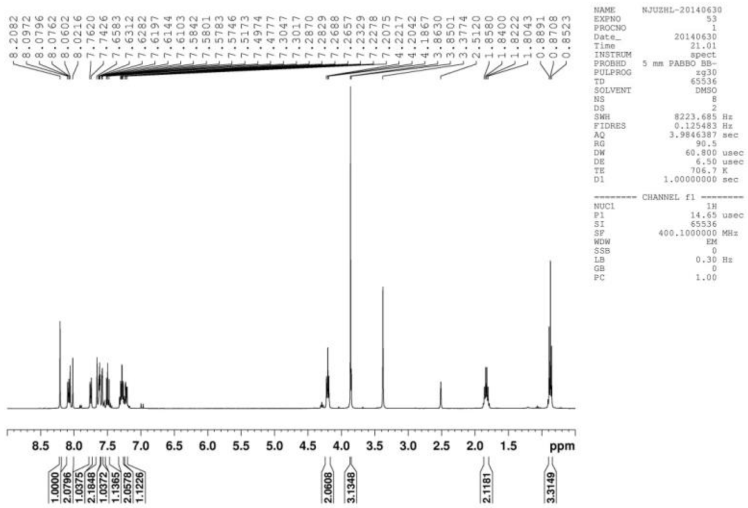

^{1}H-NMR of Compound of **27d**

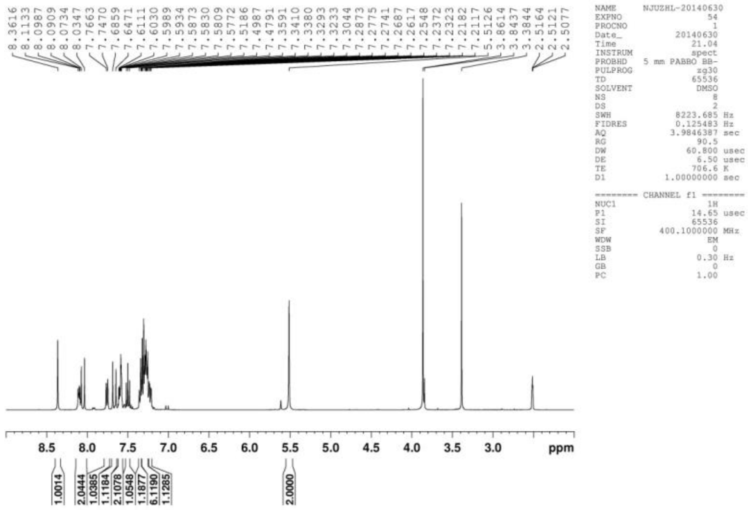

^{1}H-NMR of Compound of **27e**

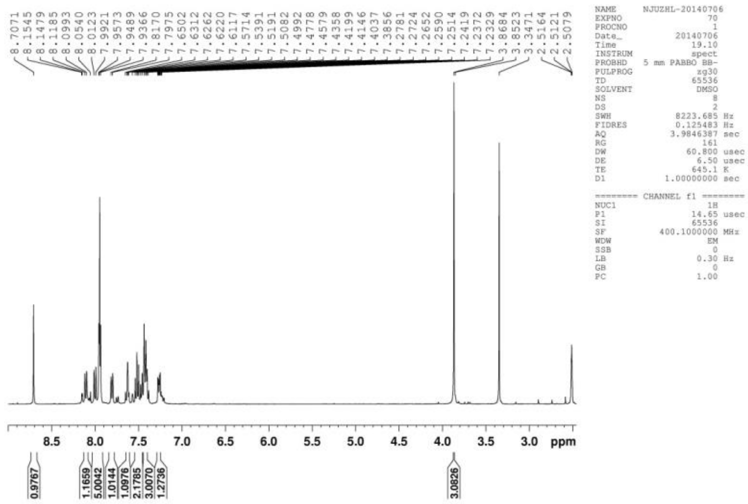

^{1}H-NMR of Compound of **28a**

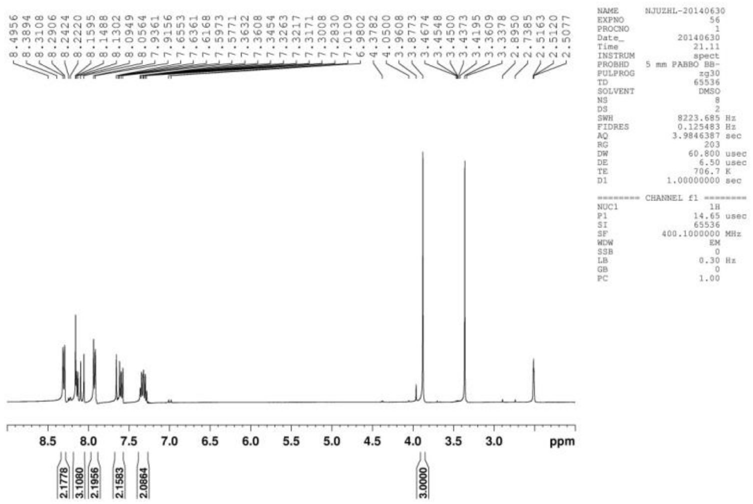

^{1}H-NMR of Compound of **28b**

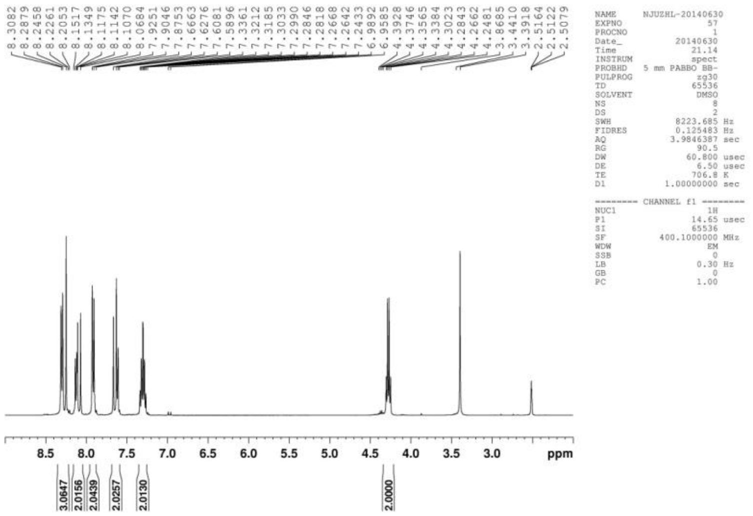

^{1}H-NMR of Compound of **28c**

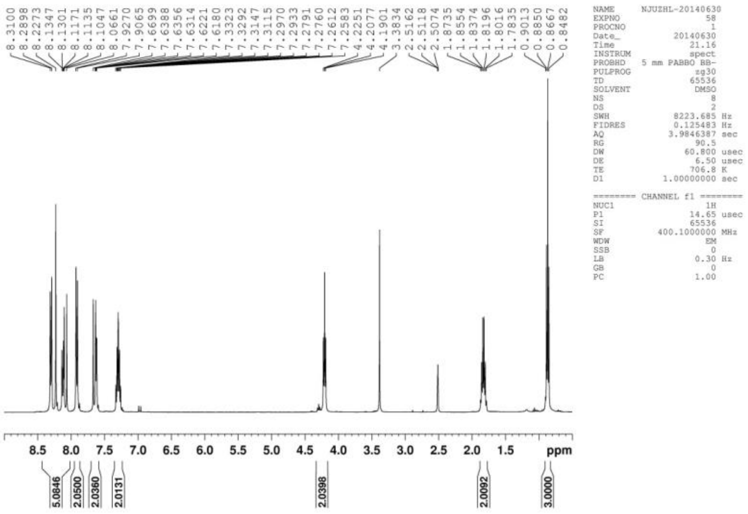

^{1}H-NMR of Compound of **28d**

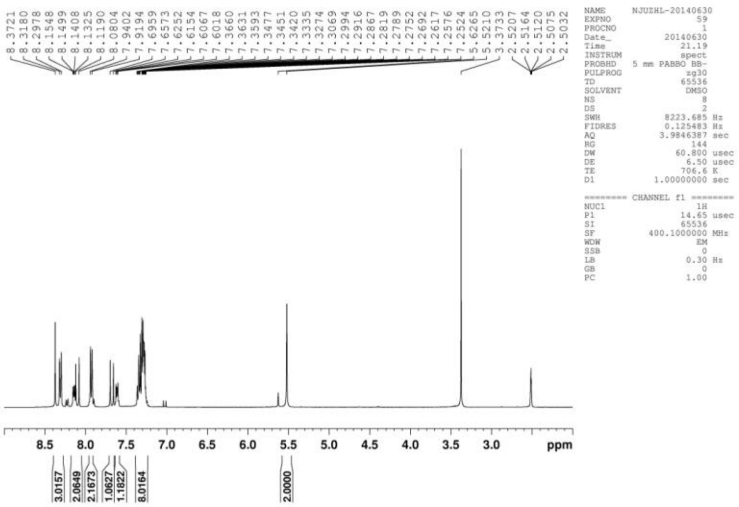

^{1}H-NMR of Compound of **28e**

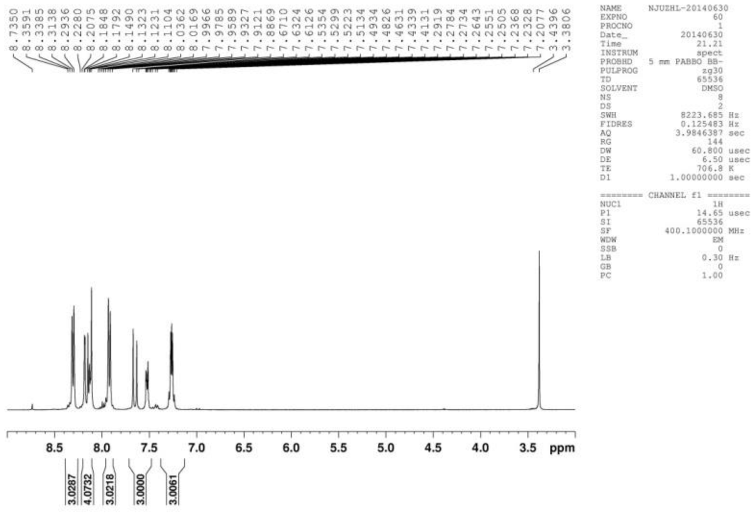

^{1}H-NMR of Compound of **29a**

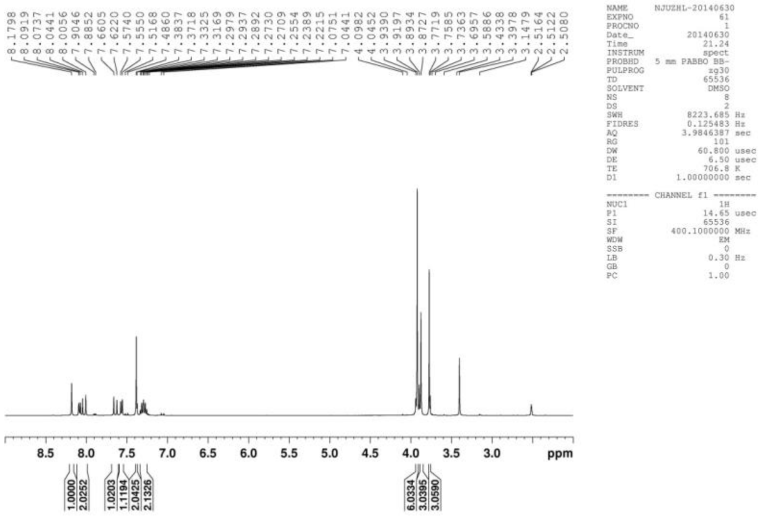

^{1}H-NMR of Compound of **29b**

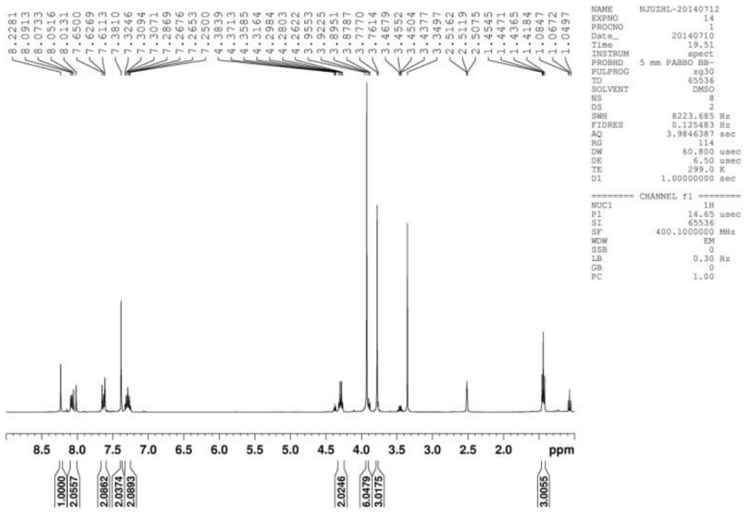

^{1}H-NMR of Compound of **29c**

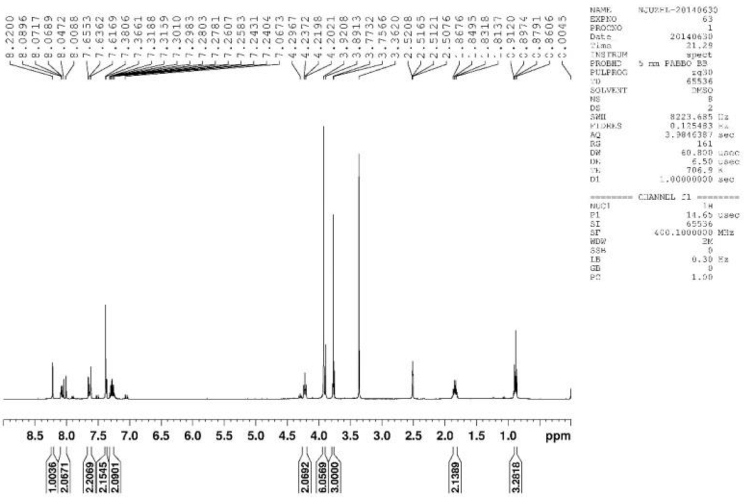

^{1}H-NMR of Compound of **29d**

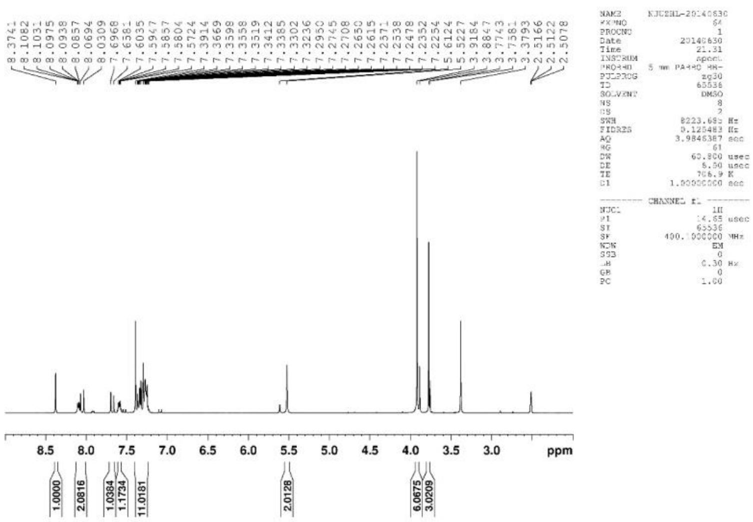

^{1}H-NMR of Compound of **29e**

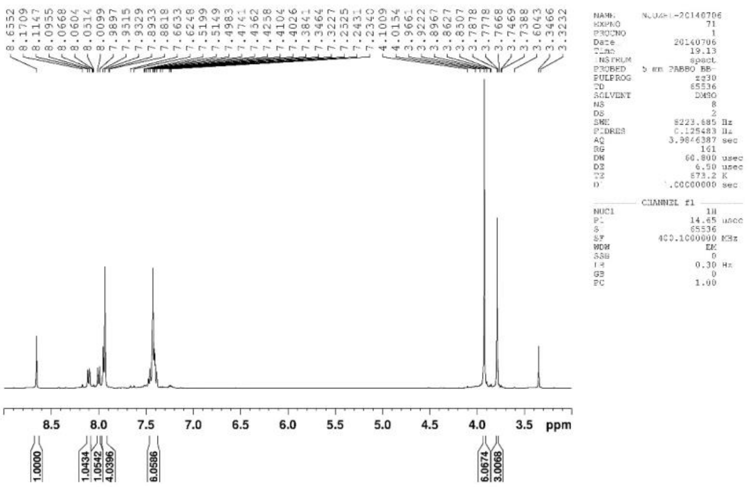

3. ^{13}C NMR of the representative compounds ^{13}C NMR of the representative

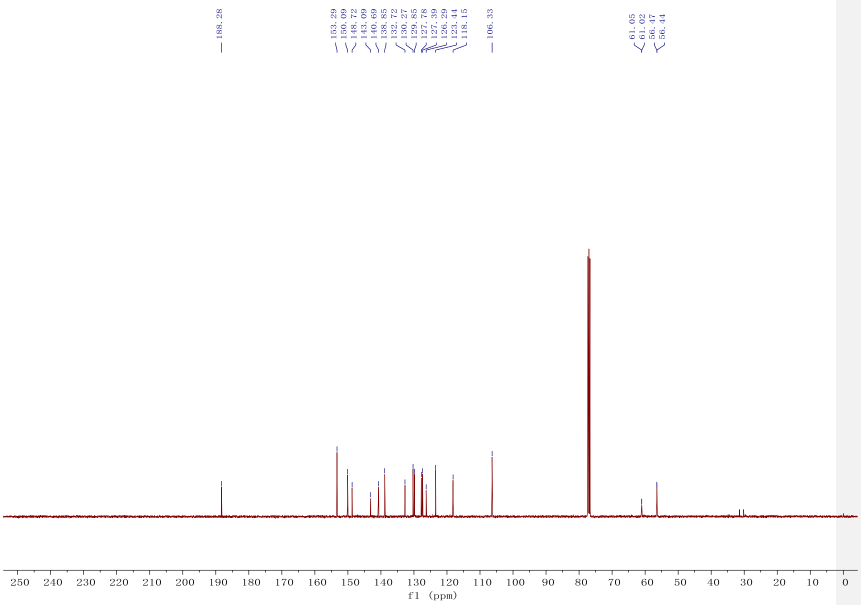

~~4.~~ ^{13}C NMR of Compound **DYT-1**

Formatted: Indent: Left: 0.63 cm, No bullets or numbering

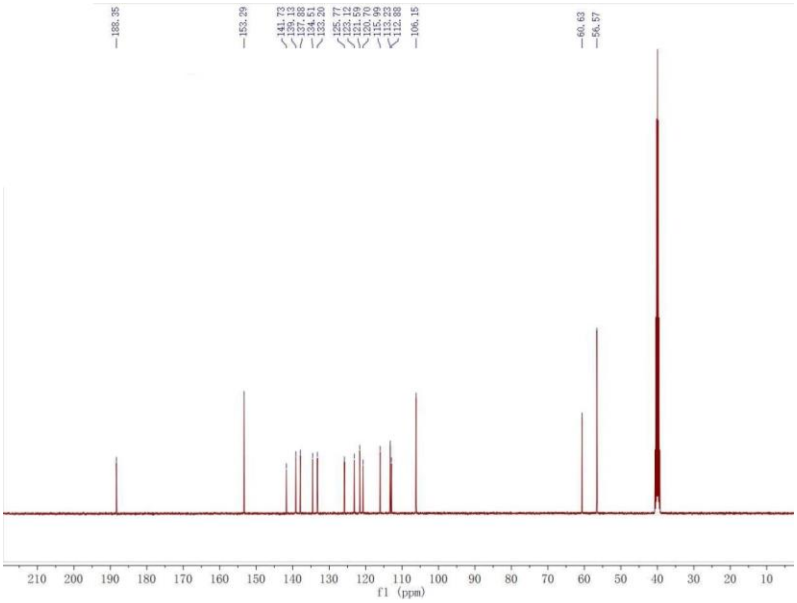

^{13}C NMR of Compound 6

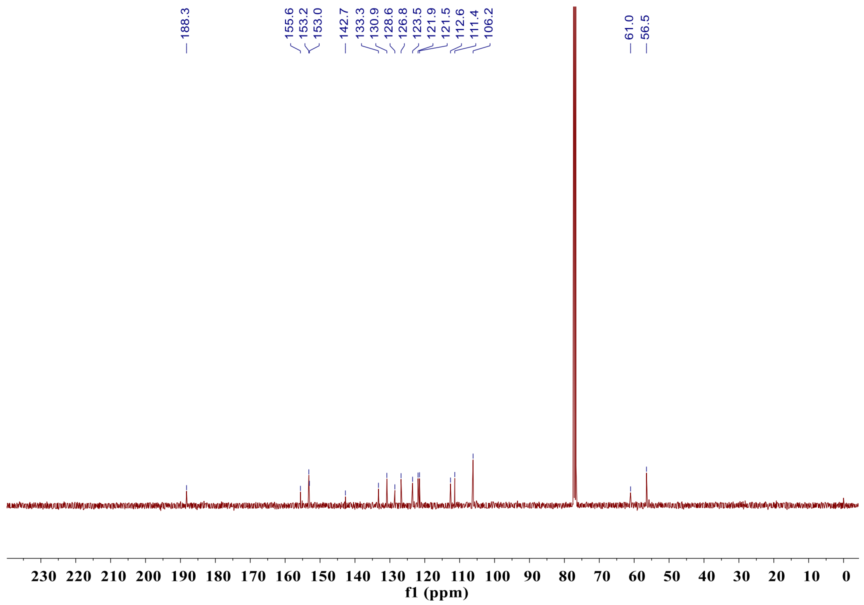

^{13}C NMR of Compound 7

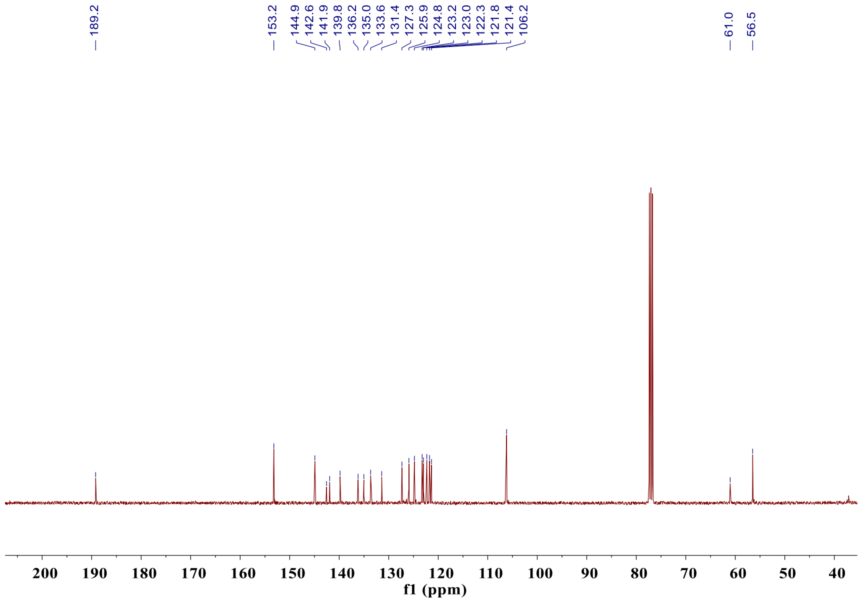

^{13}C NMR of Compound 8

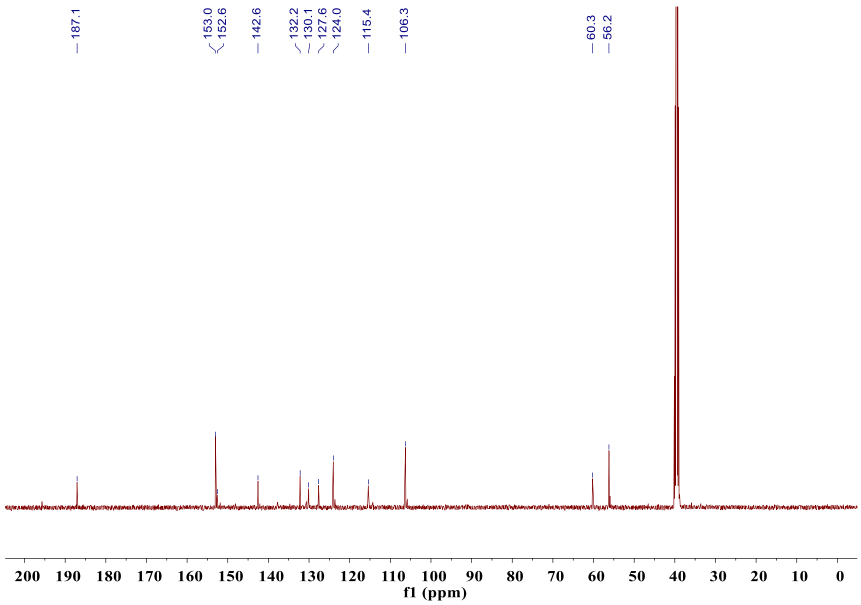

^{13}C NMR of Compound of **9**

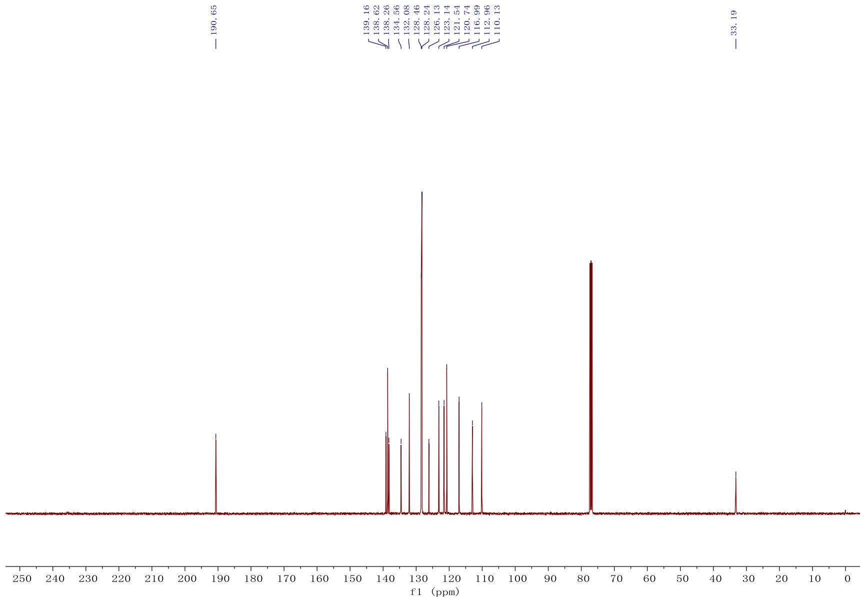

^{13}C NMR of Compound of **23a**

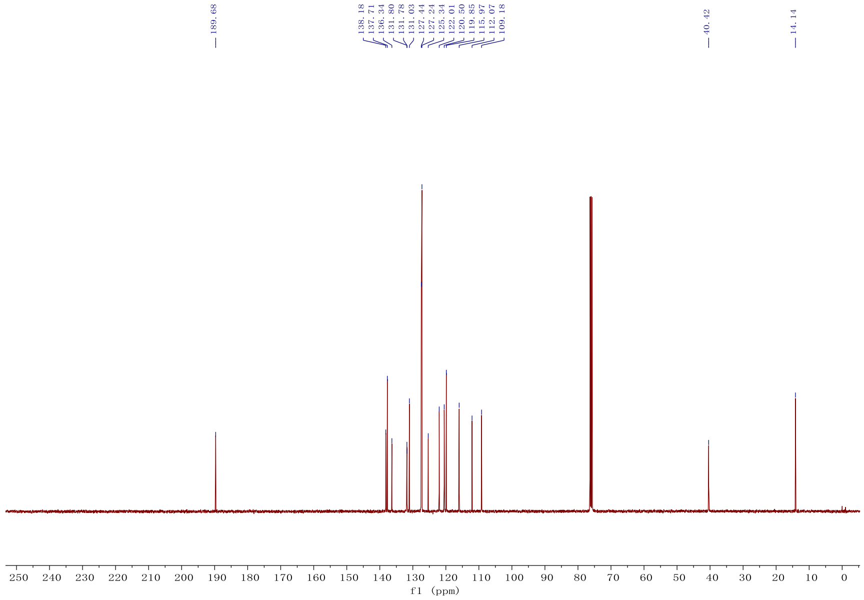

^{13}C NMR of Compound of **23b**

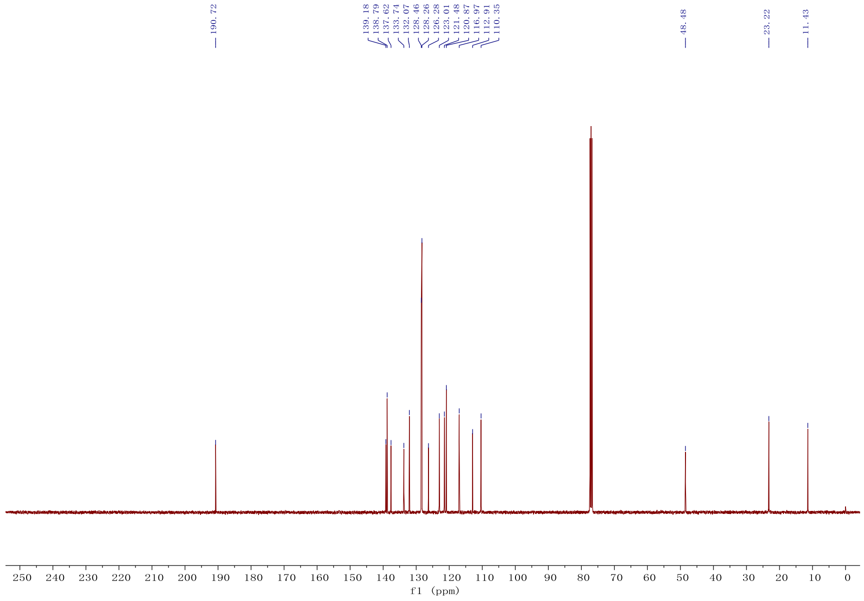

^{13}C NMR of Compound of **23c**

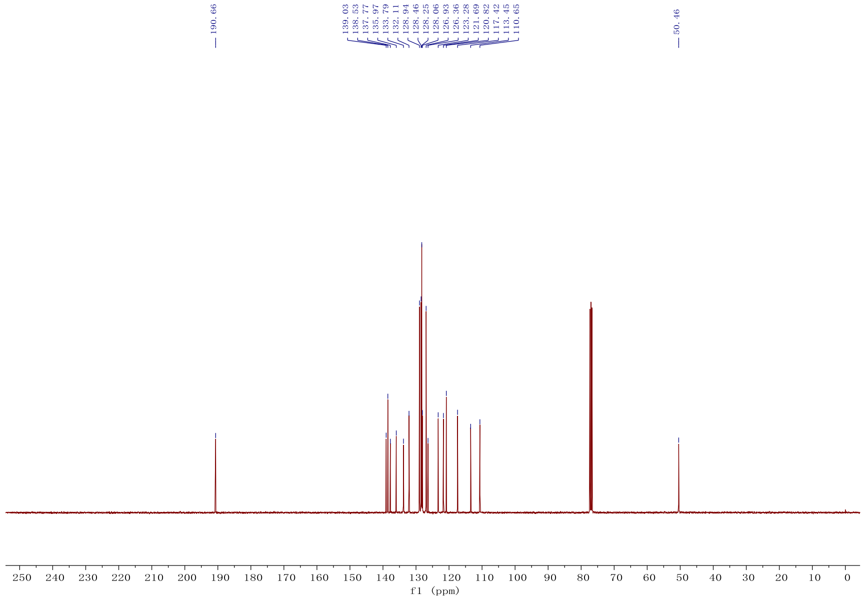

^{13}C NMR of Compound of **23d**

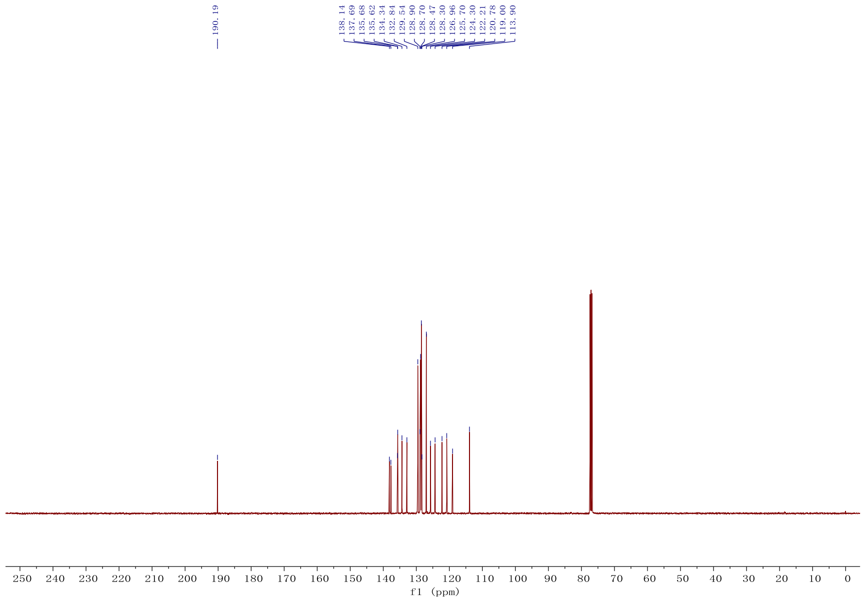

^{13}C NMR of Compound of **23e**

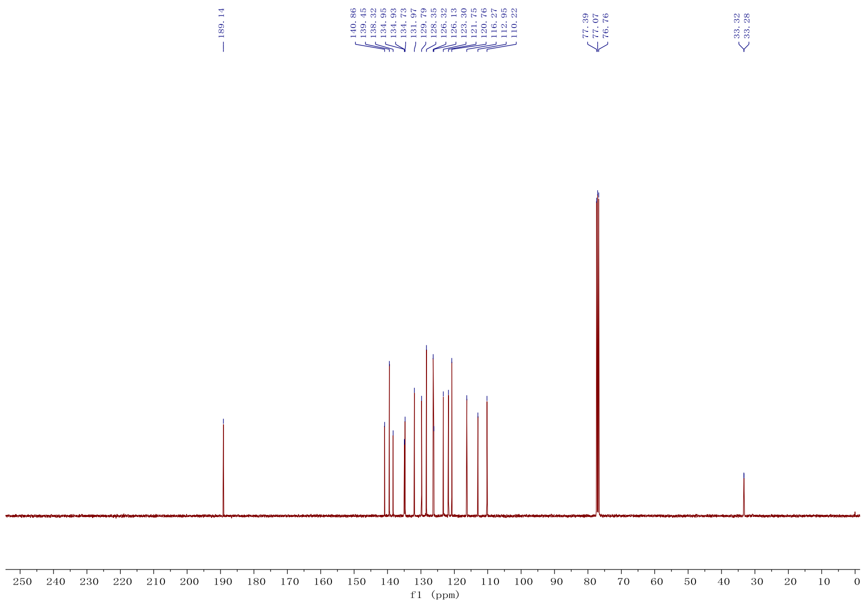

^{13}C NMR of Compound of **24a**

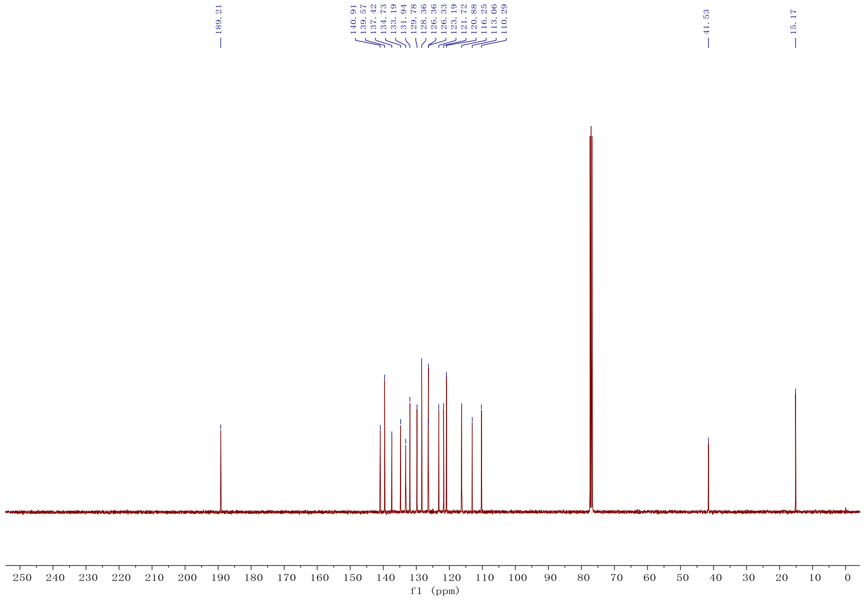

^{13}C NMR of Compound of **24b**

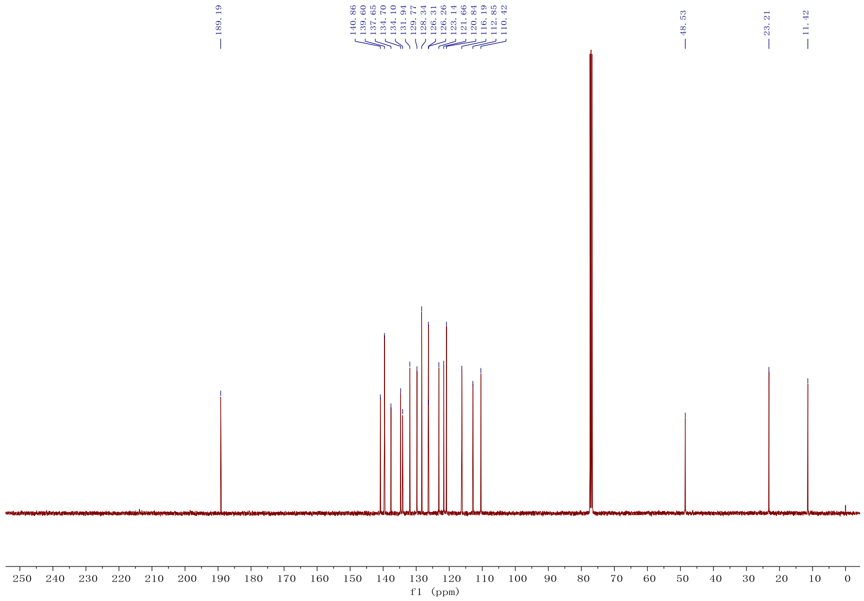

^{13}C NMR of Compound of **24c**

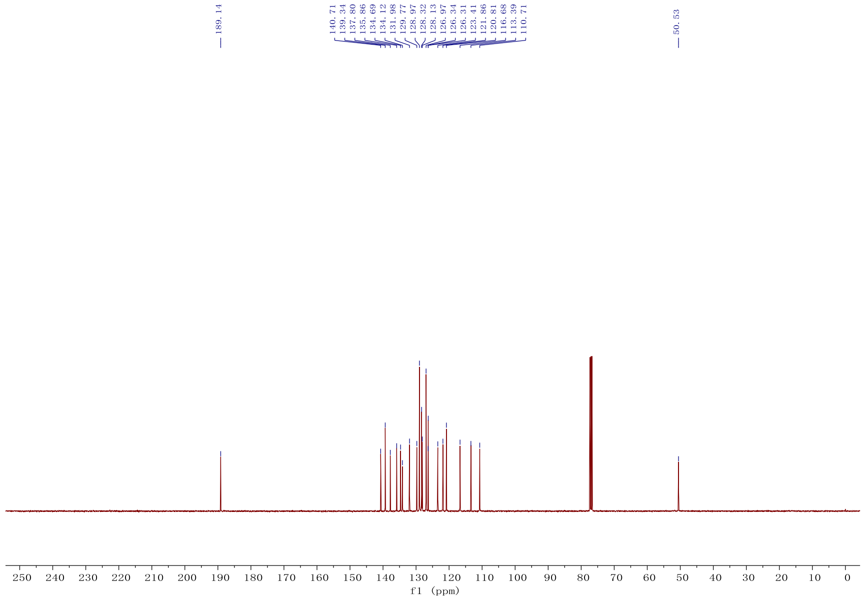

^{13}C NMR of Compound of **24d**

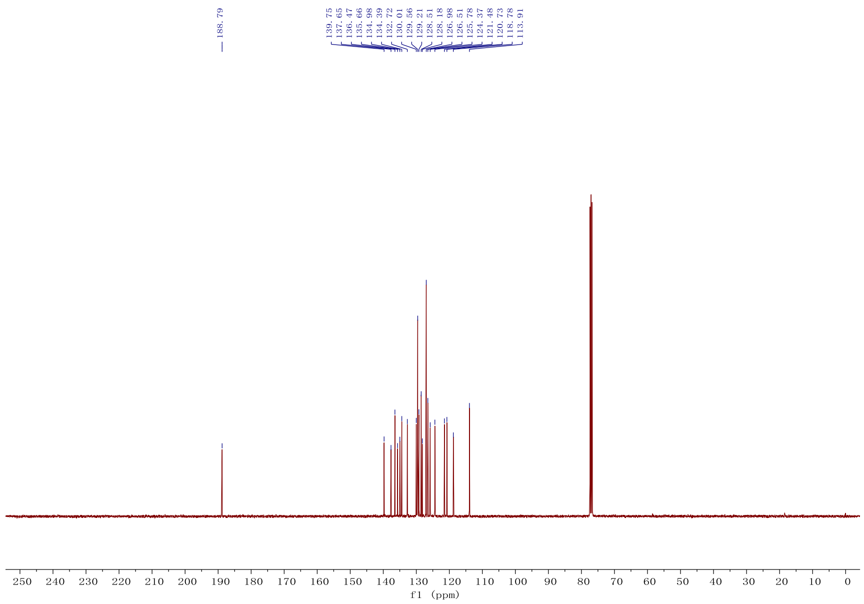

^{13}C NMR of Compound of **24e**

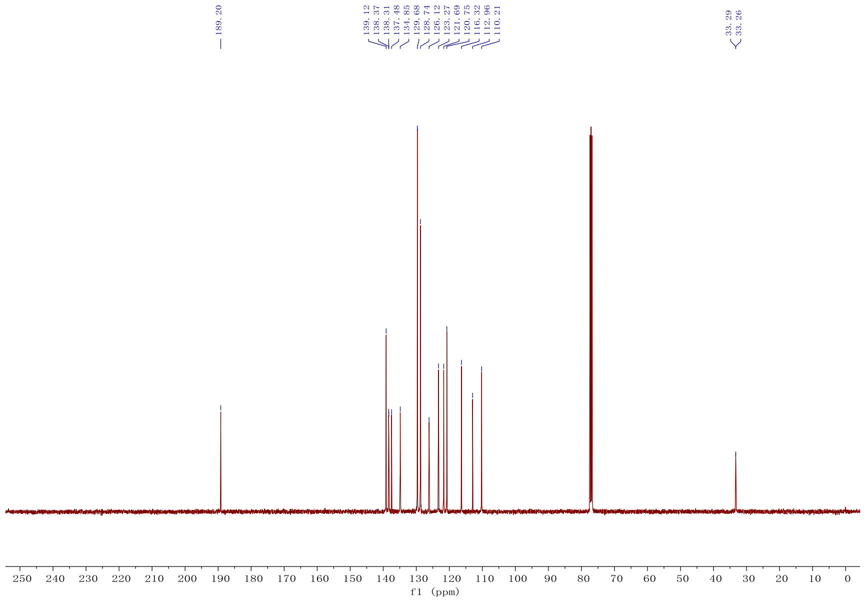

^{13}C NMR of Compound of **25a**

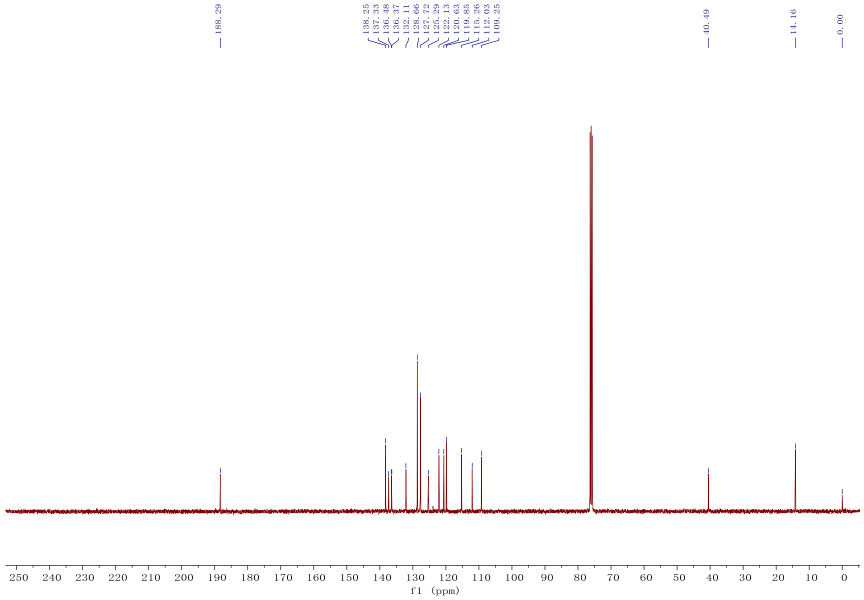

^{13}C NMR of Compound of **25b**

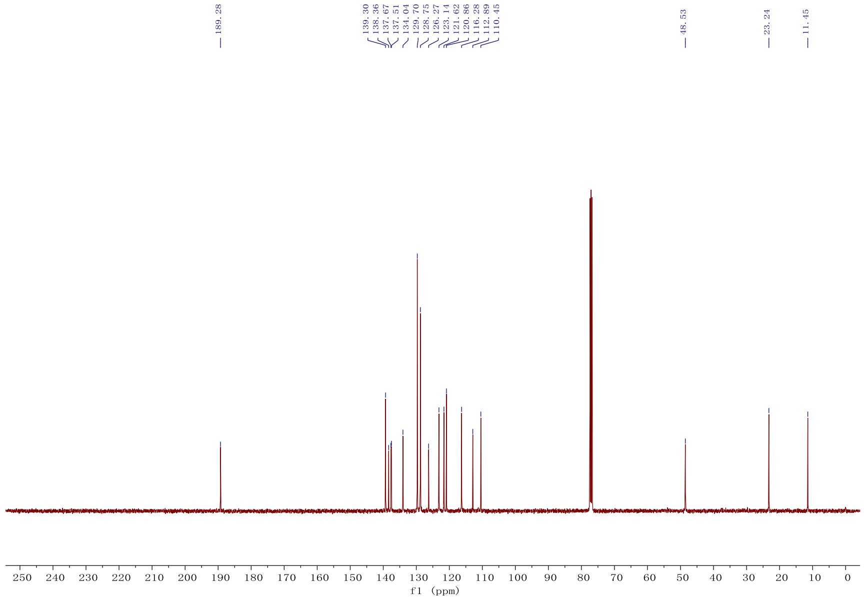

^{13}C NMR of Compound of **25c**

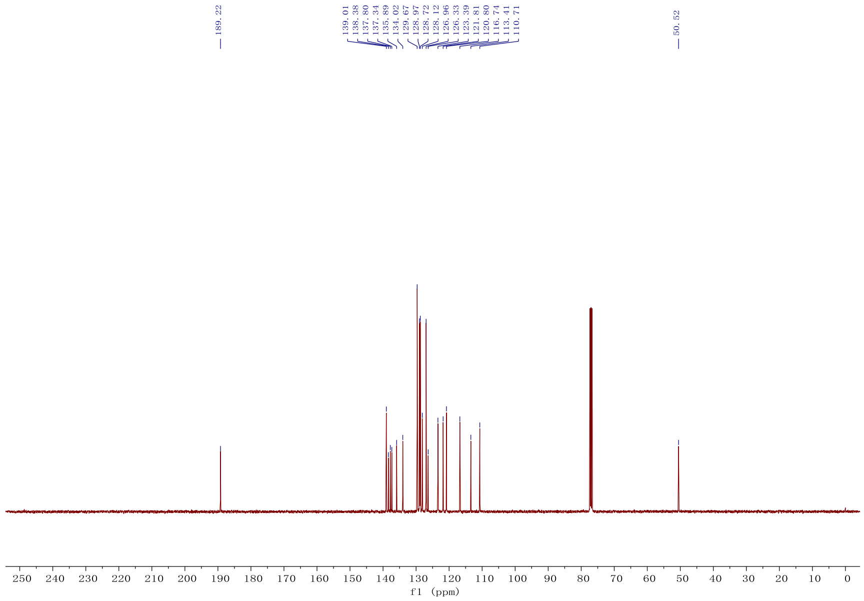

^{13}C NMR of Compound of **25d**

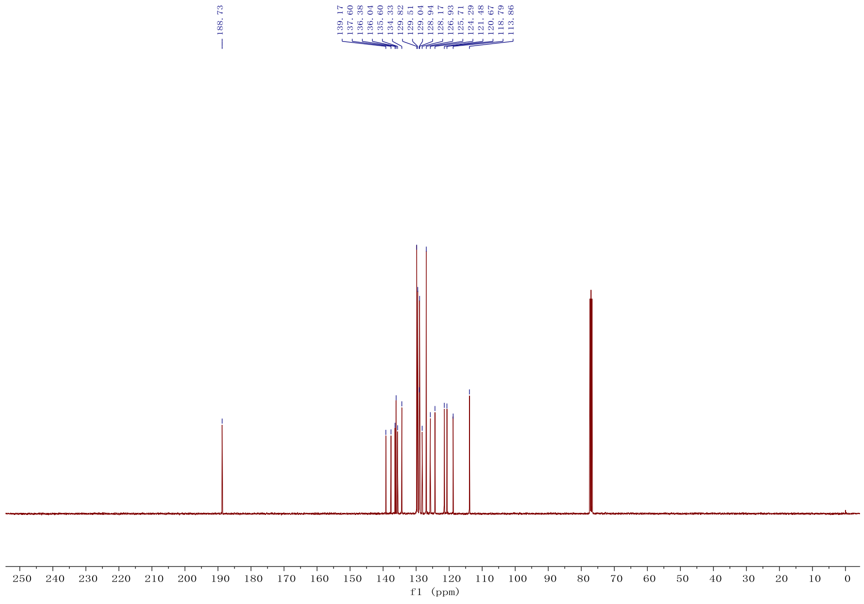

^{13}C NMR of Compound of **25e**

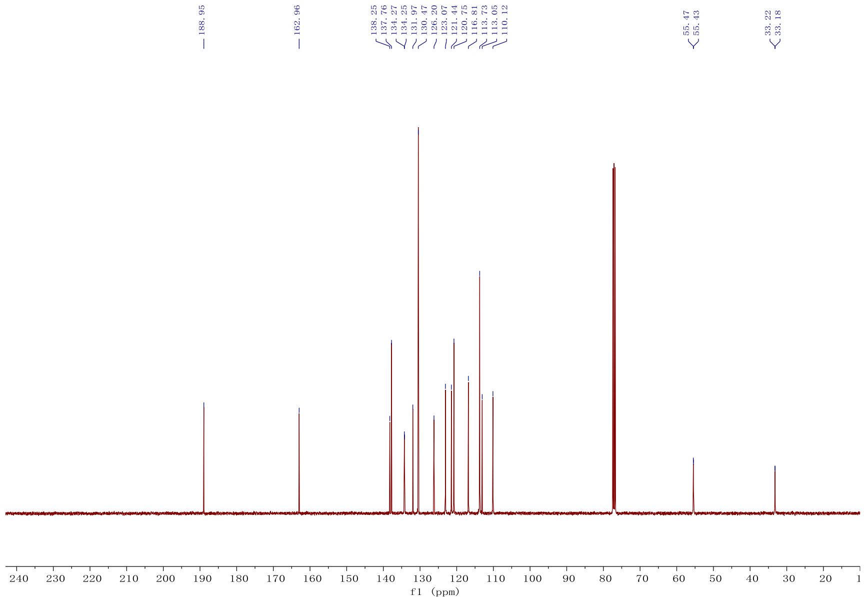

^{13}C NMR of Compound of **26a**

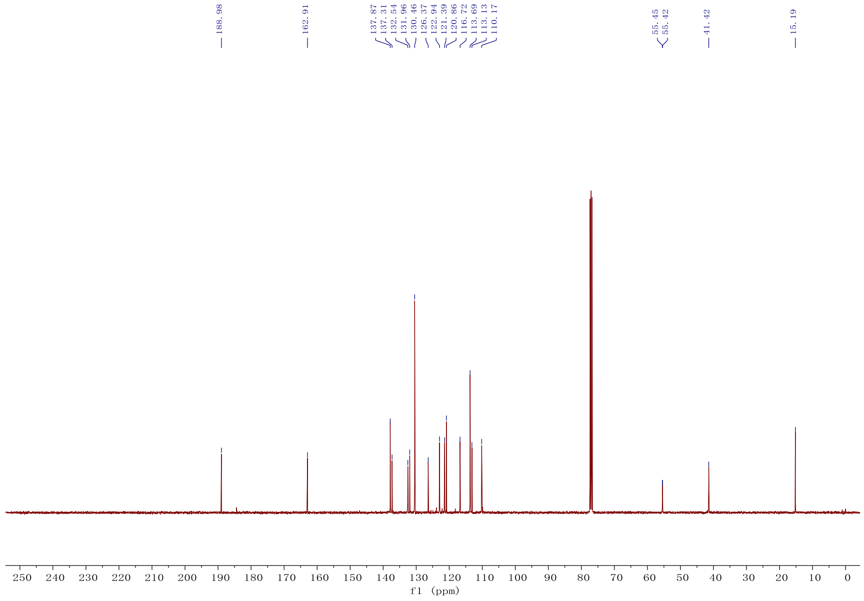

^{13}C NMR of Compound of **26b**

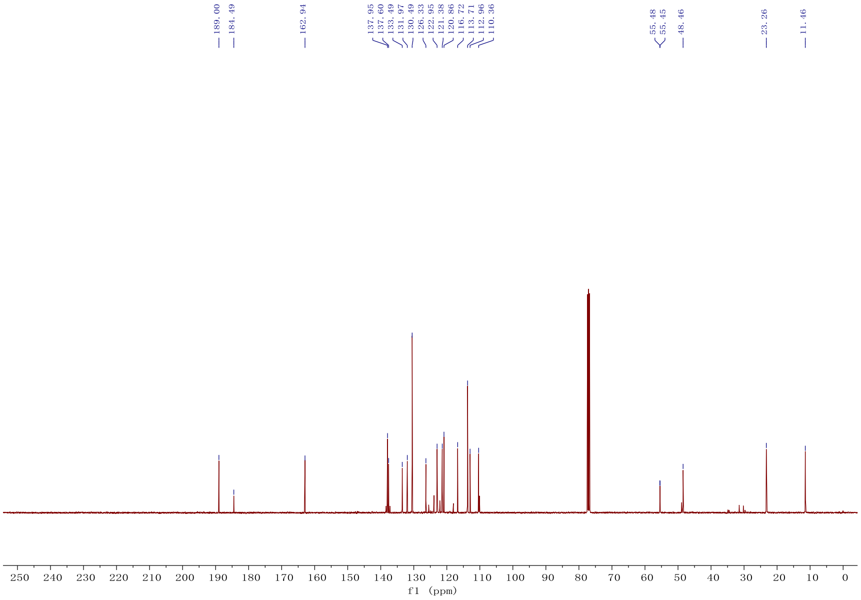

^{13}C NMR of Compound of **26c**

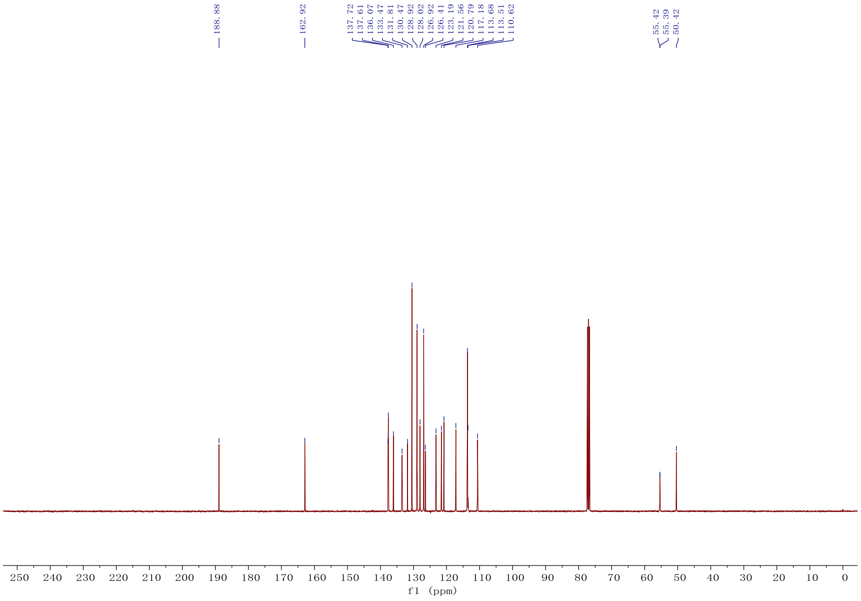

^{13}C NMR of Compound of **26d**

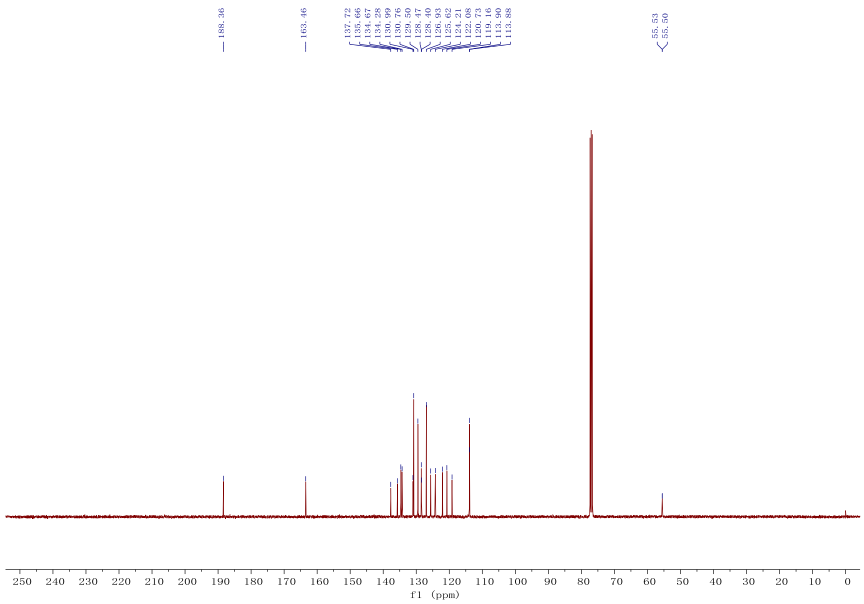

^{13}C NMR of Compound of **26e**

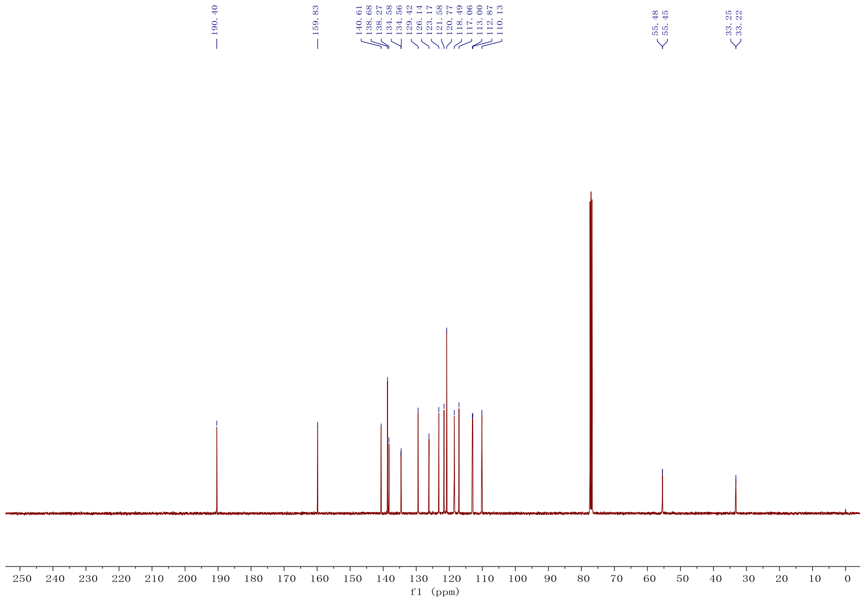

^{13}C NMR of Compound of **27a**

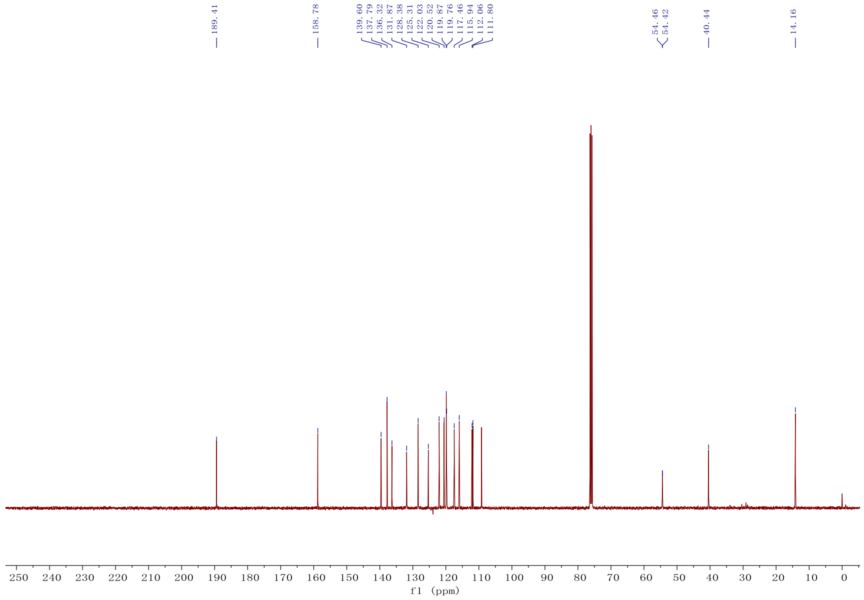

^{13}C NMR of Compound of **27b**

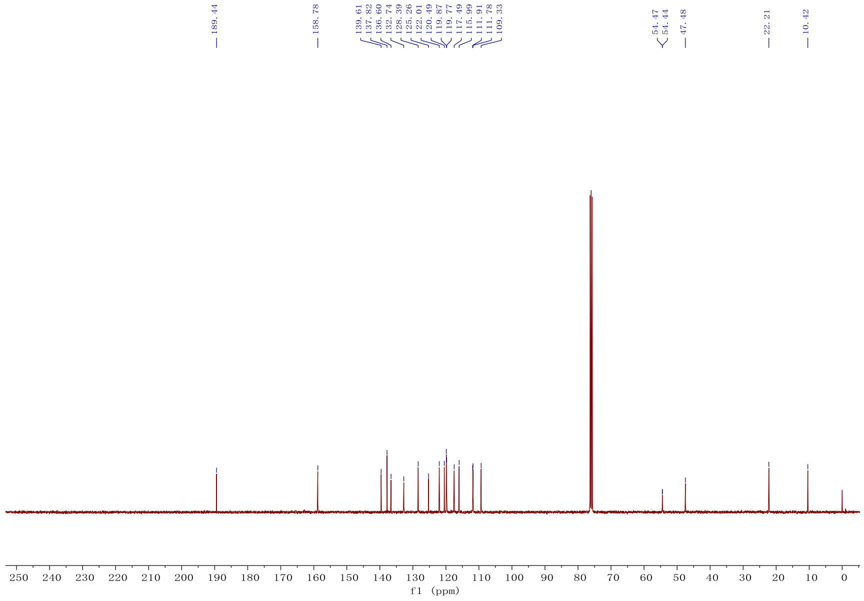

^{13}C NMR of Compound of **27c**

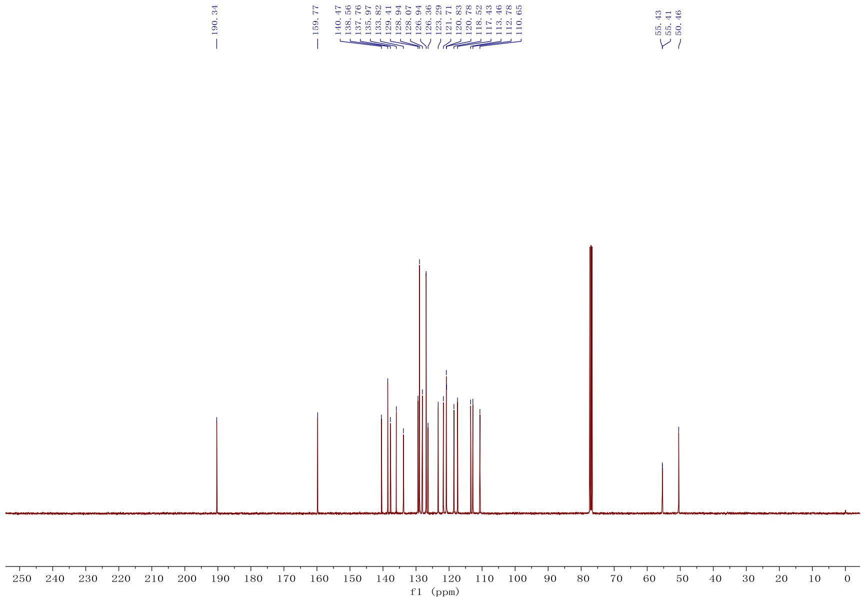

^{13}C NMR of Compound of **27d**

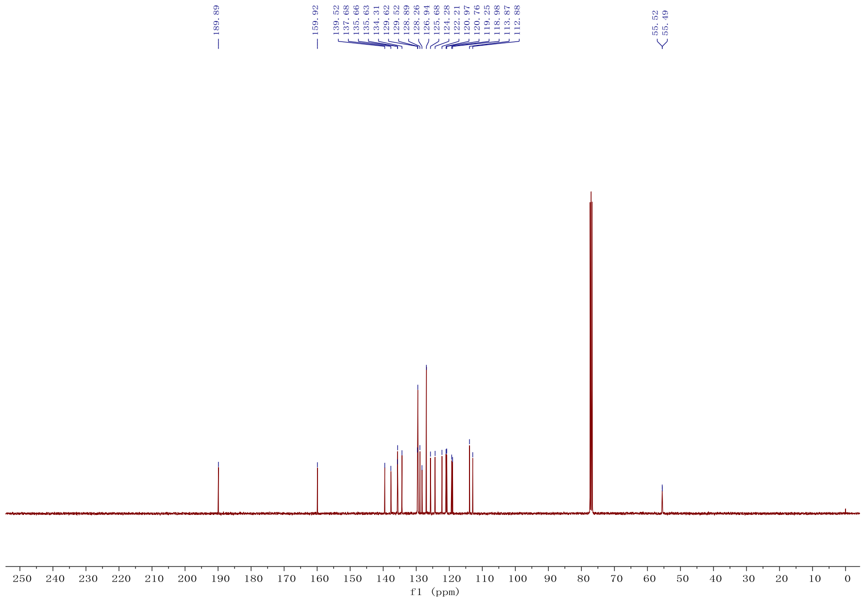

^{13}C NMR of Compound of **27e**

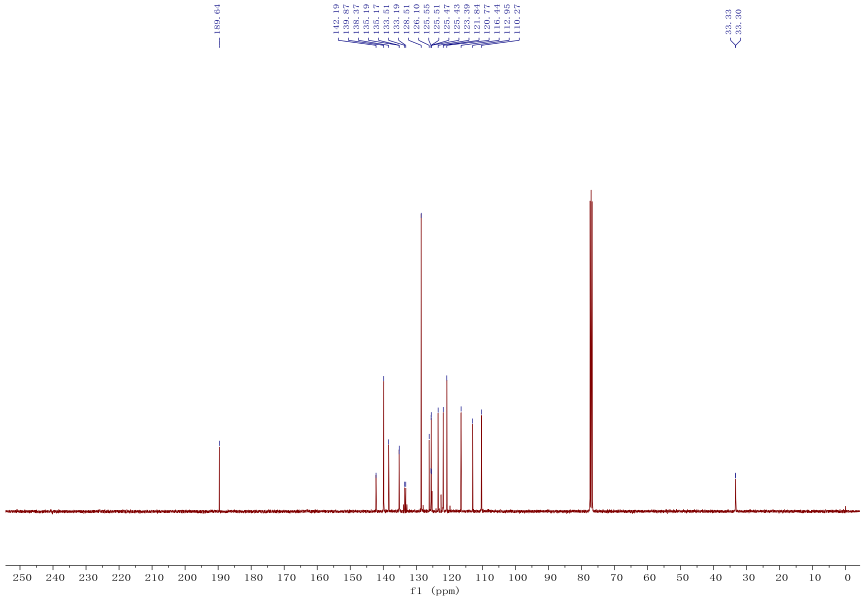

^{13}C NMR of Compound of **28a**

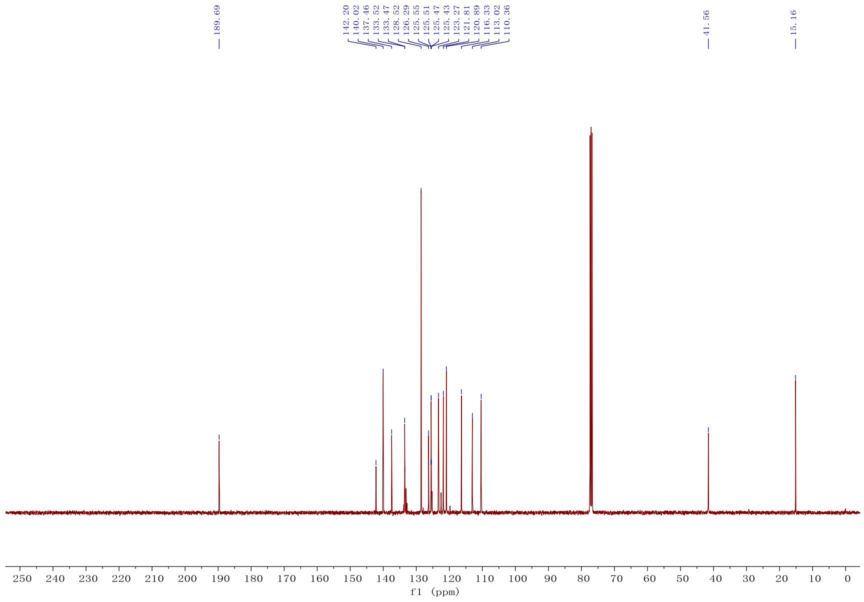

^{13}C NMR of Compound of **28b**

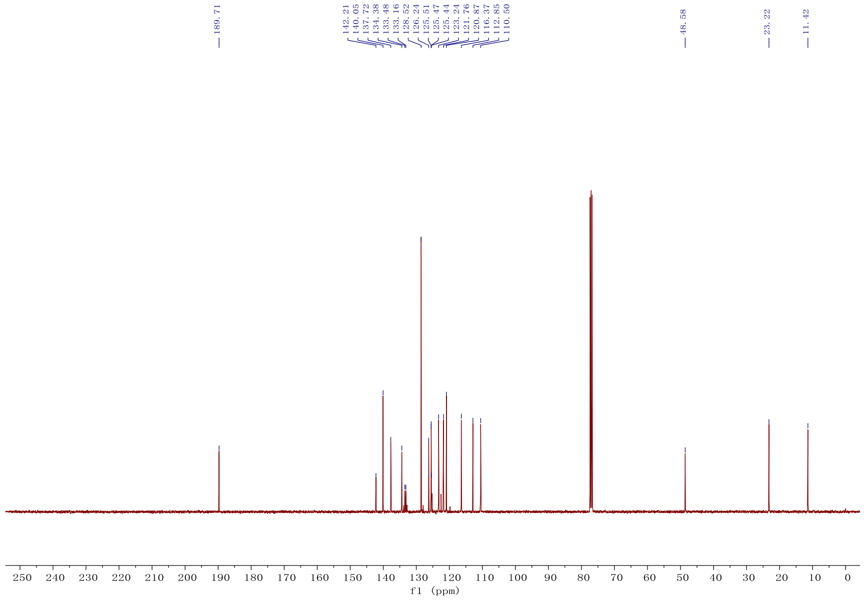

^{13}C NMR of Compound of **28c**

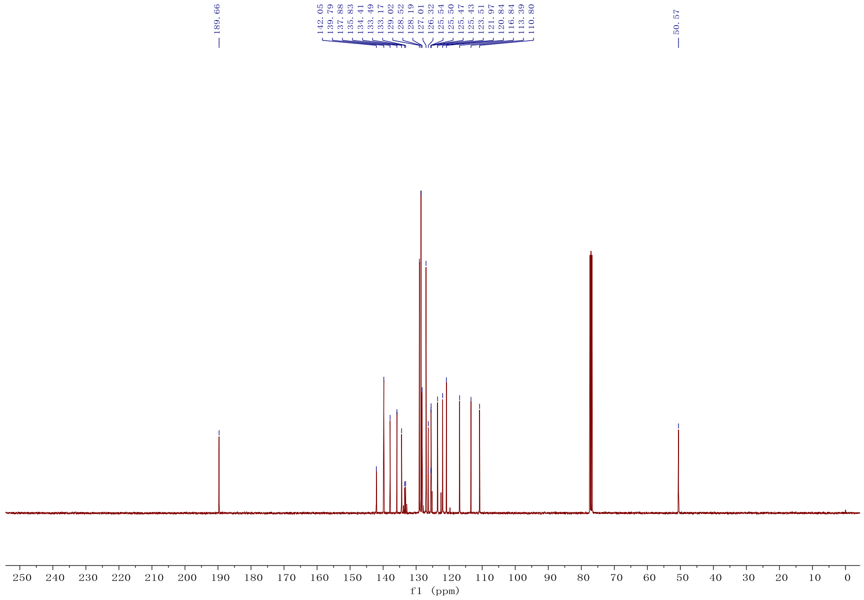

^{13}C NMR of Compound of **28d**

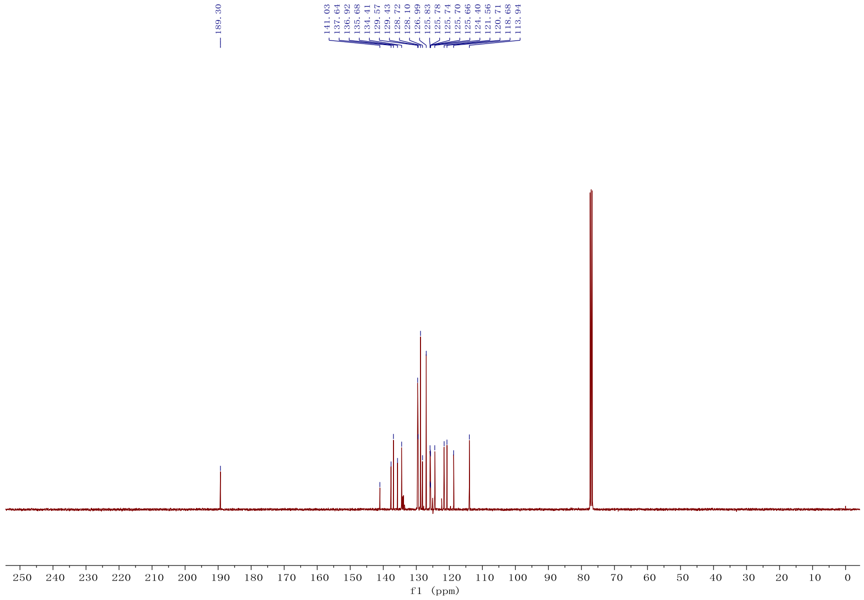

^{13}C NMR of Compound of **28e**

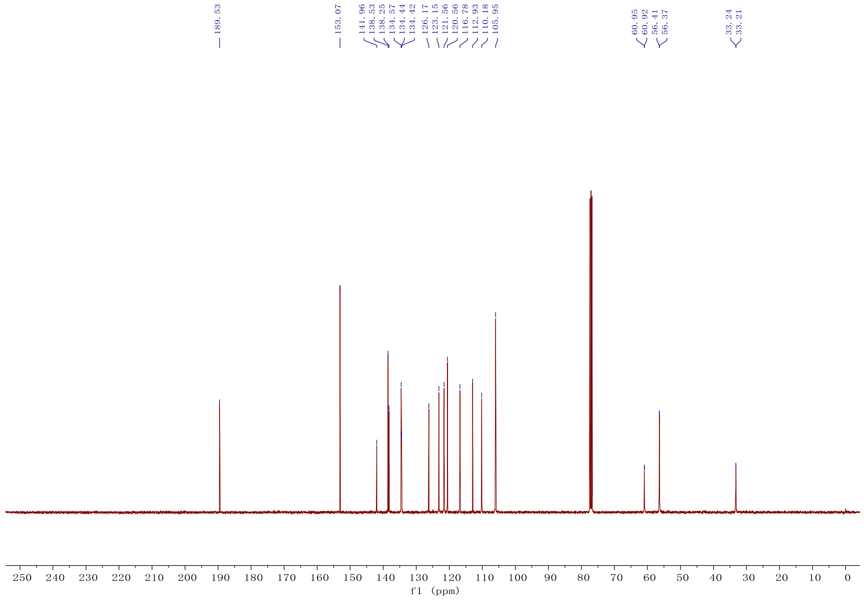

^{13}C NMR of Compound of **29a**

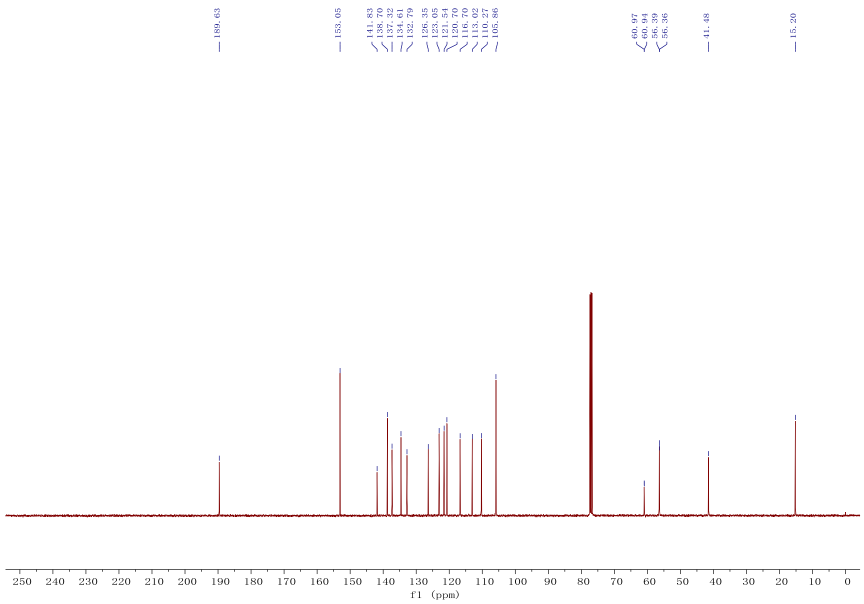

^{13}C NMR of Compound of **29b**

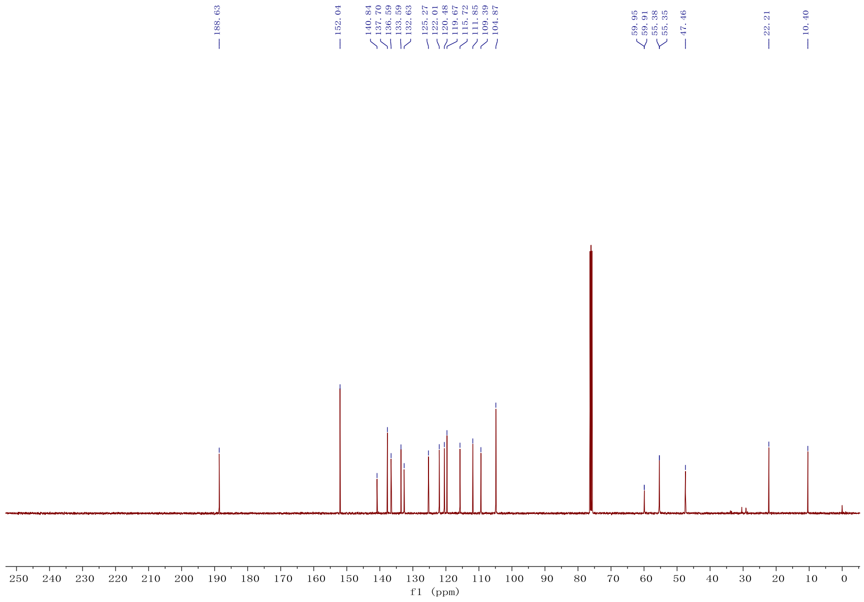

^{13}C NMR of Compound of **29c**

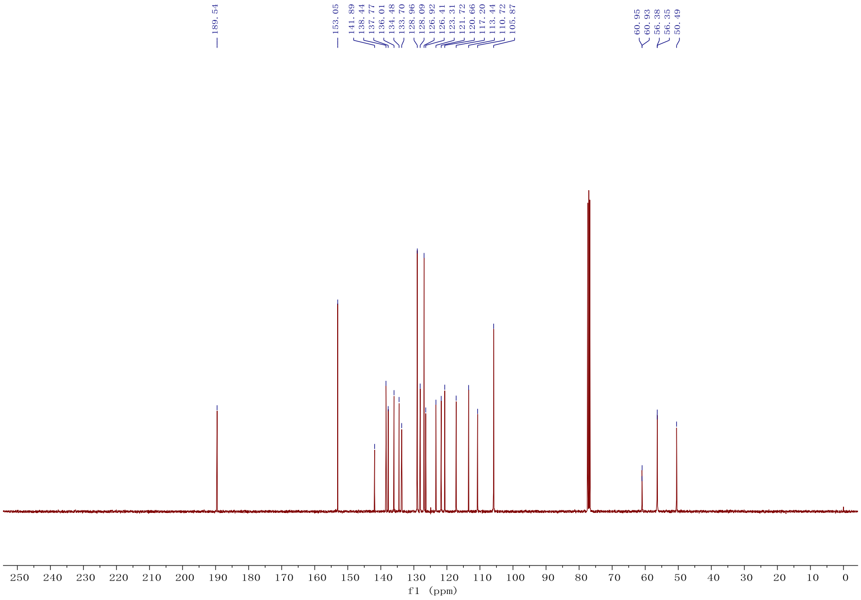

^{13}C NMR of Compound of **29d**

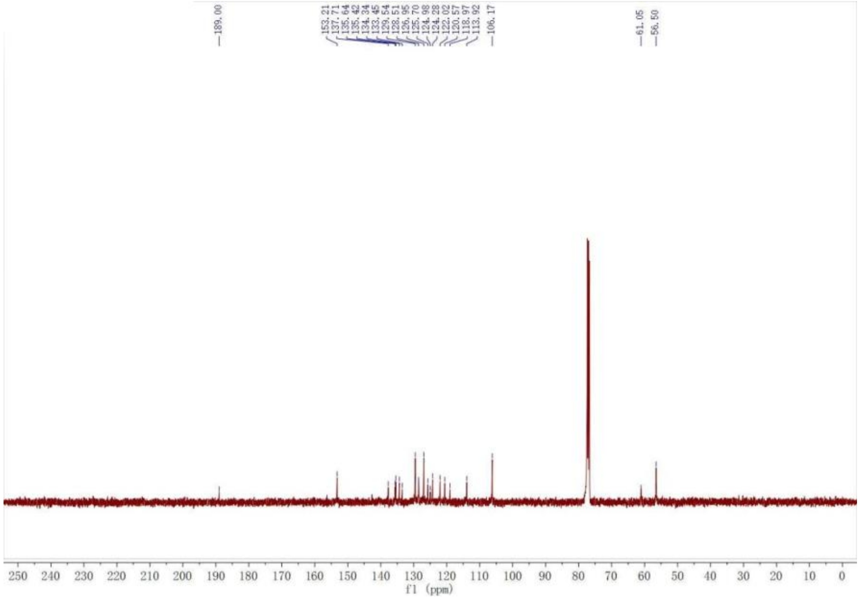

^{13}C NMR of Compound **29e**

4. The purity of potential compounds

Formatted: Font: 12 pt
Formatted: Font: 12 pt, Not Bold
Formatted: Centered

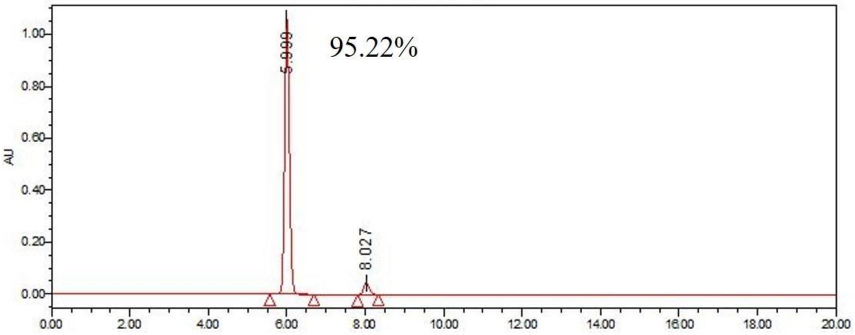

purity of compound **24d**

Formatted: Font: Not Bold

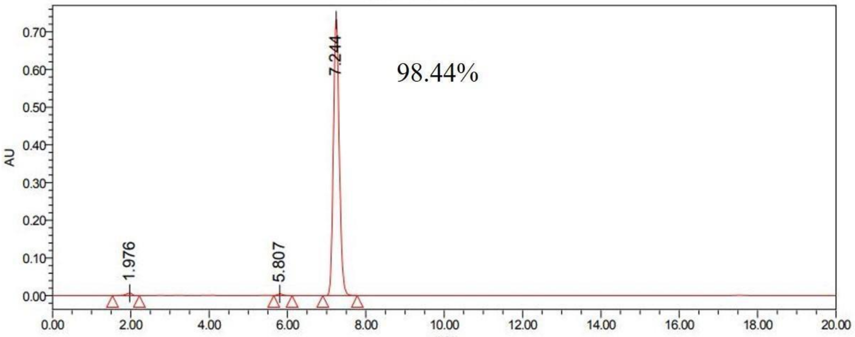

purity of compound **24e**

Formatted: Font: Not Bold

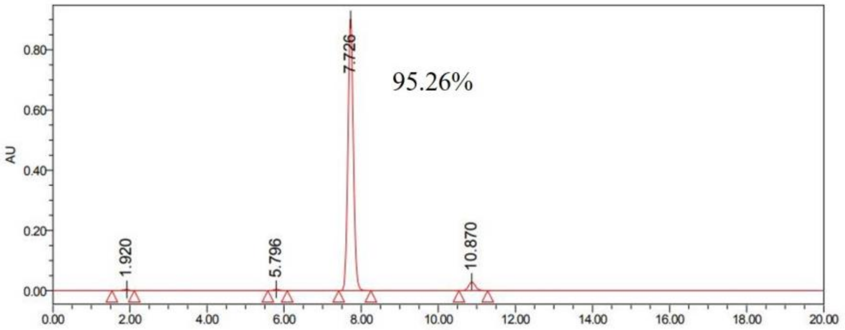

purity of compound **25d**

Formatted: Font: Not Bold

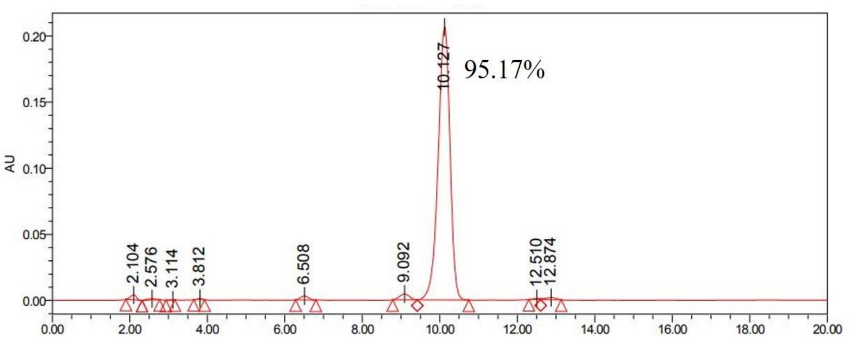

purity of compound **29e**

Formatted: Font: Not Bold

Formatted: Centered
